# Progressive aortic stiffness in aging C57Bl/6 mice displays altered contractile behaviour and extracellular matrix changes

**DOI:** 10.1038/s42003-022-03563-x

**Published:** 2022-06-17

**Authors:** Sofie De Moudt, Jhana O. Hendrickx, Cédric Neutel, Dorien De Munck, Arthur Leloup, Guido R. Y. De Meyer, Wim Martinet, Paul Fransen

**Affiliations:** Laboratory of Physiopharmacology, University of Antwerp, Antwerpen, Belgium

**Keywords:** Mechanisms of disease, Ageing, Arterial stiffening, Calcium signalling

## Abstract

Aortic stiffness is a hallmark of cardiovascular disease, but its pathophysiology remains incompletely understood. This study presents an in-dept characterization of aortic aging in male C57Bl/6 mice (2–24 months). Cardiovascular measurements include echocardiography, blood pressure measurement, and ex vivo organ chamber experiments. In vivo and ex vivo aortic stiffness increases with age, and precede the development of cardiac hypertrophy and peripheral blood pressure alterations. Contraction-independent stiffening (due to extracellular matrix changes) is pressure-dependent. Contraction-dependent aortic stiffening develops through heightened α_1_-adrenergic contractility, aberrant voltage-gated calcium channel function, and altered vascular smooth muscle cell calcium handling. Endothelial dysfunction is limited to a modest decrease in sensitivity to acetylcholine-induced relaxation with age. Our findings demonstrate that progressive arterial stiffening in C57Bl/6 mice precedes associated cardiovascular disease. Aortic aging is due to changes in extracellular matrix and vascular smooth muscle cell signalling, and not to altered endothelial function.

## Abstract (web summary)

A 24-month aging study in male C57Bl/6 mice reveals that aortic aging precedes cardiovascular disease and is due to changes in the extracellular matrix and vascular smooth muscle cell signaling.

## Introduction

As human life expectancy continues to grow, the incidence of age-related cardiovascular diseases (CVD) rises. According to the World Health Organisation, CVD has long since become the leading cause of death, resulting in an estimated 17.9 million deaths each year (i.e., 30% of global death)^1^. Arterial stiffening—defined as the impaired capacity of the large elastic arteries to smoothen pulsatile blood flow^2^—results in increased cardiac afterload, reduced coronary perfusion pressure, and pulsatile strain on the microcirculation. As such, arterial stiffness has gained much recognition as a hallmark and independent predictor of CVD^3–5^.

Elastic arteries display a distinctly non-linear stiffness-pressure relation, with a limited increase in stiffness in the physiological pressure range but exponential increase at high distending pressure^6,7^. Interestingly, despite pronounced variation in structural properties and vessel size across species, elastic modulus at mean physiological pressure is highly conserved across all vertebrate and invertebrate species with a closed circulatory system^8^, suggesting strong evolutionary pressure. Our research group previously showed that aortic vascular smooth muscle cell (VSMC) contraction modulates the elastic properties of the aorta^7^. At normal physiological pressure, maximal α_1_-adrenoreceptor-mediated contraction has been shown to increase ex vivo aortic stiffness by close to 100% at normal physiological pressure^7^, emphasizing the importance of this vasoactive pathway in the regulation of the elastic behaviour of the aorta. On the other hand, aortic contraction attenuates aortic stiffness when mean aortic distension pressure surpasses ~150 mmHg (where wall stress is transferred to incompliant collagen fibres) by disengaging collagen fibre alignment and shifting wall stress to contracted VSMC and elastin fibres^7^. This phenomenon is believed to constitute an important physiological system to regulate aortic stiffness in periods of acute hypertension (e.g., exercise, stress), when catecholamine levels typically increase alongside blood pressure. Furthermore, aortic contractile physiology was shown to be highly dependent on static^9^ and cyclic^10^ stretch stimulation, illustrating the complex interplay between arterial mechanostimulation, physiology, and biomechanics.

To investigate the role of aortic contraction in aortic (patho)physiology, our research group developed the Rodent Oscillatory Tension Set-up to study Arterial Compliance (ROTSAC), which is a novel organ chamber set-up which allows us to study biomechanical properties of arterial segments under dynamic conditions (i.e., physiologically relevant frequency and pressure oscillations)^11^. Using this organ chamber set-up, we previously reported altered contraction-dependent regulation of aortic biomechanics in mice after short-term angiotensin-II treatment^12^, in mice with autophagy-deficient VSMC^13,14^, and in mice with varying endothelial nitric oxide synthase (eNOS) expression^15^, demonstrating that dysfunctional contractile signalling in the aortic wall can underlie the pathology of aortic stiffening. We believe that an improved insight into the active processes that contribute to the pathophysiology of arterial stiffness may lead to novel treatment options for cardiovascular ageing and disease.

In the present study, a longitudinal cardiovascular characterization of spontaneously (i.e., age-dependent) ageing C57Bl/6 mice is presented to establish the temporal relation of aortic stiffness to associated CVD, i.e., cardiac hypertrophy and peripheral hypertension. Furthermore, an in-depth physiological and biomechanical investigation of the isolated ex vivo thoracic aorta was employed to identify the key mechanisms of spontaneous arterial stiffening. We demonstrated that aortic stiffening precedes peripheral blood pressure alterations and left ventricular hypertrophy in spontaneously ageing C57Bl/6 mice, underlining the importance of implementing arterial stiffness measurement as an early marker of cardiovascular ageing in standard cardiovascular care. With age, aortic tissue of C57Bl/6 mice showed heightened α_1_-adrenergic contractility, aberrant VGCC function, and altered intracellular VSMC calcium handling, indicating an important role of altered vasoactive physiology and contraction-dependent aortic stiffness regulation in the pathophysiology of arterial ageing. Interestingly, only limited age-dependent changes in endothelial dysfunction were observed, indicating that spontaneous arterial ageing in C57Bl/6 mice occurred primarily on the VSMC level.

## Results

### C57Bl/6 mice show typical properties of arterial ageing

**Fig. 1:**
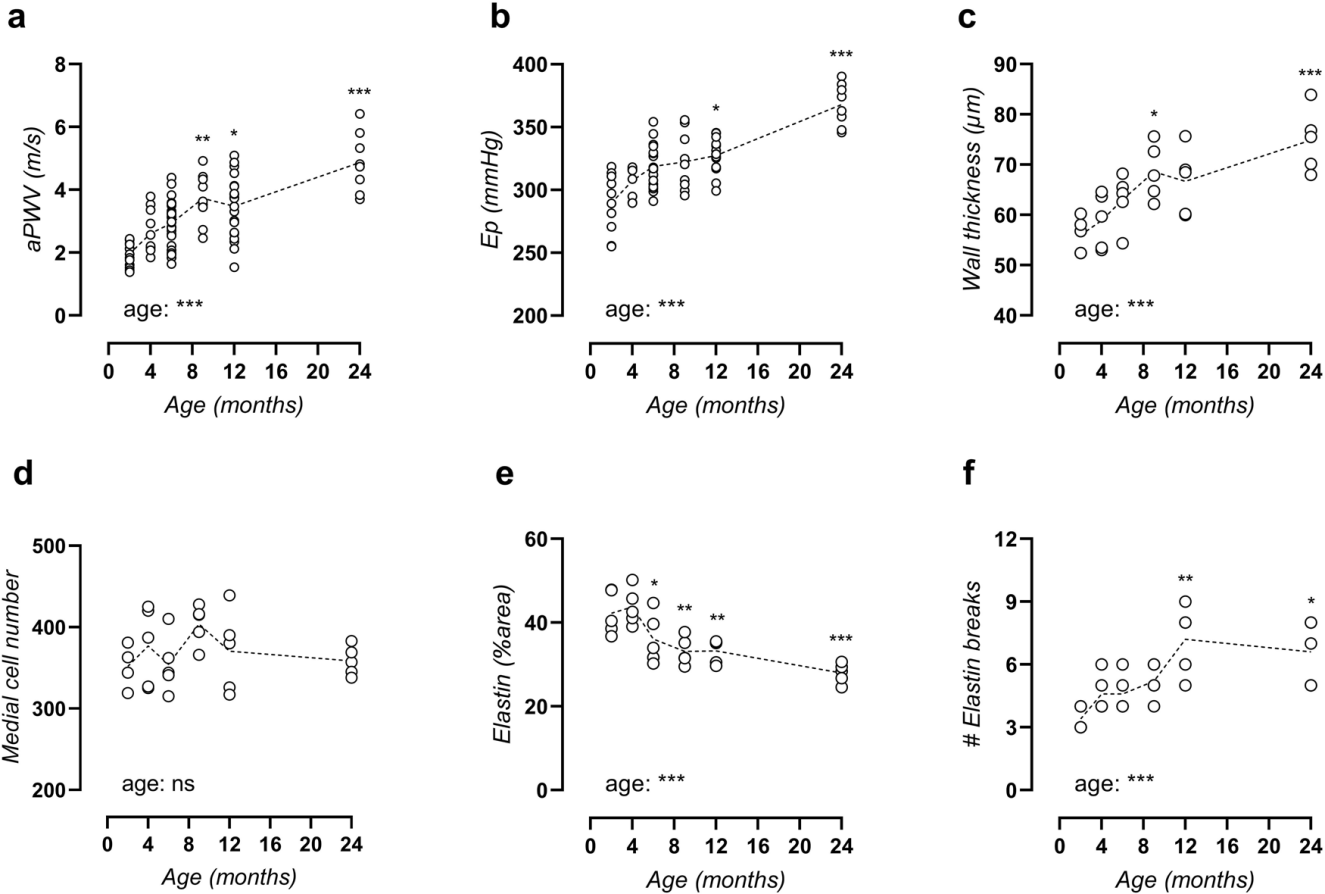
Age-related aortic stiffening in C57Bl/6 mice. Aortic stiffness was measured in vivo as aPWV (**a**) and ex vivo in the isolated thoracic aorta at isobaric conditions of 80–120 mmHg as Peterson modulus (E_p_, **b**). Wall thickness was determined on orcein-stained aortic tissue sections (**c**) and total medial cell number was counted on haematoxylin-stained aortic tissue sections (**d**). Orcein staining was used to quantify elastin positive area (**e**) and counting of elastin breaks (**f**). Each symbol represents an individual biological repeat, with *n* > 8 (**a**, **b**) and *n* = 5 (**c**, **f**). Statistical analysis using one-way ANOVA. Overall significance (bottom of graph) and post hoc significance vs. 4-month value (in graph) are listed. *p < 0.05, **p < 0.01, ***p < 0.001.

As expected, body weight gradually increased with age from 24.8 ± 0.4 g at 2 months to 35.6 ± 1.2 g at 24 months. Concomitantly, there was a non-significant trend towards increasing food and water intake, a significantly increased urinary output, and a significant age-dependent decrease of blood glucose levels (Supplementary Table 1). In vivo, aortic pulse wave velocity (aPWV) increased time-dependently, reached statistical significance versus adult 4-month-old mice by the age of 9 months (Fig. 1a), and increased to 250% of the initial value by the age of 24 months. To exclude confounding effects of possible haemodynamic factors and anaesthesia, aortic stiffness was also measured ex vivo in the isolated thoracic aorta as Peterson modulus (E_p_) in isobaric conditions (80–120 mmHg). Similar age-dependent stiffening was observed ex vivo, which reached significance versus adult 4-month-old mice at 12 months of age (Fig. 1b). These data suggest spontaneous development of progressive arterial stiffening with age. Histological analysis revealed increased wall thickness (Fig. 1c) without changes in VSMC number (Fig. 1d), implying cellular hypertrophy. Moreover, elastin content decreased with age (Fig. 1e) while elastin breaks accumulated (Fig. 1f) (representative image, Supplementary Fig. 1).

### Aortic age-dependent stiffening is more pronounced at higher distension pressure

**Fig. 2:**
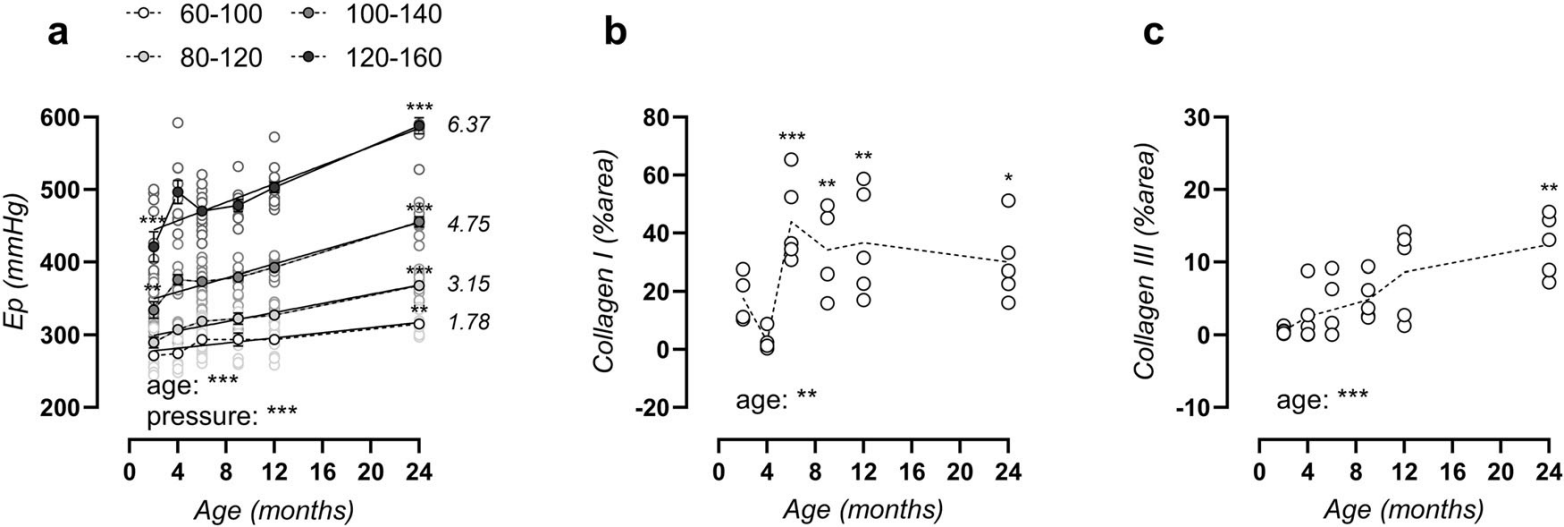
More pronounced aortic stiffening at high distending pressure corresponds to increased levels of types I and III collagen. Peterson modulus expressed in function of age for increasing distending pressure from 60–100, 80–120, 100–140, to 120–160 mmHg (**a**). Values of slope from linear regression analysis are listed on the right for each pressure. Histological assessment of area positivity for type I and II collagen (**b**, **c**). Data are listed as mean ± SEM (*n* > 8) (**a**) or each symbol represents an individual biological repeat (*n* = 5) (**b**, **c**). Statistical analysis using two-way ANOVA (**a**) or one-way ANOVA (**b**, **c**). Overall significance (bottom of graph) and post hoc significance vs. 4-month value (in graph) are listed. *p < 0.05, **p < 0.01, ***p < 0.001.

Next, E_p_ was studied over a broad physiologically relevant pressure range from hypotensive (60–100 mmHg) to normotensive (80–120 mmHg), borderline hypertensive (100–140 mmHg), and hypertensive (120–160 mmHg) distending pressures. The age-E_p_ relationship (Fig. 2a) displayed an advancing slope at increasing pressure, from 1.78 ± 0.39 (60–100 mmHg) to 3.15 ± 0.34 (80–120 mmHg), 4.75 ± 0.42 (100–140 mmHg), and 6.37 ± 0.78 (120–160 mmHg) mmHg/month (*p* = 0.002). These data demonstrate a more pronounced age-dependent aortic stiffening at high distending pressure. Since collagen is the primary load-bearing vessel-wall component at high pressure, this corresponds to the increased presence of types I and III collagen fibres (Fig. 2b, c) (representative image, Supplementary Figs. 2, 3).

### The C57Bl/6 mouse aorta shows increased contraction-dependent stiffening with age

**Fig. 3:**
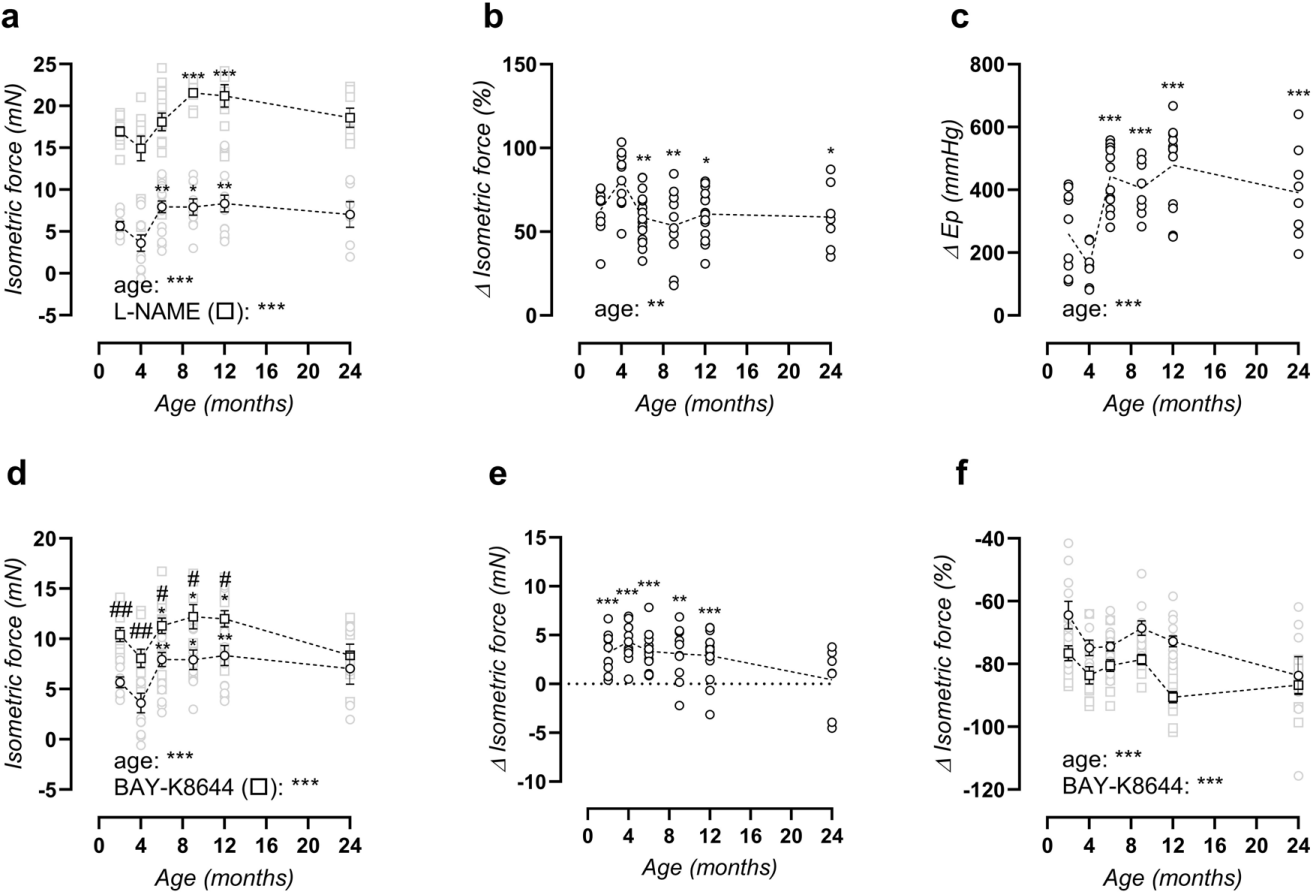
Increased PE-contractility and VGCC alterations. Maximal PE contraction in the absence (circles) and presence (squares) of L-NAME (**a**). Basal NO levels, quantified as the relative increase in PE contraction after addition of L-NAME (**b**). Contraction-dependent aortic stiffening by addition of 2 µM PE + 300 µm L-NAME at isobaric 80–120 mmHg pressure (**c**). Maximal PE contractions, as obtained from non-linear regression of the PE concentration-response curves, in the absence (circles) and presence (squares) of 30 nM BAY-K8644 (**d**), and calculated absolute BAY-K8644 effect (circles, **e**). Contraction-inhibition by diltiazem in the absence (circles) or presence (squares) of BAY-8644 (**f**). Statistical analysis using two-way ANOVA (**a**, **d**, **f**), one-way ANOVA (**b**, **c**) or one-sample *t*-test vs. 0 (**e**). Data are listed as mean ± SEM (**a**, **d**–**f**, *n* > 8) or each symbol represents an individual biological repeat (**b**, **c**, *n* > 8). Overall significance (bottom of graph) and post hoc significance vs. 4-month value (in graph) are listed. *p < 0.05, **p < 0.01, ***p < 0.001. In **d**, post hoc comparison for BAY-K8644 effect is listed as ^#^*p* < 0.05, ^##^*p* < 0.01.

Isometric contractions to phenylephrine (PE) were investigated in the absence and presence of N-Ω-Nitro-L-arginine methyl ester hydrochloride (L-NAME) to investigate the role contraction-depressing effect of basal NO. α_1_-Adrenergic stimulation of aortic segments resulted in an age-dependent increase in isometric contraction from 4 months onward, both in the absence and presence of L-NAME (Fig. 3a). Because statistical significance remained in the presence of nitric oxide synthase (NOS)-blocker L-NAME, relaxing basal nitric oxide (NO) levels in the absence of L-NAME seemed to be age-independent. Indeed, quantification of basal NO levels did not reveal a clear age-dependent depression in aged mice (although 4-month-old mice displayed a significant increase versus 6–24-month-old mice, Fig. 3b). Interestingly, a similar effect was observed on biomechanical aortic behaviour when inducing maximal VSMC contraction with PE + L-NAME, i.e., increased contraction-dependent aortic stiffening in the 6–24-month-old versus 2–4-month-old mice (Fig. 3c).

Voltage-gated calcium channel (VGCC) agonist BAY-K8644 significantly increased maximal PE-induced isometric contractions in mice aged 2–12 months old, whereas significance was lost at 24 months of age (Fig. 3d). The absolute increase of isometric force induced by maximal VGCC stimulation with BAY-K8644 was 3.4 ± 0.3 mN in 2–12-month-old mice, whereas no increase was observed in 24-month-old mice (Fig. 3e). Subsequent maximal VGCC inhibition with diltiazem revealed a clear age-dependent intensification of the VGCC contribution to PE-induced contraction (Fig. 3f). In the presence of BAY-K8644, diltiazem produced a larger inhibition, which can serve as a measure of maximal VGCC capacity. Maximal VGCC capacity was also significantly raised with age, since the diltiazem effect increased from 77 ± 2% (2 months) to 87 ± 3% (24 months, *p* = 0.021).

**Fig. 4:**
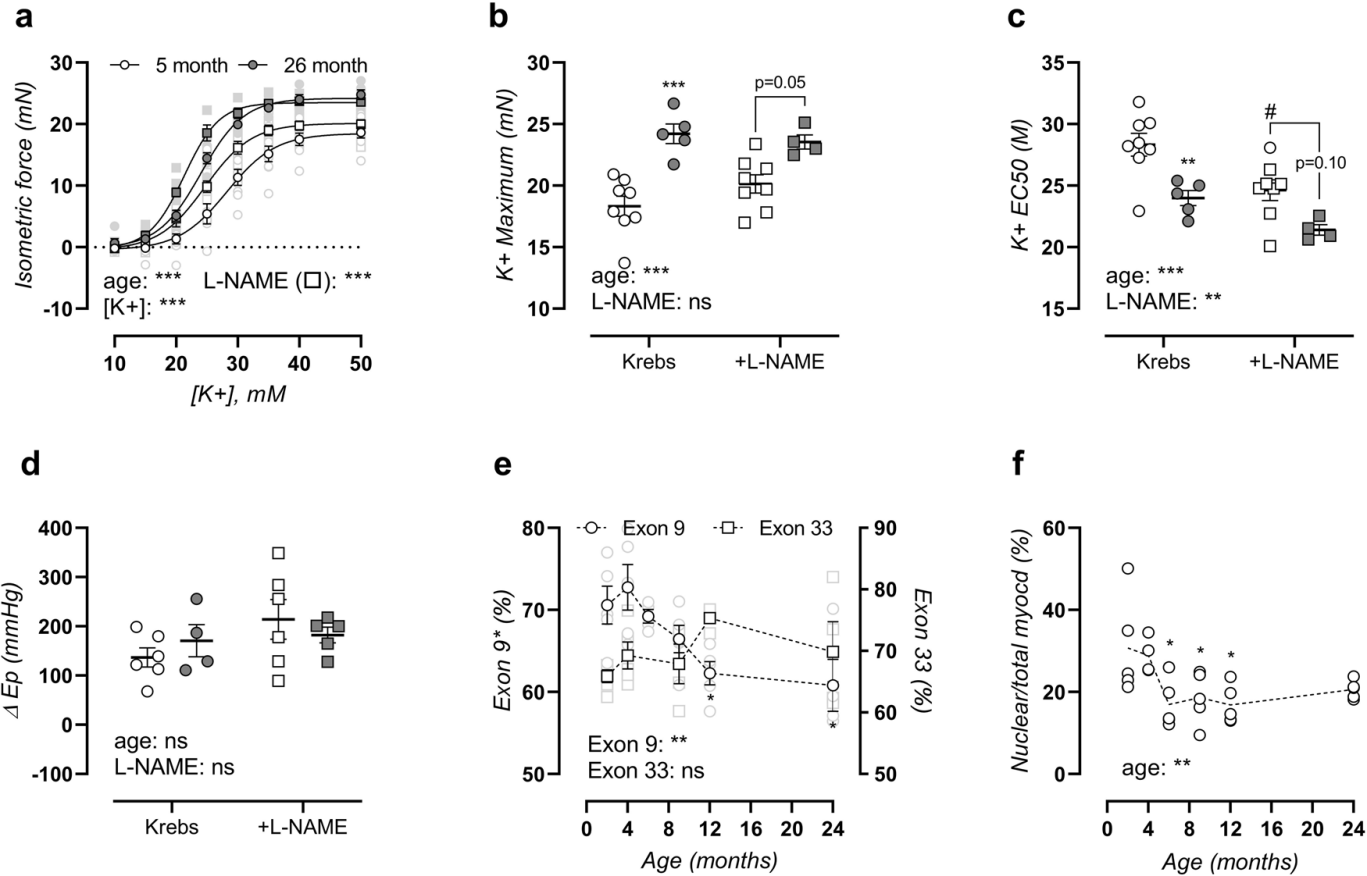
Functional and molecular VGCC phenotyping. Potassium concentration-response curves of 5- and 26-month-old mice were plotted in the absence (circles) and presence (squares) of L-NAME (**a**). Non-linear regression analysis was used to calculate the maximal effect (**b**) and EC_50_ value (**c**). 50 mM potassium-induced contraction-dependent change in Peterson modulus at calculated isobaric 80–120 mmHg distending pressure was measured in the absence and presence of 300 µM L-NAME (**d**). Splice variation was assessed for CaV1.2 exon 9* and exon 33, and quantification of the % mRNA with inclusion of exon 9* or exon 33 is shown (**e**). The fraction nuclear/total myocardin in the media was assessed using immunohistochemical staining, as a marker of VSMC phenotype regulation (**f**). Data are listed as mean ± SEM (**a**, **e**, *n* > 5) or each symbol represents an individual biological repeat (**b**–**d**, **f**, *n* > 5). Statistical analysis using three-way ANOVA (**a**), two-way ANOVA (**b**–**d**), or one-way ANOVA (**e**, **f**). Overall significance (bottom of graph) and post hoc significance vs. 4-month value (in graph) are listed (post hoc significance was not added in **a**). **p < 0.01, ***p < 0.001. In **c**, **d**, post hoc comparison for L-NAME effect is listed as ^#^*p* < 0.05.

Age-dependent VGCC activity was further assessed in a separate group of adult (5-month-old) and old (26-month-old) mice. Aortic segments were depolarized by elevating extracellular K^+^, which induces contraction mainly by activation of VGCC^16^. Depolarization of the aortic segments indeed resulted in markedly higher contractions in 26 months versus 5-month-old mice, both in the absence and presence of NOS-blocker L-NAME (Fig. 4a), indicating that increased depolarization-induced contractions were independent of basal NO production. Maximal contractions were highest in aged mice, especially in the absence of L-NAME (Fig. 4b). The sensitivity of the aorta to depolarisation by K^+^ was also age-dependent and half-maximal effective concentration (EC_50_) shifted from 28 ± 1 mM to 24 ± 1 mM K^+^ in the absence of L-NAME and from 25 ± 1 mM to 21 ± 0 mM K^+^ in the presence of L-NAME in the aorta of 5 and 26 months, respectively (Fig. 4c). 50 mM K^+^-induced contraction also resulted in distinct aortic stiffening, which was independent of age (Fig. 4d). In the presence of L-NAME (to suppress NO release and induce maximal contractions), K^+^-induced aortic stiffening was larger, although this effect seemed to be less pronounced in aged mice (non-significant trend, *p* = 0.1569). Post-transcriptional regulation of calcium voltage-gated channel subunit α1C (Cacna1C) by splice variation was assessed using reverse-transcriptase polymerase chain reaction (PCR) of CaV1.2 exon 9* and exon 33, demonstrating age-dependent suppression of exon 9* containing CaV1.2 from 71 ± 2% (2 months) to 61 ± 3% (24 months) messenger ribonucleic acid (mRNA) (Fig. 4e), whereas exon 33 splicing was unaltered (Fig. 4e) (representative image, Supplementary Fig. 4). Finally, VSMC phenotype was assessed by histological investigation of myocardin localization, showing a reduced nuclear myocardin fraction in the media of aged mice (Fig. 4f) (representative image, Supplementary Fig. 5). This indicates suppression of its transcriptional cofactor activity, and therefore loss of the normal VSMC contractile phenotype. Interestingly, the loss of nuclear myocardin was only significant in mice of 6–12 months of age, and again slightly improved in mice of 24 months of age, similar to the trend of maximal PE contractility.

### Endogenous but not exogenous NO-mediated relaxations show a desensitisation with age

**Fig. 5:**
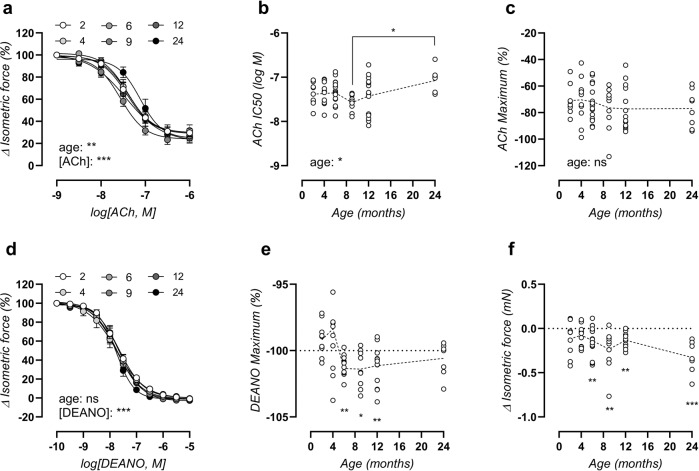
Ageing induces slight endothelial dysfunction and increased basal VSMC tone. ACh concentration-response curves of PE-precontracted aortic rings (**a**), with logIC_50_ value (**b**) and maximal effect (**c**) from non-linear regression analysis. Statistical analysis using two-way ANOVA (**a**) or one-way ANOVA (**b**, **c**). DEANO concentration-response curves of PE + L-NAME-precontracted aortic rings (**d**), with a calculated maximal effect (**e**) from non-linear regression analysis. Effect of replacing normal Krebs solution with 0Ca Krebs on isometric force (**f**). Data are listed as mean ± SEM (**a**, **d**, *n* > 8). Statistical analysis using two-way ANOVA (**a**, **d**), one-way ANOVA (**b**, **c**), or repeated one-sample *t*-test vs 100% (**e**) or 0 mN (**f**). Overall ANOVA significance (bottom of graph) and post hoc significance as indicated (above brackets) are listed (post hoc significance was not added in **a**, **d**). *p < 0.05, **p < 0.01, ***p < 0.001.

Aside from basal NO, which was age-independent as previously discussed (Fig. 3b), endothelial NO function was further assessed by eliciting acetylcholine (ACh)-induced relaxations in 2 µM PE-preconstricted aortic rings. ACh concentration-response curves (Fig. 5a) revealed a significant difference, which was due to late-onset desensitisation, as represented by a reduced sensitivity (half-maximal inhibitory concentration [IC_50_]) to ACh of −7.1 ± 0.1 log(M) in the 24-month-old mice versus −7.4 ± 0.1 log(M) in 2-month-old mice (Fig. 5b). A statistically significant difference in ACh IC_50_ was only obtained when comparing the 24-month values to the 9-month values, as illustrated in Fig. 5b. The maximal relaxation to ACh remained preserved with age, with an average −74 ± 2% relaxation across all ages (Fig. 5c).

Endothelium-independent relaxations were elicited by diethylamine NONOate (DEANO) concentration-response stimulation of 2 µM PE-preconstricted aortic rings, in the presence of 300 µM L-NAME to exclude effects of endogenously produced NO. The sensitivity of the aortic VSMC to exogenous NO (DEANO) was unaltered by age (Fig. 5d), verifying that the above-mentioned age-dependent desensitization to ACh represented reduced production of endothelial NO. The maximal effect of DEANO surpassed 100% relaxation from 6-months of age onward (Fig. 5e), evidencing a heightened basal VSMC tone in Krebs-Ringer solution in older animals. This was confirmed by the significant decrease in basal force when normal Krebs-Ringer solution was replaced by a solution lacking calcium (0Ca Krebs, Fig. 5f). The change in force by 0Ca Krebs reached a maximal −0.33 ± 0.06 mN in 24-month-old mice.

### Old C57Bl/6 mice display increased SR-mediated contractions

**Fig. 6:**
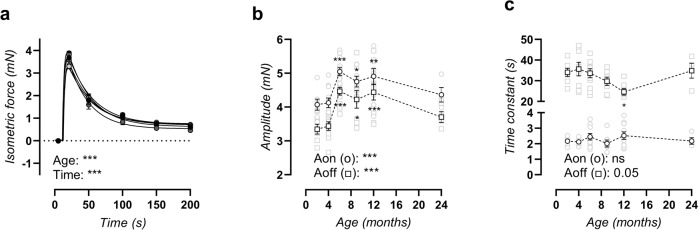
Altered transient SR-mediated contractions. A tracing of the transient SR-mediated contraction (**a**) was fitted with a bi-exponential regression equation encompassing the amplitude of the upward (A_on_) and downward (A_off_) phase (**b**), as well as the time constant of the upward (τ_on_) and downward (τ_off_) phase (**c**). Statistical analysis using two-way ANOVA (**a**) or one-way ANOVA (**b**, **c**). Data are listed as mean ± SEM (*n* > 8). Overall significance (bottom of graph) and post hoc significance vs. 4-month value (in graph) are listed (post hoc significance was not added in **a**). *p < 0.05, **p < 0.01, ***p < 0.001.

Transient sarcoplasmic reticulum (SR) Ca^2+^ release-mediated contractions were studied by α_1_-adrenergic stimulation of aortic segments in 0Ca Krebs solution, which prevented extracellular calcium influx. Phasic contractions were age-dependent (Fig. 6a). Bi-exponential regression of the upward (calcium release) and downward (calcium removal) phase revealed an increased amplitude of both phases in mice aged 6–12 months old, which is again attenuated in the 24-month-old mice (Fig. 6b). The time constant of the upward phase showed no age-dependent changes, whereas the time constant of the downward phase showed a decline from 6 to 12 months of age, which was again attenuated in 24-month-old mice (Fig. 6c).

### Cardiac hypertrophy and late-onset peripheral pulse pressure elevation

**Fig. 7:**
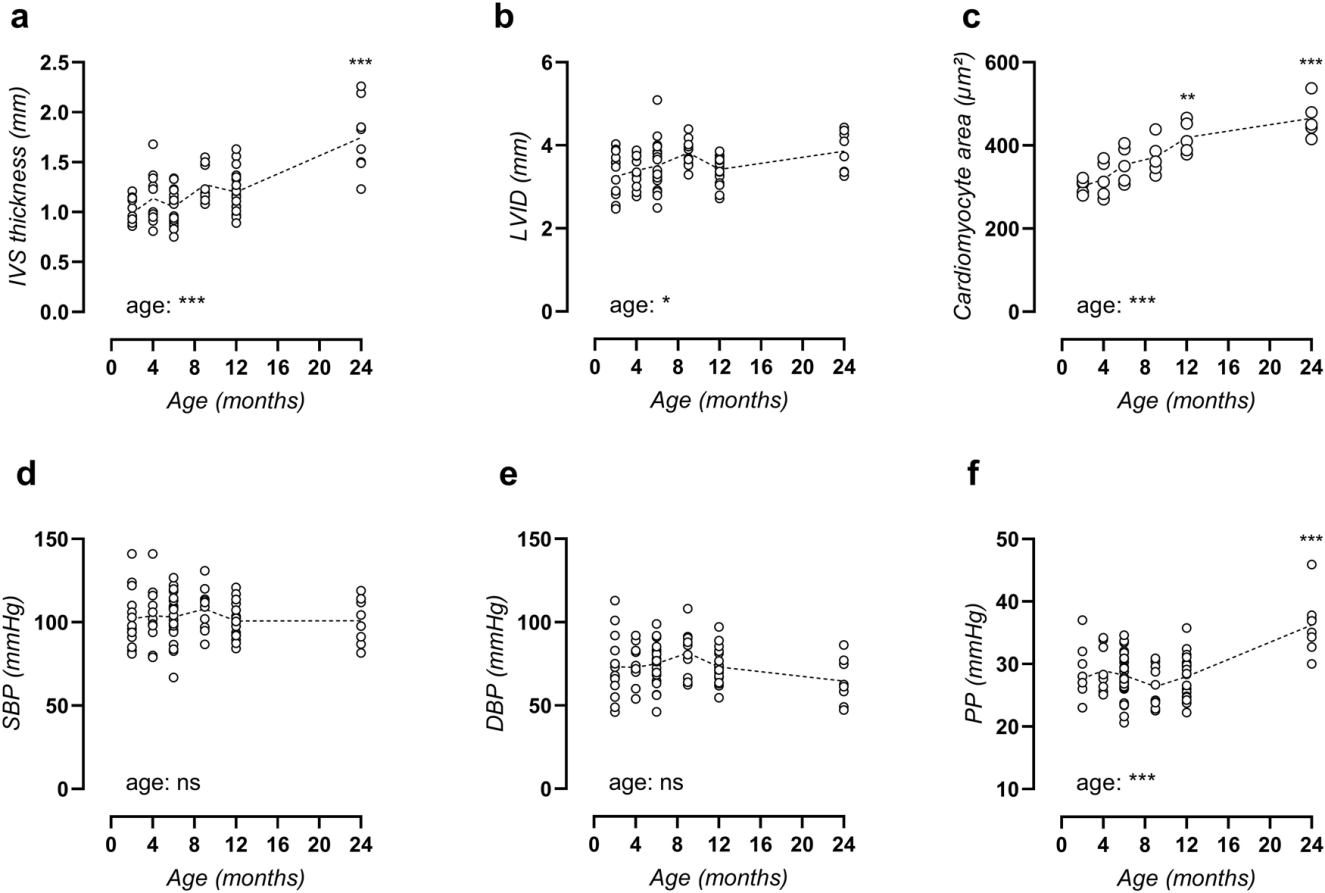
Arterial-stiffness precedes cardiac disease and peripheral blood pressure alterations. Echocardiograms were used to assess interventricular septum (IVS) thickness (**a**) and left ventricular internal diameter (LVID, **b**). Cardiomyocyte cross-sectional area was studied using immunohistochemical staining for laminin (**c**). Peripheral systolic blood pressure (SBP, **d**), diastolic blood pressure (DBP, **e**) and pulse pressure (PP, **f**) were measured. Each symbol represents an individual biological repeat (*n* > 8). Statistical analysis using one-way ANOVA. Overall significance (bottom of graph) and post hoc significance vs. 4-month value (in graph) are listed. ***p < 0.001.

Echocardiograms revealed significant cardiac hypertrophy with age, which included significantly increased interventricular septum (IVS) thickness at 24 months of age (Fig. 7a). Slight dilation of the left ventricular (LV) lumen was also observed (Fig. 7b), indicating eccentric cardiac hypertrophy. Hypertrophy was confirmed on the cellular level using histological assessment of cardiomyocyte cross-sectional area (Fig. 7c) (representative image, Supplementary Fig. 6). Other cardiac parameters included unaltered heart weight and conscious heart rate, increased LV mass, normal ejection fraction (EF), and normal fractional shortening (FS). In addition, diastolic dysfunction was observed as an increased isovolumic relaxation time (IVRT), although no other indications of diastolic heart disease were present (Supplementary Table 2).

The temporal development of aortic stiffness and hypertension was studied by peripheral blood pressure (BP) measurement. Remarkably, systolic blood pressure (Fig. 7d) and diastolic blood pressure (Fig. 7e) both remained unchanged until the age of 12 months, with an mean (SEM) systolic and diastolic blood pressure of 103.0 ± 14.0 mmHg and 74.8 ± 13.5 mmHg, respectively. At 24 months, mean (SEM) blood pressure shifted to 100.8 ± 13.7 mmHg and 64.6 ± 13.8 mmHg, respectively, thus displaying a non-significant decrease in diastolic blood pressure. This resulted in a markedly heightened pulse pressure, which was significantly increased in the 24-month-old mice as compared to all other ages (Fig. 7f).

## Discussion

This study demonstrates that spontaneously ageing C57Bl/6 mice develop increased aortic stiffness by the age of 9 months, thus preceding cardiac hypertrophy and peripheral blood pressure alterations which were observed at 24 months. Aortic stiffness was both contraction-independent (with marked ageing especially at high distending pressure), and contraction-dependent (heightened α_1_-adrenergic contractility). Aortic stiffening further coincides with aberrant VGCC function, i.e., altered alternative splicing, increased VGCC activation, elevated VGCC contribution to α_1_-adrenergic contractions, heightened VGCC capacity, and raised potassium-induced contractions. Furthermore, VSMC ageing promotes increased baseline contractile tone and changed intracellular calcium handling. On the endothelial level, ageing effects are limited to a modestly decreased sensitivity to ACh-induced relaxation.

The present study included mice of 2, 4, 6, 9, 12, and 24 months of age. This corresponds to equivalent human ages of 16, 23, 30, 36, 42.5, and 69 years, respectively^17,18^. As follows, the study includes most mouse life phases including juvenile (2-month old), mature adult (4- and 6-month old), middle-aged (9- and 12-month old), and old (24-month old)^17,18^, and the study thus describes both the maturation (from 2 to 4 months of age) and ageing (from 4 to 24 month of age) of male C57Bl/6 mice. The results in this study demonstrate that significant aortic ageing (including increased collagen and decreased elastin content) occurs early in the ageing process, starting at 6 months of age. In (healthy) humans, pulse wave velocity (PWV) also increases early in adult life^19–23^, with a linear age-dependent increase in PWV in humans aged 10–80 years old and a PWV of ~5–9 m/s (young-old). Interestingly, PWV shows significant changes already at 20–29 years of age. Data from the present study also demonstrate a linear increase in PWV in 2–24-month-old mice. Therefore, spontaneous arterial ageing in C57Bl/6 mice follows a similar pattern as reported for humans. Furthermore, thoracic aorta stiffness of C57Bl/6 mice was studied ex vivo, as to assess aortic stiffness in controlled and isobaric conditions. These experiments confirm that the increased PWV represents biomechanical changes of the aortic wall itself (rather than confounding haemodynamic factors) and suggest that aorta-targeting interventions may delay age-dependent CVD initiation.

Recent ageing studies have advanced our understanding of aortic stiffness pathophysiology, and identified novel therapeutic targets. An example of such promising treatment strategy, suggested by Kim et al.^24^, is mineralocorticoid receptor (MR) antagonism. Indeed, both genetic deletion and pharmacological inhibition of MR in VSMCs attenuated age-related aortic stiffening and fibrosis. Another example is the use of stem-cell-derived extracellular vesicles to reverse age-associated aortic stiffness and hypertension through activation of the sirtuin type 1 (SIRT1)/eNOS/AMPKα axis^25^. Genetic SIRT1 overexpression in mice also inhibited aortic stiffening by preventing aortic collagen and advanced glycation end-product accumulation with age. Even in 24-month-old mice, SIRT1 activation significantly improved aortic stiffness and blood pressure^26^. Both MR antagonism and SIRT1 agonism are therefore believed to target aortic ageing by affecting fibrosis and extracellular matrix (ECM) organisation. The present study described marked aortic ECM changes with age, including increased collagen and decreased elastin content, which represent a hallmark ECM response to ageing^27–29^. Interestingly, although collagen types I and III (which are the main fibril-forming collagens involved in imparting strength to the vessel wall^30,31^) were both increased with age, different trends were observed during the ageing process. For collagen type III, a gradual increase with age was observed (similar to the observed increase in passive aortic stiffness), whereas collagen type I was increased in 6–24-month-old mice versus 2–4-month-old mice (similar to the observed increase in aortic contractility). These results suggest a variant role of collagen types I and III in aortic physiology and ageing. Even though the specific role of MR or SIRT1 was not investigated in this study, the above-mentioned findings prove that ECM and fibrotic alterations can be reversed by therapeutic intervention, even at advanced age.

Aside from cell-ECM changes, the present study suggests that heightened α_1_-adrenoreceptor-mediated contractility with age—as confirmed by other independent ageing studies^32,33^—plays an important role in contraction-dependent stiffening of the aged aorta. Heightened PE-induced contractions were observed from 6 months onward, although a late-onset attenuation in the presence of NOS-blocker L-NAME (i.e., maximum contracted) was remarked in 24-month-old mice. Previous work of our research group has established the importance of VSMC contraction in the active regulation of aortic stiffness^7^, indicating that heightened contractile behaviour of the aged aorta might impair the active regulation of the aortic pressure-stiffness relationship. Lysyl oxidase-like 2 (LOXL2) haploinsufficiency has been shown to attenuate PE-contractions in aged mice, accompanied by protection against age-dependent aortic stiffening, blood pressure alterations, and ECM matrix reorganisation^33^. This confirms the relevance of dysfunctional VSMC contractile behaviour in arterial ageing.

In chronic arterial stiffness and hypertension, ion channel remodelling in VSMC leads to depolarisation^34^, which may result in “premature” VGCC activation. VGCC represent the foremost calcium entry pathway in VSMC, illustrated by the pronounced phenotype of smooth muscle cell (SMC)-specific Cacna1C knockout mice (i.e., disestablished myogenic tone and severe hypotension)^35^. Accordingly, increased VGCC activity in cardiovascular ageing has been reported in cardiomyocytes^36,37^ and VSMC of various arterial beds (e.g., aorta^34,38^, coronary arteries^39^, mesenteric arteries^40^). On the other hand, decreased VGCC expression and activity with cardiovascular ageing were also reported^41^, and the exact role of VGCC in CVD is thus up for debate. In the present study, a distinct role for VGCC dysfunction was revealed, which includes an increased contribution of VGCC to α_1_-adrenergic contraction, a heightened VGCC capacity, an increased VGCC activation state, elevated depolarisation-induced (KCl) contractions, and an altered post-transcriptional splice variant expression. Splice variation acts as a predominant regulatory mechanism of VGCC activity. The mouse Cacna1C gene consists of 49 exons, 20 of which undergo alternative splicing, and accounting for 50 expressed splice isoforms in cardiac and smooth muscle tissues^42^. In VSMC, splice variation is confined to three mutually exclusive (1b/c, 21/22 and 31/32) and two alternate (9* and 33) exons^43^, of which several isoforms have been reported in hypertension pathophysiology. Particularly exon 9* and 33 are broadly studied. Increased exon 9* inclusion is reported in the mesenteric artery of spontaneously hypertensive rats (SHR)^44^, and results in a hyperpolarized shift in voltage-dependent activation toward the physiological arterial voltage range^45^ which may contribute to the elevated vasoconstrictive response of SHR arteries. Hence the increased splicing of exon 9* in spontaneously aged C57Bl/6 mice is at variance with the distinct observation of heightened high K^+^-induced and α_1_-adrenoreceptor-mediated contractility^46,47^. The VGCC isoform with spliced exon 9* was further shown to display a decreased sensitivity to inhibition by diltiazem (i.e., >2-fold increase in IC_50_)^48^. Although the diltiazem concentration in the present study (35 µM) induced maximal inhibition in healthy aortic segments, it could be hypothesised that desensitization of the ion channel with age resulted in an underestimation of the inhibitory effect. Thus, the age-dependent increase in VGCC contribution to α_1_-adrenergic contractions could be even stronger than initially observed. On the other hand, a parallel age-dependency was observed between exon 9* splicing and the effects VGCC agonist BAY-K8644 on contraction. Because both affect the voltage-dependent activation of VGCC, these data at least suggest that the voltage-dependency of VGCC is altered with increasing age.

Unexpectedly, the present study found that most physiological changes with age involve VSMC function, whereas endothelial NO production was largely unaffected. ACh-stimulated relaxations showed a slight desensitisation at 24 months of age, whereas basal NO production remained normal altogether. This opposes consensus clinical knowledge which denominates endothelial dysfunction as a cardiovascular ageing hallmark^49–51^. Preclinical studies in accelerated ageing models have indeed identified endothelial dysfunction as a key mechanism of ageing^52^. Similarly, another recent ageing study demonstrated impaired NO function during ageing of C57Bl/6 mice, which was accelerated by Western-type diet and ameliorated by aerobic exercise^53^. Severe premature ageing in a murine model of Hutchinson–Gilford progeria syndrome (HGPS) promotes increased aortic stiffness both in vivo^54^ and ex vivo^54,55^. Interestingly, by cell type-specific expression of the HGPS mutation in endothelial cells (EC) or VSMC, it was shown that the arterial ageing phenotype was caused by functional changes in VSMC rather than EC^55^, in line with our findings that spontaneous ageing mostly occurs on the level of VSMC. Nonetheless, dietary supplementation with nitrates reverses the HGPS aortic phenotype, indicating that improved endothelial function may still compensate for aberrant VSMC function^55^.

As the leading world health risk, hypertension affects 40% of adults worldwide and results in 9.4 million annual deaths^56^. Consequently, the pharmaceutical industry has greatly invested in the development of antihypertensive dugs leading to a marked evolution in blood pressure-lowering drugs. Nevertheless, target blood pressure control under antihypertensive treatment is only achieved in 53% of patients^57^. Other therapeutic strategies should thus be explored. It has long been discussed whether arterial stiffness is the cause or consequence of age-related hypertension^58^, and whether it might represent a valuable therapeutic target for patients with uncontrolled hypertension. On the one hand, arterial stiffening gives rise to early return of reflected pressure waves leading to systolic BP augmentation and could thus be argued to cause hypertension directly^59^. Conversely, the non-linear elastic behaviour of the aorta shows that arterial stiffness acutely increases at high distending pressure^7^, and chronic exposure to high blood pressure is known to promote vessel wall changes (e.g., medial hypertrophy and ECM synthesis) which lead to chronic arterial stiffening. Since aortic stiffness is therefore both cause and consequence of hypertension, they ultimately represent a positive feedback loop, which encompasses an important clinical challenge^60^.

Growing evidence supports aortic stiffening as contributing factor in the development and progression of hypertension. This includes reports stating that aortic stiffness can precede the onset of hypertension in preclinical models^61,62^, as well as epidemiological findings suggesting that elevated aortic stiffness can predict future blood pressure changes whereas blood pressure has no predictive value for future PWV^63^. Such studies do not only underline the therapeutic potential of arterial stiffness-targeting drugs, but endorse the implementation of aortic stiffness measurement in standard cardiovascular care of at-risk patients. This was first recommended in 2007 by the European Society of Hypertension/European Society of Cardiology (ESC/ESH) guidelines^64^, which advocate for PWV assessment in cardiovascular risk evaluation. Recommendations notwithstanding, implementation of PWV measurement in CVD prevention and management has not yet found its way into general clinical practice, even though a 1 m/s increase in carotid-femoral PWV seriously aggravates the risk for cardiovascular events and all-cause mortality^65^.

The present study complements literature findings with the observation that elevated PWV precedes peripheral blood pressure alterations in spontaneous cardiovascular ageing by 15 months, as confirmed by other ageing studies^33,66^, affirming the use of aortic stiffness as an early marker in CVD monitoring as recommended by the ESC/ESH guidelines. The present study measured blood pressure using the tail-cuff method. Although this is not a gold standard method for blood pressure assessment, it allows for measurement at a peripheral site, which is more relatable to the clinical setting and indicative of the risk of pulsatile damage to the peripheral organs. Although in the present study, peripheral blood pressure changes are limited to elevated pulse pressure, without clear systolic hypertension, other ageing studies have described spontaneous peripheral systolic blood pressure increase with advanced age in mice^33^. Biomechanical and histological data in this study are in favour of treatment strategies that target cell-ECM interactions, such as MR antagonism or SIRT1 agonism. Moreover, ex vivo physiological investigation revealed other key players in the ageing process, such as heightened α_1_-adrenergic contractility and dysregulation of VGCC activity, which might represent new therapeutic targets in CVD management.

It needs to be emphasized that the presented data in this manuscript only includes assessments of structural aortic stiffness of the entire arterial segment, rather than assessments of material stiffness of aortic tissue, since no correction was made for aortic wall thickness during ex vivo biomechanical testing. Rachev et al.^67^ previously reported that a 60% increase in wall thickness only increased E_p_ by 16%. Since the present manuscript reports a 34% increase in aortic wall thickness and a 27% increase in E_p_ in 24-month-old mice (versus 2-month-old mice), the presented data suggest that the observed differences in ex vivo aortic stiffness were not solely the result of aortic wall thickening and that age-related aortic tissue stiffening may also occur in spontaneously ageing C57Bl/6 mice. This could represent an interesting topic for future research.

In conclusion, progressive arterial stiffening was observed in ageing male C57Bl/6 mice, with significantly increased PWV and ex vivo Peterson modulus at 9 and 12 months of age, respectively, thus preceding associated cardiac hypertrophy and peripheral blood pressure changes, which developed by 24 months of age. Thoracic aorta reactivity and biomechanical testing revealed both contraction-independent stiffening due to ECM changes, and contraction-dependent stiffening as a result of heightened α_1_-adrenergic contractions and aberrant VGCC function. Minimal changes in endothelial NO signalling were observed, suggesting that spontaneous arterial ageing in the studied male C57Bl/6 mice was due to ECM and VSMC signalling changes rather than altered EC function. In light of the high prevalence of arterial stiffness and hypertension in the ageing population, this study advocates for measurement of arterial stiffness in cardiovascular care and pinpoints possible new therapeutic interventions to include VSMC-ECM interactions or adrenergic and VGCC signalling pathways.

## Methods

### Laboratory animals and tissue collection

All animal experiments were approved by the Ethical Committee of the University of Antwerp and conducted in accordance to the Guide for the Care and Use of Laboratory Animals, published by the National Institutes of Health (NIH Publication No. 85-23; Revised, 1996). Randomization and blinding were not applicable to this study. All mice were bred and housed in the animal facility of the University of Antwerp, with a 12 h/12 h light-dark cycle and had free access to water and standard chow (Ssniff® Rat/Mouse maintenance 10 mm, V1534-000). For the main experiment, male C57Bl/6 mice were used at the age of 2 (*n* = 14), 4 (*n* = 11), 6 (*n* = 25), 9 (*n* = 10), 12 (*n* = 20), and 24 (*n* = 8) months old, which were each measured once at their respective end ages to avoid the influences of repeated measurements. One week before sacrifice, in vivo parameters were measured, including body weight and venous nonfasting blood glucose level, 24 h metabolic cages for recording of food and water intake and urinary production, and various cardiovascular parameters. When mice were under deep anaesthesia (pentobarbital sodium, 75 mg/kg ip; Sanofi, Belgium), mice were euthanized by perforation of the diaphragm. The thoracic aorta and heart were carefully removed and the aorta was stripped of adherent tissue. Next, aortic rings of 2 mm width were cut starting at the diaphragm. Of these, two segments were used for ex vivo isometric reactivity studies, two segments for ex vivo assessment of biomechanical aortic properties, and one segments was fixed in 4% formaldehyde for histological staining. The most proximal thoracic aorta segment, just distally from the left subclavian branch of the aortic arch, was embedded in optimal cutting temperature (OCT) medium, and stored at −80 °C for RNA isolation. The heart was also isolated and longitudinally cut in halve, randomly assigned for formaldehyde fixation or snap-freezing. In a follow-up experiment, a separate group of young (5 months, *n* = 8) and aged (26 months, *n* = 5) mice were used for assessment of isometric potassium concentration-response curves.

### In vivo cardiovascular measurements

One week before sacrifice, mice underwent cardiovascular tests, to investigate the time-dependent development or age-related aortic stiffening, peripheral blood pressure alterations and cardiac disease. For in vivo cardiovascular measurements, *n* = 14 (2 month), *n* = 11 (4 month), *n* = 25 (6 month), *n* = 10 (9 month), *n* = 20 (12 month) and *n* = 8 (24 month) biological replicates were included. Peripheral blood pressure was measured with the CODA tail-cuff method^68^. A pressure-volume sensor was attached distally to an occluding cuff to the tail of conscious restrained mice for blood pressure recording. Systolic and diastolic blood pressure were measured on three consecutive days, of which the final measurement was used. Next, transthoracic echocardiograms were acquired in anesthetized mice (1.5–2.5% isoflurane v/v (Forene, Abbvie)) using high-frequency ultrasound (Vevo2100, Visualsonics). Heart rate was maintained at 500 ± 50 beats/min and body temperature between 36 and 38 °C. M-mode images were obtained for LV function evaluation on short-axis view, including measurement of IVS thickness and LV lumen diameter on three consecutive respiratory cycles. FS, EF, LV mass, and stroke volume (SV) were calculated. On a four-chamber view, diastolic heart function parameters were assessed using pulsed wave Doppler analysis of blood flow through the mitral valve, which allows for measurement of the E wave, A wave, isovolumic relaxation time and deceleration time, and calculation of the E/A ratio. Finally, aPWV was measured using the method of Di Lascio et al.^69^. B-mode images of aortic diameter and pulsed wave Doppler analysis of velocity were acquired and averaged over several cardiac cycles. aPWV was calculated as dV/2dln(D) (with dV, velocity change; and dln(D), the variation of the natural logarithm of diameter).

### Isometric reactivity studies

To investigate age-dependent alterations in aortic physiology, 2-mm aortic rings were mounted between two parallel wire hooks in a 10-mL organ bath containing Krebs-Ringer solution (composition (mM): NaCl 118; KCl 4.7; CaCl_2_ 2.5; KH_2_PO_4_ 1.2; MgSO_4_ 1.2; NaHCO_3_ 25; CaEDTA 0.025; glucose 11.1). The solution was continuously heated to 37 °C and aerated with a 95% O_2_/5% CO_2_ gas mixture to maintain a pH of 7.4.

In the main ageing experiment (2–24 months of age), a fixed preload of 20 mN was applied to approximate normal physiological stretch at a mean blood pressure of 100 mmHg^9^, and isometric contractions and relaxations were measured by means of a Statham UC2 force transducer (Gould, USA). For isometric reactivity studies, *n* = 10 (2 months), *n* = 9 (4 months), *n* = 18 (6 months), *n* = 9 (9 months), *n* = 14 (12 months) and *n* = 8 (24 months) biological replicates were included. Contractions were induced by concentration-response stimulation with α_1_-adrenergic agonist PE (3 nM to 10 µM), in the absence and presence of VGCC agonist BAY-K8644 (30 nM). Subsequently, VGCC were blocked with 35 µM diltiazem to assess the contribution of VGCC to PE-induced contractions. Endothelium-dependent and -independent relaxations were induced by concentration-response stimulation with ACh (3 nM to 1 µM) and DEANO (0.3 nM–10 µM), respectively, in PE-precontracted aortic rings. For DEANO-induced relaxations, 300 µM L-NAME (NOS blocker) was also added to the organ bath to exclude effects of endogenously produced NO. Basal NO levels were quantified as the relative difference in PE-induced contractile force in the absence and presence of L-NAME. Finally, to avoid extracellular calcium influx, Krebs-Ringer solution was replaced by a solution lacking calcium (0Ca Krebs), and a transient inositol 1,4,5-triphosphate (IP_3_)-mediated contraction was induced by 2 µM PE as previously described^16^. IP_3_-mediated contractions were studied in parallel in two aortic rings and averaged for the analysis.

In a follow-up experiment (separate group of 5- and 26-month-old mice), both isometric and isobaric measurements were performed in ROTSAC organ baths, allowing for calibration of aortic segments and resulting application of a preload corresponding to a calculated pressure of 100 mmHg for each individual aortic ring. This preload was significantly increased from 26.7 ± 0.8 (*n* = 6) to 30.0 ± 0.5 mN (*n* = 4) for 5- and 26-month-old mice, respectively. Receptor-independent contractions were elicited using Krebs-solutions with increasing concentrations of potassium (5.9 to 50 mM K^+^). These Krebs-solutions were prepared by iso-osmotic replacement of sodium chloride with potassium chloride.

All measurements were performed after steady-state conditions were reached. All concentration-response curves were fitted with a non-linear 4-parameter equation, to obtain values for maximal effect and EC_50_ or IC_50_.

### Measurement of ex vivo aortic stiffness

To investigate age-dependent alterations in aortic biomechanical properties, 2-mm aortic rings were mounted in a ROTSAC, between two parallel wire hooks in a 10-mL organ bath containing Krebs-Ringer solution. The upper wire hook was connected to a force-length transducer, and segments were continuously stretched between alternating preloads corresponding to the “systolic” and “diastolic” transmural pressures at a physiological frequency of 10 Hz to mimic the physiological heart rate in mice (600 bpm)^11^. At any given pressure, calibration of the upper hook allowed for the calculation of the diastolic and systolic vessel diameter (mm) and E_p_. E_p_ was defined as the pulse pressure divided by the relative diameter change (E_p_ = D_0_*ΔP/ΔD), and can be interpreted as the pressure change that is required to increase aortic diameter by 100%. For ex vivo biomechanical studies, *n* = 10 (2 month), *n* = 9 (4 month), *n* = 18 (6 month), *n* = 9 (9 month), *n* = 14 (12 month) and *n* = 8 (24 month) biological replicates were included. Aortic stiffness was always assessed in isobaric conditions, and measured at oscillating pressures of 60–100, 80–120, 100–140 and 120–160 mmHg. Corresponding preloads were applied consecutively on each aortic segment at increasing distending pressure as shown in the representative tracing in Supplementary Fig. 7. Contraction and relaxation of vessel segments were elicited as described above to assess different players in active contraction-dependent aortic stiffening.

### Histology

To investigate age-dependent structural changes in aortic and cardiac tissue, histological analyses were performed. Aortic and cardiac tissue were fixed for 24 h in 4% formalin solution (BDH Prolabo), and subsequently dehydrated in 60% isopropanol (BDH Prolabo), followed by paraffin-embedding. Aortic media thickness was measured on orcein-stained sections of the aorta, which allows for accurate assessment of the inner and outer border of the media layer. Orcein staining was further used to assess elastin content and for counting elastin breaks. Collagen composition of the media was ascertained by immunohistochemical staining with an anti-collagen I (Abcam, ab21286), anti-collagen III (Chemicon, MAB1343), and anti-collagen IV (DAKO, M0785) antibodies, and VSMC phenotype regulation was assessed by immunofluorescent staining with an anti-myocardin (Sigma, sab4200539) antibody to quantify nuclear and cytoplasmic myocardin levels. Total number of VSMC was ascertained by automated counting of the nuclei in the aortic media layer on a haematoxylin staining. Cardiac hypertrophy was quantified on the cellular level using an anti-laminin (Novus Biologicals, nb300-144) staining of cardiac sections to measure myocardial cross-sectional area. Five images of each mouse were recorded for this measurement in different cross-sectional regions of the heart, and 20 cardiomyocytes were measured per image (final average of 100 measurements). Microscopic images were acquired with Universal Grap 6.1 software using an Olympus BX4 microscope (×10 objective [aortic tissue] or ×40 objective [cardiac tissue]) or Celena S fluorescence microscope (×4 objective, DAPI LED filter [Ex375/28, Em460/50] and RFP LED filter [Ex470/30, Em530/50]) and quantified using ImageJ software.

### CaV1.2 Splice-variant PCR

To investigate the molecular mechanisms of aberrant VGCC function in the aorta, alternative splicing of the Cacna1C gene transcript was investigated. For the investigation of VGCC splice variants, the most average *n* = 5 from each age-group was selected (based on systolic BP, aPWV, and ex vivo E_p_) for RNA isolation from thoracic aortic tissue. RNA isolation and reverse transcription were carried out using a NucleoSpin® RNA kit (Macherey-Nagel) and a SensiFAST™ cDNA Synthesis Kit (Bioline), respectively. The resulting cDNA was subjected to a PCR reaction with exon 9* and exon 33 specific primers by Li et al.^70^. The resulting PCR product was separated on a 4% agarose gel and bands were quantified using ImageJ software.

### Statistics and reproducibility

Data are expressed as mean ± SEM, with *n* representing the number of biological replicates. All analyses were performed using GraphPad Prism (version 8, GraphPad Software) and a significance level of 5% was set to identify statistically significant changes. All data were included in the analysis, unless the data were influenced by experimental error and was identified as outlier during statistical testing in Graphpad Prism software. Normality of data was verified using the Kolmogorov–Smirnov test, and parametric testing was used when indicated. This includes one-sample *t*-test, one-way ANOVA, two-way ANOVA, and three-way ANOVA as indicated in the figure legends. A Tukey multiple testing correction was employed for post hoc testing of the ANOVA tests. For the main ageing experiment (2–24 months), post hoc testing for the factor age was performed vs. the 4-month value unless otherwise stated, since this represents the first age included in the experiment which represents the mature adult stage^17,18^. All attempts at replication were successful.

### Reporting summary

Further information on research design is available in the Nature Research Reporting Summary linked to this article.

## Supplementary information

Supplementary Information

Reporting Summary

Peer Review File

## Data Availability

The datasets generated during and/or analysed during the current study are available from the corresponding author on reasonable request.
