# Supplementary material for: Progressive aortic stiffness in aging C57Bl/6 mice displays altered contractile behaviour and extracellular matrix changes
Source: Commun Biol. 2022 Jun 17;5:605. doi: 10.1038/s42003-022-03563-x (PMC9203497; doi:10.1038/s42003-022-03563-x)
Supplement: Supplementary file 1 — Supplementary Information [file 42003_2022_3563_MOESM1_ESM.pdf]

Progressive aortic stiffness in aging C57Bl/6 mice displays altered contractile behaviour and extracellular matrix changes

Sofie De Moudt, Jhana O. Hendrickx, Cédric Neutel, Dorien De Munck, Arthur Leloup, Guido R.Y. De Meyer, Wim Martinet, Paul Fransen

Laboratory of Physiopharmacology, University of Antwerp, Belgium

Supplementary file.

1. Supplementary Tables

Supplementary Table 1: General characteristics of aging C57Bl6 mice.

Age (month)	2.8 ± 0.0	4.0 ± 0.0	6.1 ± 0.0	9.4 ± 0.0	12.1 ± 0.0	23.9 ± 0.7	p
n (none)	14	11	25	10	20	8	
Body weight (g)	24.8 ± 0.4	26.7 ± 0.5	31.4 ± 0.4	32.0 ± 0.6	33.7 ± 0.3	35.6 ± 1.2	***
Food intake (g/24h)	4.0 ± 0.0	4.1 ± 0.3	4.9 ± 0.3	4.2 ± 0.0	5.2 ± 0.3	N.A.	ns
Water intake (mL/24h)	5.3 ± 0.3	6.8 ± 0.7	7.1 ± 1.3	7.8 ± 0.0	8.2 ± 0.9	N.A.	ns
Urinary output (mL/24h)	1.6 ± 0.1	1.1 ± 0.1	1.9 ± 0.1	1.9 ± 1.2	2.3 ± 0.1	N.A.	***
Blood glucose (mg/dL)	185 ± 9	158 ± 6	172 ± 5	148 ± 5	147 ± 5	N.A.	***

Data are displayed as mean ± SEM. Statistical analysis using ordinary one-way ANOVA, overall significance is shown in the final column. ns p>0.05, *** p<0.001.

Supplementary Table 2: Extended cardiac parameters of aging C57Bl6 mice.

Age group	2	4	6	9	12	24	p
Heart weigth (mg)	166 ± 8	172 ± 5	182 ± 5	160 ± 8	167 ± 5	179 ± 14	ns
Heart rate (bpm)	438 ± 21	481 ± 39	474 ± 18	443 ± 26	456 ± 26	374 ± 58	ns
Stroke volume (mL)	34 ± 3	36 ± 3	38 ± 2	44 ± 3	38 ± 2	48 ± 4	*
EF (%)	80 ± 2	76 ± 3	78 ± 1	70 ± 2	80 ± 2	75 ± 4	ns
FS (%)	48 ± 2	45 ± 3	45 ± 1	39 ± 2	48 ± 2	45 ± 4	ns
IVRT (ms)	16.1 ± 1.1	14.4 ± 1.4	17.7 ± 0.8	21.8 ± 1.7	19.6 ± 0.5	22.4 ± 1.7	***
Deceleration (ms)	18.5 ± 1.3	22.2 ± 2.4	18.1 ± 1.1	18.1 ± 1.9	15.6 ± 1.0	13.7 ± 1.6	*
E/A ratio	1.29 ± 0.03	1.39 ± 0.05	1.39 ± 0.05	1.36 ± 0.05	1.48 ± 0.06	1.43 ± 0.12	ns

Abbreviations: EF, ejection fraction; FS, fractional shortening; IVRT, isovolumic relaxation time. Data are displayed as mean ± SEM. Statistical analysis using ordinary one-way ANOVA, overall significance is shown in the final column. ns p>0.05, * p<0.05, *** p<0.001.

2. Supplementary figures

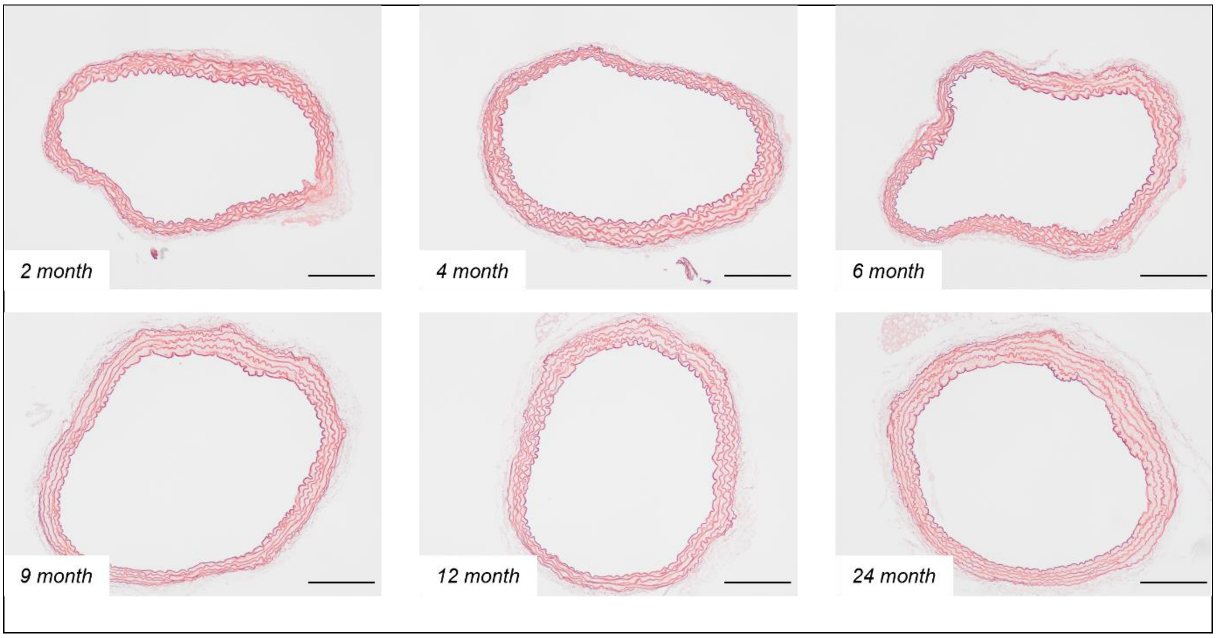

Supplementary Figure 1: Histological evaluation of aortic structure in aging C57Bl/6 mouse aortic tissue. Representative images of orcein-stained aortic tissue of 2, 4, 6, 9, 12, and 24 month old mice. Scale bar (bottom right) represents 200 µm.

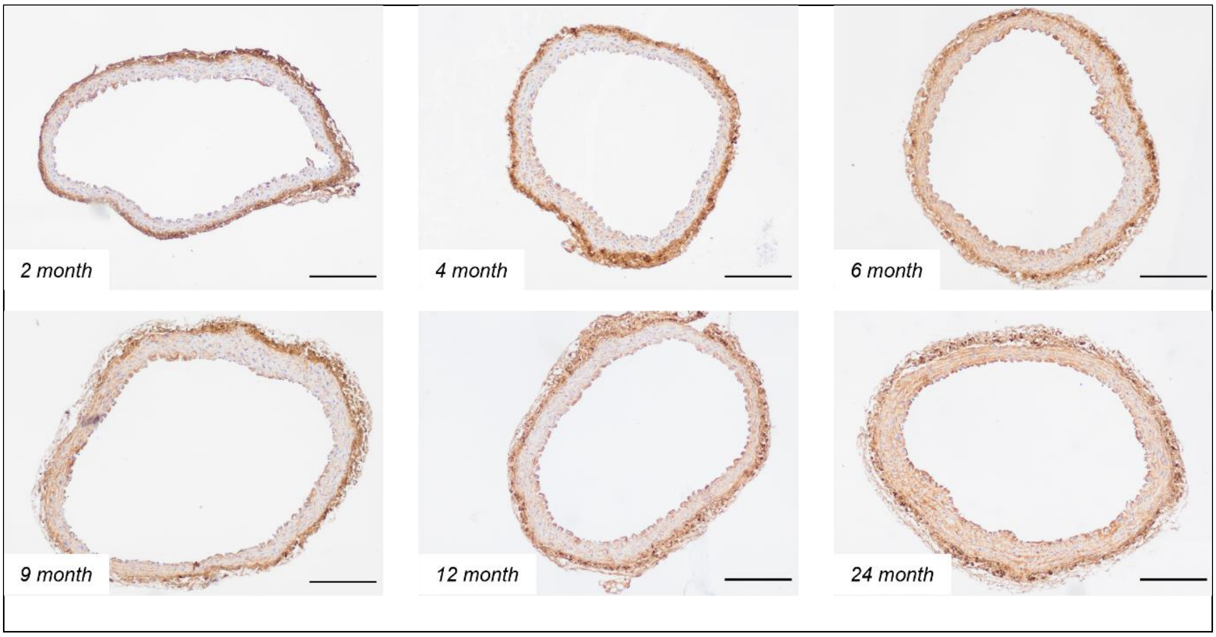

Supplementary Figure 2: Histological evaluation of collagen type I in aging C57Bl/6 mouse aortic tissue. Representative images of immunohistochemical staining for collagen type I fibres on aortic tissue of 2, 4, 6, 9, 12, and 24 month old mice. Scale bar (bottom right) represents 200 µm.

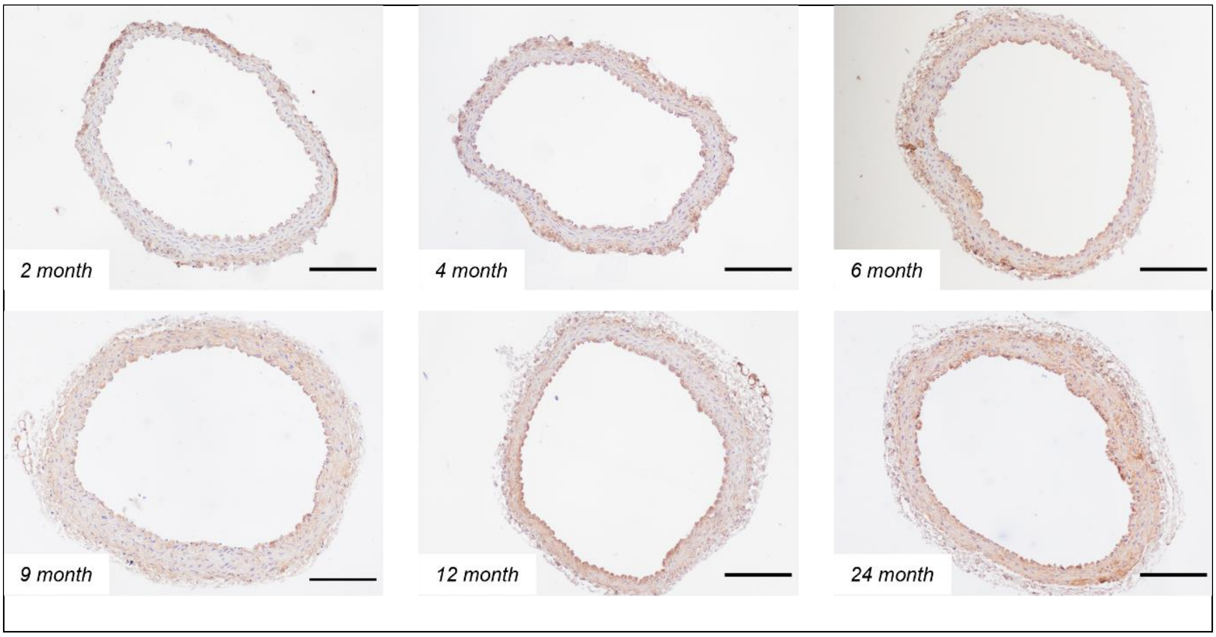

Supplementary Figure 3: Histological evaluation of collagen type III in aging C57Bl/6 mouse aortic tissue. Representative images of immunohistochemical staining for collagen type III fibres on aortic tissue of 2, 4, 6, 9, 12, and 24 month old mice. Scale bar (bottom right) represents 200 µm.

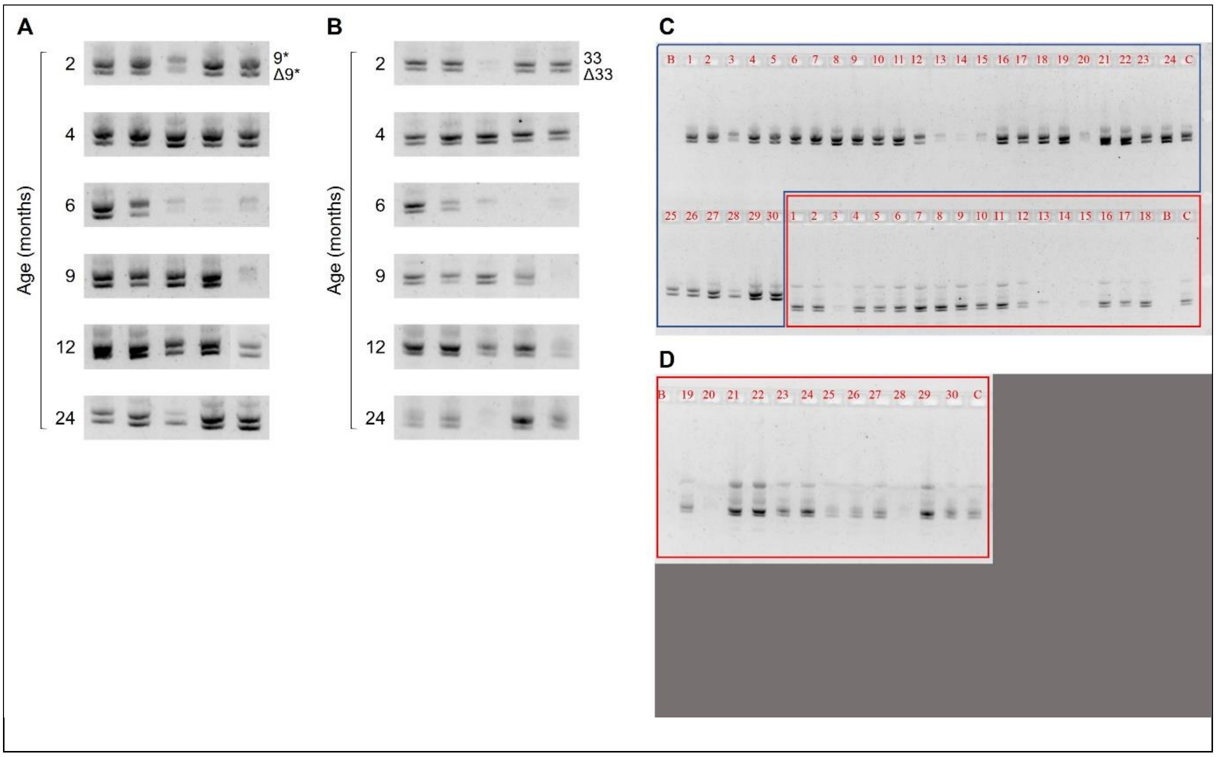

Supplementary Figure 4: CaV1.2 gel electrophoresis of aging C57Bl/6 mouse aortic tissue. Splice variation was assessed for CaV1.2 exon 9* and exon 33 by reverse-transcriptase PCR. Splice variants were separated using gel electrophoresis, as shown for exon 9* and Δ9* (A), and exon 33 and Δ33 (B). Full length gels for the exon 9* assay (blue square) and exon 33 assay (red square) are included in (C) and (D). Sample numbering: 1-5 (2 month), 6-10 (4 month), 11-15 (6 month), 16-20 (9 month), 21-25 (12 month), and 26-30 (24 month), B (blank), C (positive control).

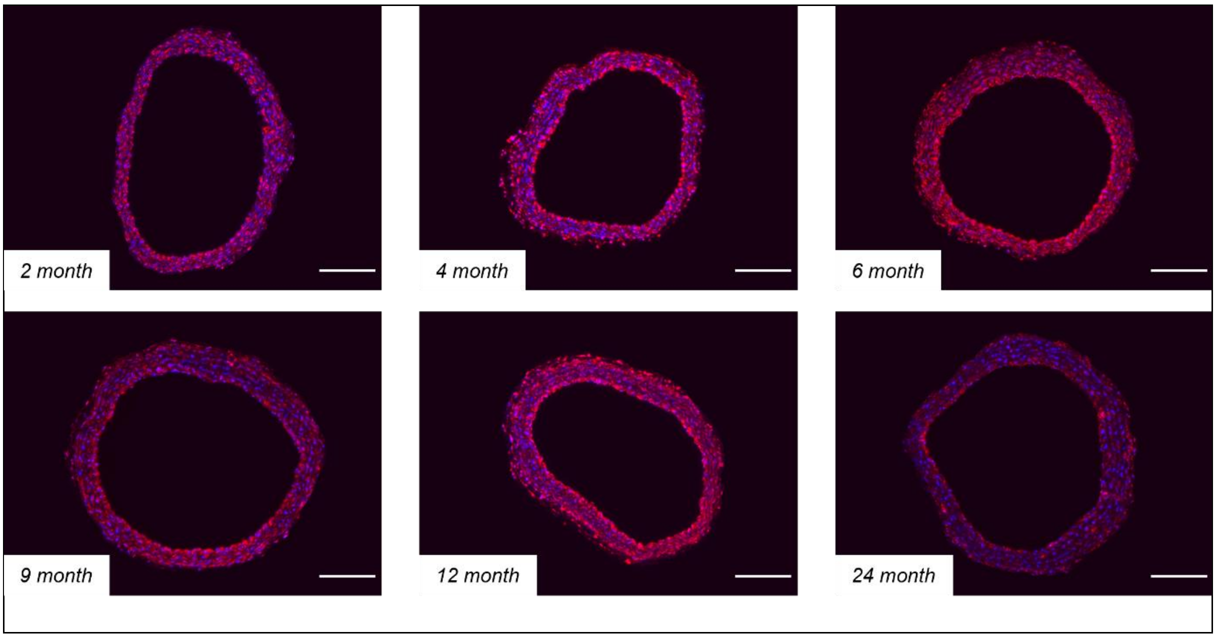

Supplementary Figure 5: Histological evaluation of nuclear myocardin fraction in aging C57Bl/6 mouse aortic tissue. Representative images of immunofluorescent staining for myocardin (red) and cell nuclei (blue) on aortic tissue of 2, 4, 6, 9, 12, and 24 month old mice. Scale bar (bottom right) represents 200 µm.

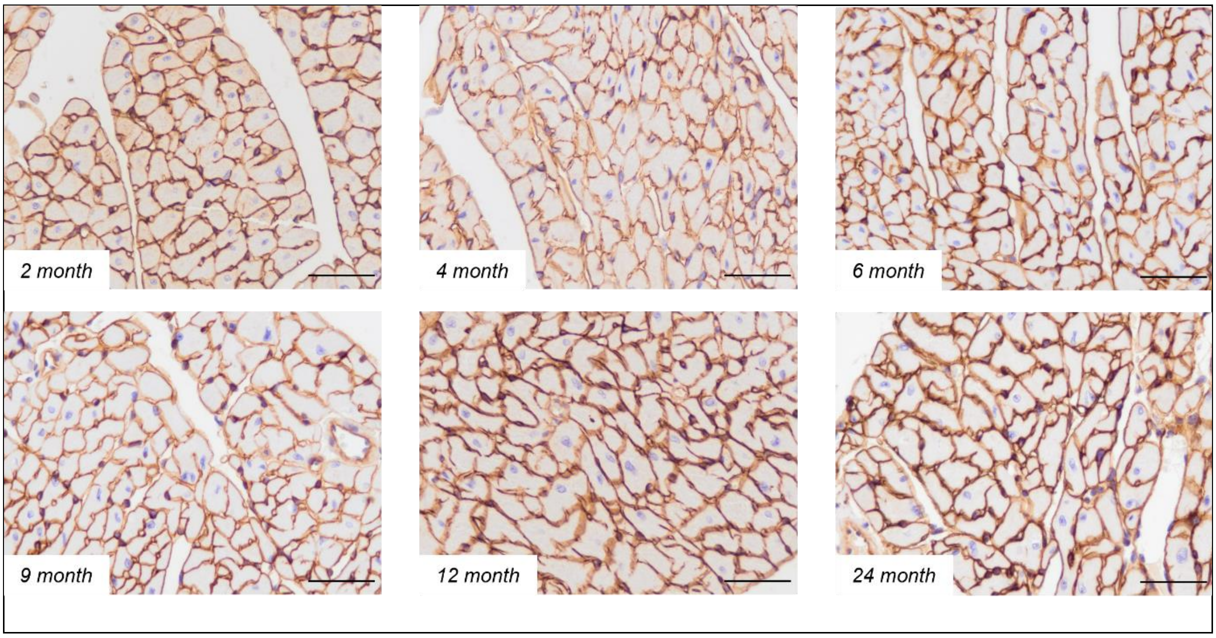

Supplementary Figure 6: Histological evaluation of cardiomyocyte cross-sectional area of aging C57Bl/6 mouse cardiac tissue. Representative images of laminin-stained cardiac tissue of 2, 4, 6, 9, 12 and 24 month old mice. Scale bar (bottom right) represents 50 µm.

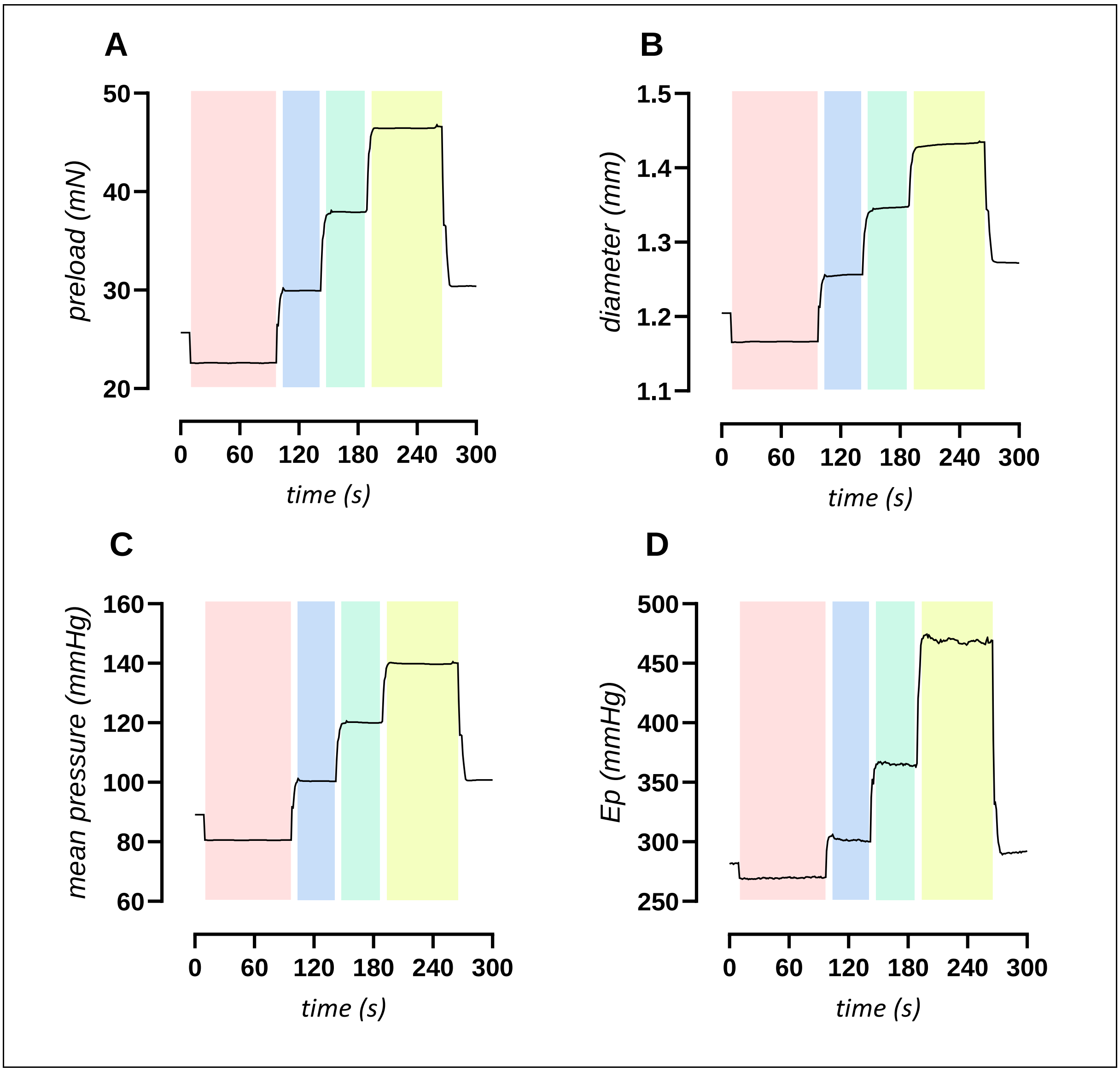

Supplementary Figure 7: Representative tracing of pressure-E_p curve recording. Measurement of force (A) and diameter (B) were used to calculate distending pressure (C) and Peterson modulus (E_p, D). Preload was adjusted to obtain 60-100 mmHg (red background), 80-120 mmHg (blue background), 100-140-mmHg (green background), and 120-160 mmHg (yellow background) pressure oscillations. On average, pressure-E_p curves were measured over a 5-minute period.
