# Supplementary material for: Progressive aortic stiffness in aging C57Bl/6 mice displays altered contractile behaviour and extracellular matrix changes
Source: Commun Biol. 2022 Jun 17;5:605. doi: 10.1038/s42003-022-03563-x (PMC9203497; doi:10.1038/s42003-022-03563-x)
Supplement: Supplementary file 2 — Reporting Summary [file 42003_2022_3563_MOESM2_ESM.pdf]

nature portfolio

Corresponding author(s): Sofie De Moudt

Last updated by author(s): Feb 14, 2022

Reporting Summary

Nature Portfolio wishes to improve the reproducibility of the work that we publish. This form provides structure for consistency and transparency in reporting. For further information on Nature Portfolio policies, see our Editorial Policies and the Editorial Policy Checklist.

Statistics

For all statistical analyses, confirm that the following items are present in the figure legend, table legend, main text, or Methods section.

n/a	Confirmed	
☐	☒	The exact sample size (n) for each experimental group/condition, given as a discrete number and unit of measurement
☐	☒	A statement on whether measurements were taken from distinct samples or whether the same sample was measured repeatedly
☐	☒	The statistical test(s) used AND whether they are one- or two-sided<br>*Only common tests should be described solely by name; describe more complex techniques in the Methods section.*
☐	☒	A description of all covariates tested
☐	☒	A description of any assumptions or corrections, such as tests of normality and adjustment for multiple comparisons
☐	☒	A full description of the statistical parameters including central tendency (e.g. means) or other basic estimates (e.g. regression coefficient) AND variation (e.g. standard deviation) or associated estimates of uncertainty (e.g. confidence intervals)
☒	☐	For null hypothesis testing, the test statistic (e.g. F, t, r) with confidence intervals, effect sizes, degrees of freedom and P value noted<br>*Give P values as exact values whenever suitable.*
☒	☐	For Bayesian analysis, information on the choice of priors and Markov chain Monte Carlo settings
☒	☐	For hierarchical and complex designs, identification of the appropriate level for tests and full reporting of outcomes
☒	☐	Estimates of effect sizes (e.g. Cohen's d, Pearson's r), indicating how they were calculated

Our web collection on statistics for biologists contains articles on many of the points above.

Software and code

Policy information about availability of computer code

Data collection	Labchart 8, Universal Grap 6.1
Data analysis	Graphpad Prism 8, ImageJ 1.35c

For manuscripts utilizing custom algorithms or software that are central to the research but not yet described in published literature, software must be made available to editors and reviewers. We strongly encourage code deposition in a community repository (e.g. GitHub). See the Nature Portfolio guidelines for submitting code & software for further information.

Data

Policy information about availability of data

All manuscripts must include a data availability statement. This statement should provide the following information, where applicable:

- Accession codes, unique identifiers, or web links for publicly available datasets
- A description of any restrictions on data availability
- For clinical datasets or third party data, please ensure that the statement adheres to our policy

The data that support the findings of this study are available from the corresponding author upon reasonable request.

Field-specific reporting

Please select the one below that is the best fit for your research. If you are not sure, read the appropriate sections before making your selection.

☒ Life sciences ☐ Behavioural & social sciences ☐ Ecological, evolutionary & environmental sciences

For a reference copy of the document with all sections, see nature.com/documents/nr-reporting-summary-flat.pdf

Life sciences study design

All studies must disclose on these points even when the disclosure is negative.

Sample size	To determine the sample size for the experiments, we used a power and sample size calculator (http://www.statisticalsolutions.net/pssZtest_calc.php) in combination with the expertise of the labs. We expected a 30% variation based on our expertise with the performed ex vivo measurements. Individual cohort sizes of 10 mice per time point were determined to provide a power of 0.89 for an unpaired t-test analysis. As the C57Bl/6 mice in the paper were used in a larger study (as controls for an experimental group which was not included in the manuscript), the experiment was repeated until a n=10 was reached in each group. Due to mortality, only n=8 was reached in the 24-month C57Bl/6 mice group. The analyzed sample size for C57Bl/6 mice was therefore between n=8 and n=25 for each individual age group. Sample size is indicated in the manuscript in the figure legends.
Data exclusions	All data were included in the analysis, unless the data was influenced by experimental error and was identified as outlier during statistical testing in Graphpad Prism software.
Replication	All attempts at replication were successful.
Randomization	Randomization was not relevant to the study as this was a spontaneous aging study. No study treatments etc. needed to be randomized
Blinding	Blinding was not relevant to the study as the main output parameters (e.g. blood pressure, isometric contractions) were not subject to observer bias.

Reporting for specific materials, systems and methods

We require information from authors about some types of materials, experimental systems and methods used in many studies. Here, indicate whether each material, system or method listed is relevant to your study. If you are not sure if a list item applies to your research, read the appropriate section before selecting a response.

Materials & experimental systems

n/a	Involved in the study	
☐	☒	Antibodies
☒	☐	Eukaryotic cell lines
☒	☐	Palaeontology and archaeology
☒	☐	Animals and other organisms
☒	☐	Human research participants
☒	☐	Clinical data
☒	☐	Dual use research of concern

Methods

n/a	Involved in the study	
☒	☐	ChIP-seq
☒	☐	Flow cytometry
☒	☐	MRI-based neuroimaging

Antibodies

Antibodies used	anti-collagen I (Abcam, ab21286), anti-collagen III (chemicon, MAB1343), and anti-collagen IV (DAKO, M0785), anti-myocardin (Sigma, sab4200539), anti-laminin (Novus Biologicals, nb300-144).
Validation	No information on the validation of the primary antibodies is available
