# Supplementary material for: Progressive aortic stiffness in aging C57Bl/6 mice displays altered contractile behaviour and extracellular matrix changes
Source: Commun Biol. 2022 Jun 17;5:605. doi: 10.1038/s42003-022-03563-x (PMC9203497; doi:10.1038/s42003-022-03563-x)
Supplement: Supplementary file 3 — Peer Review File [file 42003_2022_3563_MOESM3_ESM.pdf]

nature portfolio

Peer Review File

Open Access This file is licensed under a Creative Commons Attribution 4.0 International License, which permits use, sharing, adaptation, distribution and reproduction in any medium or format, as long as you give appropriate credit to the original author(s) and the source, provide a link to the Creative Commons license, and indicate if changes were made. In the cases where the authors are anonymous, such as is the case for the reports of anonymous peer reviewers, author attribution should be to 'Anonymous Referee' followed by a clear attribution to the source work. The images or other third party material in this file are included in the article's Creative Commons license, unless indicated otherwise in a credit line to the material. If material is not included in the article's Creative Commons license and your intended use is not permitted by statutory regulation or exceeds the permitted use, you will need to obtain permission directly from the copyright holder. To view a copy of this license, visit http://creativecommons.org/licenses/by/4.0/.

Reviewers' comments:

Reviewer #1 (Remarks to the Author):

This is an interesting paper that presents results on stiffening on C57Bl/6 mice and its relevance to tissue aging. This results have not been presented previously. The article is also well-written and-structured. The protocol is well defined and the experimental results are interesting. However, the results are not well presented.
In my opinion this article has several weaknesses that should be overcome in order for it to be published in COMMSBIO:
- The stiffness results are calculated and presented as the slope of the pressure increase vs diameter increase curve. This is not correct as the diameter increase at a given pressure depends on the wall thickness. As greater thickness, it is more difficult to increase the diameter at a given pressure, but this does not mean that the tissue is stiffer, but rather that the thicker the tube is, the more difficult it is to deform. To analyse whether the stiffness increases, it would be necessary to normalise the curve according to the vessel dimensions, i.e. to calculate the slope of the stress versus strain curve.
All the conclusions in the article are based on these results. It will be necessary to re-obtain the slopes and then compare the rest of the results with the stiffness to see if the conclusions hold or are modified.

Reviewer #2 (Remarks to the Author):

In this manuscript, the authors conducted a comprehensive study on the impact of aging on the vascular biomechanical properties using in vivo arterial stiffness measurement and ex vivo biomechanical characterization test. The major finding was age-dependent arterial stiffening in C57Bl/6 mice preceded cardiac hypertrophy and hypertension. In addition, progressive arterial stiffening was due to changes in extracellular matrix and vascular smooth muscle cell signaling rather than endothelial dysfunction. This study provided valuable insight into how aging affect cardiovascular health and addressed the potential of using aortic stiffness as an early marker in the diagnosis and prevention of cardiovascular disease. However, there are some concerns and issues about the study as listed below:

Method section
1.What is the rationale for only using male mice in this study? Did the authors conduct any pilot study to observe sex difference in the aortic stiffening?

2.What is the rationale for using abdominal PWV for in vivo measurement while ex vivo biomechanical test was conducted on Descending thoracic aorta? Is it possible to obtain PWV of DTAo?

3.In reactivity studies in the method section, what is the preload applied to aortic segments of all age groups? Is it universal 20mN as described in the beginning, or age-dependent data listed in the following section? When conducting PE concentration-response stimulation, which concentrations were selected in the range of 3 nM to 10 μM? Is there any concentration-response curve and followed up analysis?

4.In the method section of measurement of ex vivo aortic stiffness, some info is missing: 1).are these preloads applied on aortic segments age-dependent? What is individual preload applied to mimic the different pressure range? 2).When obtaining each Ep over the increasing pressure range (60-100 to 120-160), was the test conducted continuously on the same aortic segment with increasing pressure? What are time intervals between pressure change?

5.Histology: It was stated in the method section of CaV1.2 Splice-variant PCR, the most average n=5 from each age-group was selected. Also n=5 for both histological and IHC study, did the selection follow the same manner?

Result section

1. In Figure 1B, *** p<0.001 at 6-month vs 2mo for Ep at isobaric conditions of 80-120, while in Figure 2A, the * p<0.05 at 6 mo vs 2 mo for same condition. Why p value changes significantly when comparing same data?

2. When acquiring the data in Figure 3A, after adding 2 μM PE into ROTSAC bath, isometric force (contraction force) was recorded right away or is there any time lag? Is it same for force measurement after adding L-NAME? Same issues for data acquisition in Figure 4 when using potassium and force change in Figure 5 when adding acetylcholine?

3. When acquiring the data in Figure 3D, what was the concentration of PE? How to calculate the isometric force change %? Is it relative to the state with & without agonist before adding diltiazem?

4. Histology: In either method section or figure legend, the magnification of microscope for imaging is missing. The scale bar in orcein-stained slides in supplementary Figure 1 is not clear. Which fluorescent filter (emission/excitation info) was used to image the myocardin stained section in supplementary Figure 2 ?

Minor editorial issues:
1.A few typos (Line 11: "Our" spelled as Out, Line 79: p=0.021, Line 103: -7.1±0.1 log(M), Line 275: "recording" as "recoding", Line 348: "of" as "off",
2.Wrong labeling in Figure 3 legend: Statistical analysis using two-way ANOVA in (A,D, F) NOT (B,D,F). Also, data are listed as mean±SEM in (A,D-F, n>8) NOT (B,D-F, n>8).
Wrong labeling in Figure 4 legend: Data are listed as mean±SEM (A,E, n>5) NOT (A,F, n>5).
Wrong description about F in Figure 4 legend.

Reviewer #3 (Remarks to the Author):

The manuscript submitted by De Moudt et al. characterized and explored the mechanisms of age-related aortic stiffening in C57BL/6 mice. The study used a longitudinal study design in mice to assess aortic stiffness using the gold standard measurement, aortic pulse wave velocity (PWV), and assessed elastic modulus, cardiac function, endothelial function, extracellular matrix remodeling, splice variation and isometric reactivity to determine the mechanisms by which arterial stiffness occurs throughout the lifespan. The investigators should be commended for their heroic efforts in accomplishing this comprehensive study. However, in its current form, the narrative throughout this manuscript does not clearly reflect the data, which markedly reduced enthusiasm. Despite the reduced enthusiasm, I believe that adjustments to the overall narrative, rationale and detail of particular experimental approaches and statistical analyses would significantly improve the impact of the manuscript.

Major
• Narrative: The introduction is presented in the style of a general review of the literature. As such, the structure of the introduction does not accurately set up the study design and results narrative.

• Narrative: There needs to be a reworking of the statistics/narrative to fit the data. Currently most statistics are comparing the 2 mo timepoint to other timepoints. It may be advantageous to leverage the 6 mo timepoint since that is considered a fully developed "young adult" mouse and is more consistent with the literature in the cardiovascular aging field. The period between 2 and 6 months

shows how CV function is changed throughout the maturation/development process in mice, but not aging per se.

• Statistical analyses: All analyses were conducted relative to the 2 month time-point. This approach does not accurately represent the aging effect on the outcomes assessed. Rather, analyses should be conducted across all time-points. Furthermore, the most accurate representation of the aging effect would be to compare the 6 mo time-point (time-point representing the earliest stage of physical maturation [i.e., young adulthood]) vs. the 24 month time-point [i.e., older adulthood]).

• Narrative: Numerous techniques and abbreviations, such as VGCC and Cacna1C, are not well explained in the introduction or prior to use in the results section.

• Rationale: Clear rationale should be provided regarding the selection of mouse ages. Middle-aged for this mouse strain is ~18 months and the median lifespan for this strain is ~27 months – why weren't these ages used? Furthermore, it is not clear in the literature as to how vascular function changes through physical maturation (2-6 months in this mouse strain), so this is a novel component of the study; however, authors do not highlight this fact.

• Rationale: There is no justification for the varying sample sizes in different figures or why there are different mouse number at different timepoints within a given figure.

• Rationale: In the results section it would be helpful to explain why certain pressure ranges were selected for Peterson's modulus. How do those pressure ranges relate to mouse and human physiology?

• Rationale: The rationale for using L-NAME in particular experiments is not well justified. If authors hypothesize that NO mediates the responses they are measuring, that should be clearly stated throughout.

• Methods/Rationale: Better justification and rationale is needed to explain why certain methods such as CaV1.2 splice variant PCR and isometric reactivity were used to study aortic stiffness.

• Methods: Inclusion of tracking points in the PWV graphs by using lines in between the same mouse to ensure similar trends with age would be helpful to the audience.

• Methods: Only one aortic cross-sectional image per group is included to represent the histology data. Rather one section per group should be provided for each sub analysis. This will enhance rigor and reproducibility.

• Data interpretation: The PWV measures are a bit concerning, given that these are genetically identical mice who are not on intervention there is a very large spread (nearly 350 cm/sec at some time points). Please expand on this finding in the discussion/limitations section….Showing individual mouse trends in PWV (as mentioned above) could limit this concern.

• Data interpretation: Type-1 collagen is the primary isoform of collagen in arteries and does not show a clear "aging" pattern, while Type-3 collagen does. Throughout the discussion, authors describe these data collectively, when there were clearly differring age-related changes between the two isoforms.

• Narrative/Data interpretation: Previous lifelong vascular function studies have been conducted in this same mouse strain (PMID: 33103241). The results of this previous lifelong study should be considered in the context of the present study. In particular, this previous publication highlights the role of vascular mitochondrial oxidative stress and inflammation and reduced nitric oxide bioavailability as mediators of vascular aging (i.e., mechanisms underlying vascular aging). Furthermore, this publication showed a clear phenotype for age-related endothelial dysfuncton(where the current

manuscript did not), albeit in a pressurized carotid artery model (which is suggested to be more translational than the aortic ring model for assessing endothelium-dependent dilation).

• Data interpretation: The fact that changes in PWV precede any changes in blood pressure, is certainly interesting (which was also shown in the lifelong study highlighted above); however, PWV is considered the "gold standard" technique for assessing large elastic artery stiffness, while tail-cuff blood pressure is not a "gold-standard" technique. Further, there is not enough data to claim that endothelial function is not relevant to arterial dysfunction. This statement can be made when specifically referring to the mice in this study but it not all-encompassing to other mouse strains or even other cohorts.

Minor
• Methods: The authors do not clearly identify the mouse strain, C57BL/6 (J? or N?), where the strain was obtained, and the specific mouse chow used in the study. Details such as these are essential for enhancing rigor and reproducibility.
• Narrative: Line 37: Define "spontaneously aging."
• Grammar: Line 45, "aortic" should not be capitalized.
• Narrative: Line 45-46, aPWV is not a marker but the gold-standard method to assess aortic stiffness.
• Data interpretation: It would be informative for the reader if authors would highlight the individual data. The manuscript states that PWV increased by 250%, which doesn't appear to be the case for all mice.
• Data interpretation: Line 49: "age-related stiffening" should not be assessed in 2 vs. 6 mo old mice. This time-frame represents physical maturation, not aging.
• Methods: Line 64-65,69,76,88, compound concentrations not necessary; include in the materials and methods section
• Narrative: Line 66,83, NOS should be spelled out before and defined in the previous sentence where it was introduced
• Rationale/ Data interpretation: Line 70, why are mice starting at 6 mo considered older (6 months is equivalent to ~30 in human years)?
• Rationale: Line 80 & Figure 4A, why were these age groups chosen?
• References: Lines 112-119, the references are not properly inserted.
• Narrative: Line 227, "BP" should be spelled out or defined previously.
• Narrative: Line 242: Define "carotid-femoral PWV."
• Narrative: Line 247: "…affirming the use of aortic stiffness as an early marker of CVD monitoring" is a very broad statement. This can only be described in the context of this mouse model.
• Figures/Methods: Figure 3E statistical symbols do not directly relate to legend below.
• Figures/Rationale: Figure 5B lack of rationale for comparing 8 to 24 mos.

REVIEWER #1

This is an interesting paper that presents results on stiffening on C57Bl/6 mice and its relevance to tissue aging. This results have not been presented previously. The article is also well-written and-structured. The protocol is well defined and the experimental results are interesting. However, the results are not well presented.

In my opinion this article has several weaknesses that should be overcome in order for it to be published in COMMSBIO:

1) The stiffness results are calculated and presented as the slope of the pressure increase vs diameter increase curve. This is not correct as the diameter increase at a given pressure depends on the wall thickness. As greater thickness, it is more difficult to increase the diameter at a given pressure, but this does not mean that the tissue is stiffer, but rather that the thicker the tube is, the more difficult it is to deform. To analyse whether the stiffness increases, it would be necessary to normalise the curve according to the vessel dimensions, *i.e.* to calculate the slope of the stress versus strain curve.

   All the conclusions in the article are based on these results. It will be necessary to re-obtain the slopes and then compare the rest of the results with the stiffness to see if the conclusions hold or are modified.

Please find the FIGURES FOR REVIEWER, REVISED MANUSCRIPT FIGURES, and REVISED MANUSCRIPT SUPPLEMENTARY FIGURES at the end of this document. All changes to the revised manuscript text are indicated in this document in **bold/underlined/grey highlight**.

The reviewer is correct in stating that the manuscript does not take into account wall thickness when calculating aortic stiffness, since we use the Peterson modulus (E_p) to assess aortic stiffness, which is calculated as $E_p = D_0(\Delta P/\Delta D)$, with D, diameter; P, pressure; and D_0, start diameter. By definition, this equation therefore does not take into account wall thickness. As a result, E_p values are indeed different from true material stiffness parameters such as the Youngs modulus, where stiffness is interpreted as a 'material' or 'tissue' property, whereas E_p represents the structural stiffness of the entire aortic segment, including its wall thickness.

As the reviewer correctly states: "at greater thickness, it is more difficult to increase the diameter at a given pressure." We consider that this would be true for the *in vivo* aorta as well; that when wall thickness increases, the aorta will be more difficult to distend, resulting in the known adverse hemodynamic alterations related to aortic stiffening. The Peterson elastic modulus can be interpreted as the pressure change that is required to increase aortic diameter by 100%. Although it is therefore not a measure of material stiffness, we believe this parameter holds a greater relevance for the *in vivo* hemodynamic situation. Clinical findings confirm that local measures such as E_p correlate well with global PWV *[1]*. In our opinion, the added value of the *ex vivo* measurements is to correct for *in vivo* confounding factors (*e.g.*, blood pressure, heart rate, circulating factors) and to allow us to assess active mediators of aortic stiffness using vasoactive agents. In the present manuscript, we describe E_p and aortic pulse wave velocity (aPWV) for C57Bl/6 mice aged 2-24 months of age. Figure 1 for Reviewer shows the correlation of *ex vivo* E_p and *in vivo* aPWV, which demonstrates that the Peterson modulus was a representative method to assess aortic stiffness *ex vivo*.

Furthermore, it was previously published that a 60% increase in wall thickness only increased Peterson modulus by 16% *[2]*. Since we report a 34% increase in aortic wall thickness and a 27% increase in E_p in 24-month old mice (versus 2-month old mice), the authors do not believe that the observed differences in aortic stiffness with age solely reflect aortic wall thickening, and we do expect that material stiffness would be increased as well. However, the authors did not make any reference to this in the original manuscript, only describing changes in arterial stiffness (not tissue stiffness) since we realize that this is not a statement we could support based on the data. Although it would therefore

be interesting to assess tissue stiffness by taking into account aortic wall stiffness, our equipment is unable to measure aortic wall thickness dynamically during the measurements and it is therefore not feasible for us to include the requested calculations.

To conclude, we agree with the Reviewer that it would be interesting to assess tissue stiffness in aging C57Bl/6 mice, but are unfortunately unable to do so using the equipment we have at hand. However, we want to stress that we do believe that the presented data using Peterson modulus are representative for *in vivo* aortic stiffening (possibly even more so than when using a parameter which corrects for wall thickness *[1,3]*), and that the manuscript therefore demonstrates important and novel findings regarding the mechanisms of age-related aortic stiffening in C57Bl/6 mice. To accommodate the Reviewer's comment, the authors updated the manuscript discussion to include this as a study limitation (Lines 292-299): "**It needs to be emphasized that the presented data in this manuscript only includes assessments of structural aortic stiffness of the entire arterial segment, rather than assessments of material stiffness of aortic tissue, since no correction was made for aortic wall thickness during *ex vivo* biomechanical testing. Rachev et al *[2]* previously reported that a 60% increase in wall thickness only increased E_p by 16%. Since the present manuscript reports a 34% increase in aortic wall thickness and a 27% increase in E_p in 24-month old mice (versus 2-month old mice), the presented data suggest that the observed differences in *ex vivo* aortic stiffness were not solely the result of aortic wall thickening and that age-related aortic tissue stiffening may also occur in spontaneously aging C57Bl/6 mice. This could represent an interesting topic for future research.**"

[1] Nagai, Y. et al. Carotid arterial stiffness as a surrogate for aortic stiffness: relationship between carotid artery pressure-strain elastic modulus and aortic pulse wave velocity. Ultrasound Med Biol 25, 181-188, doi:10.1016/s0301-5629(98)00146-x (1999).

[2] Rachev, A., Stergiopulos, N. & Meister, J. J. A model for geometric and mechanical adaptation of arteries to sustained hypertension. J Biomech Eng 120, 9-17, doi:10.1115/1.2834313 (1998).

[3] Spronck, B. & Humphrey, J. Arterial Stiffness: Different Metrics, Different Meanings. J Biomech Eng, doi:10.1115/1.4043486 (2019).

REVIEWER #2

In this manuscript, the authors conducted a comprehensive study on the impact of aging on the vascular biomechanical properties using *in vivo* arterial stiffness measurement and *ex vivo* biomechanical characterization test. The major finding was age-dependent arterial stiffening in C57Bl/6 mice preceded cardiac hypertrophy and hypertension. In addition, progressive arterial stiffening was due to changes in extracellular matrix and vascular smooth muscle cell signaling rather than endothelial dysfunction. This study provided valuable insight into how aging affect cardiovascular health and addressed the potential of using aortic stiffness as an early marker in the diagnosis and prevention of cardiovascular disease. However, there are some concerns and issues about the study as listed below:

Firstly, the authors wish to thank the reviewer for the thorough review of this manuscript. The authors believe he/she raised some interesting comments which enhanced the value of the revised work. Please find the FIGURES FOR REVIEWER, REVISED MANUSCRIPT FIGURES, and REVISED MANUSCRIPT SUPPLEMENTARY FIGURES at the end of this document. All changes to the revised manuscript text are indicated in this document in **bold/underlined/grey highlight**.

Method section

1) What is the rationale for only using male mice in this study? Did the authors conduct any pilot study to observe sex difference in the aortic stiffening?

The authors acknowledge that important sex-differences are expected when studying cardiovascular disease. Considering that gender would likely add an important layer of variation, and data in male and female animals would therefore need to be stratified according to gender, the authors believed that this would add too much complexity to a study that already contained a total of 101 mice and +6 age groups. Therefore, we opted to focus on the effects in male mice only. To accommodate this comment, the authors updated the study conclusion to state that it applies to male animals only, as not to generalize the findings to C57Bl/6 mice of both genders. The conclusion now states (Line 301): "Progressive arterial stiffening was observed in ageing **male** C57Bl/6 mice, …"

2) What is the rationale for using abdominal PWV for *in vivo* measurement while *ex vivo* biomechanical test was conducted on Descending thoracic aorta? Is it possible to obtain PWV of DTAo?

Why we choose the descending thoracic aorta for *ex vivo* measurement: The descending thoracic aorta is the model example of an elastic blood vessel because of its close proximity to the heart. Furthermore, it is an anatomically interesting part of the aorta for *ex vivo* testing because of its long, straight nature. The abdominal aorta, on the other hand, has several large branching arteries, such as the mesenteric and renal arteries, and displays important differences in aortic stiffness and physiology depending on its suprarenal or infrarenal location (see Figure 2a,b for Reviewer), making this less suitable for *ex vivo* testing.

Why we choose the abdominal aorta for *in vivo* measurement: *In vivo* pulse wave velocity is measured by B-mode images of aortic diameter and pulsed wave Doppler analysis of blood flow velocity. These measurements are hard to obtain from the thoracic aorta due to the interfering presence of heart, lungs, and ribs, and strong interference of movement during in-and exhalation, whereas the abdominal aorta is easily imaged as demonstrated in Figure 2c for reviewer. Pulse wave velocity is measured at the site indicated by 'abdominal aorta' on this image, just proximal of the curvature leading to the renal vessels. Ultrasound assessment of aPWV is considered the gold standard for PWV measurement in mice.

Why measurements of descending aorta and suprarenal abdominal aorta are closely relatable: We have previously performed measurements of *ex vivo* aortic diameter and stiffness at different sites of the aorta, *i.e.*, the ascending thoracic aorta, descending thoracic aorta, and suprarenal abdominal

aorta (see Figure 2a,b for Reviewer). These experiments showed significantly decreasing aortic diameter and significantly increasing aortic stiffness from the proximal to distal aorta. However, no significant differences in either diameter or stiffness were observed between the descending thoracic aorta and suprarenal abdominal aorta. A marked difference in aortic stiffness was mainly observed for the infrarenal abdominal aorta. Therefore, the authors concluded that the two selected sites chosen for *in vivo* (suprarenal abdominal aorta) and *ex vivo* (descending thoracic aorta) measurements would provide closely inter-relatable information. Indeed, we have shown a clear correlation for *in vivo* aPWV and *ex vivo* E_p measurements of the male C57Bl/6 mice used in the present study (Figure 1 for Reviewer).

3) In reactivity studies in the method section, what is the preload applied to aortic segments of all age groups? Is it universal 20mN as described in the beginning, or age-dependent data listed in the following section? When conducting PE concentration-response stimulation, which concentrations were selected in the range of 3 nM to 10 �M? Is there any concentration-response curve and followed up analysis?

Regarding the preload for isometric reactivity testing: During the main aging study (mice aged 2-24 months of age), isometric reactivity was measured using a fixed preload of 20 mN. We have previously shown that for healthy adult mice, this preload represents to a mean blood pressure of 100 mmHg *[1]*, which is why it was applied throughout the experiment as a physiological relevant preload. On the other hand, the follow-up experiment in 5 and 26 month old mice was performed in ROTSAC organ chambers which allows for calibration of the individual aortic segment (contrary to classic isometric organ baths). Therefore, we could apply the correct preload corresponding to 100 mmHg for each individual aortic ring. In these mice, we observed that a higher preload was required in old versus young mice (26.7±0.8 mN for 5-month old mice [n=6] versus 30.0±0.5 mN for 26-month old mice [n=4]) as was stated in the methods section of the original manuscript. We clarified these different uses of isometric preload in the updated manuscript (Lines 356, 371-374): "**In the main aging experiment (2-24 months of age), a fixed** preload of 20 mN was applied to approximate normal physiological stretch at a mean blood pressure of 100 mmHg, … **In a follow-up experiment (separate group of 5 and 26 months old mice),** both isometric and isobaric measurements were performed in a Rodent Oscillatory Tension Set-up for Arterial Compliance (ROTSAC), allowing for calibration of aortic segments and resulting application of a preload corresponding to a calculated pressure of 100 mmHg **for each individual aortic ring. This** preload was significantly increased from 26.7±0.8 (n=6) to 30.0±0.5 mN (n=4) for 5 and 26 months old mice**, respectively**. …"

To verify the correctness of the applied preloads in the main aging study, we performed a post-hoc analysis of the ROTSAC organ chamber measurements to ascertain the 'ideal' preload to obtain a calculated distending pressure of 100 mmHg. The results are shown in Figure 3 for Reviewer, demonstrating that, indeed, preload should have ideally been adjusted for age. However, preload was only significantly increased in the 24-month old mice (26.83±0.50 mN, versus 23.44±0.24 mN in the 4-month age group). The ~3 mN increase in 'ideal' preload with age, however, is not of the magnitude expected to affect isometric reactivity according to our previously published work *[1]*. Therefore, the authors believe that the difference in optimal preload due to age was not expected to greatly affect the data presented in this study.

Regarding the PE concentration response curves: PE concentration-response curves were performed in the absence and presence of voltage-gated calcium channel (VGCSC) agonist BAY-K8644 (30 nM), as stated in the method section of the original manuscript. The cumulative final doses included: $3*10^{-9}$ M, 10^{-8} M, $3*10^{-8}$ M, 10^{-7} M, $3*10^{-7}$ M, 10^{-6} M, $3*10^{-6}$ M, and 10^{-5} M (in a log scale, this represents an increase per 0.5 log(M) dose). From the dose-response experiments, the maximal effect and EC_{50} values were calculated using a non-linear 4-parameter equation as stated in the methods

section of the manuscript. Due to the complexity of the graphical representation of dose response data for 6 age groups, only the maximal value of the PE concentration response curve was shown in the manuscript (see Figure 3d of the manuscript). To clarify, both the original dose-response curves and the calculated maximal effect and EC_{50} values were included in Figure 4 for Reviewer.

[1] DE MOUDT, S., LELOUP, A., VAN HOVE, C., DE MEYER, G. & FRANSEN, P. 2017. Isometric Stretch Alters Vascular Reactivity of Mouse Aortic Segments. Front Physiol, 8, 157.

4) In the method section of measurement of *ex vivo* aortic stiffness, some info is missing: 1).are these preloads applied on aortic segments age-dependent? What is individual preload applied to mimic the different pressure range? 2).When obtaining each Ep over the increasing pressure range (60-100 to 120-160), was the test conducted continuously on the same aortic segment with increasing pressure? What are time intervals between pressure change?

For the *ex vivo* aortic stiffness measurements in the ROTSAC organ chambers, the applied preloads are determined by calibration of each individual aortic ring, so indeed, these are pressure and age-dependent (see previously, Figure 2 for Reviewer). For the description of the method and calibrations, the authors refer to the original publication of the ROTSAC method by Leloup et al *[1]*. The calibration allows us to dynamically adjust the preload to obtain the correct calculated pressures for each individual aortic ring. For the pressure range, the test was conducted continuously on the same aortic segment using increasing preloads. To clarify the timing, a supplementary figure (identical to Figure 5 for Reviewer) was prepared which shows a tracing of the measurement. The method section was updated in the revised manuscript as follows (Line 389-392): "Aortic stiffness was always assessed in isobaric conditions, and measured at oscillating pressures of 60-100, 80-120, 100-140 and 120-160 mmHg. **Corresponding preloads were applied consecutively on each aortic segment at increasing distending pressure as shown in the representative tracing in Supplementary Figure 7**."

[1] LELOUP, A. J., VAN HOVE, C. E., KURDI, A., DE MOUDT, S., MARTINET, W., DE MEYER, G. R., SCHRIJVERS, D. M., DE KEULENAER, G. W. & FRANSEN, P. 2016. A novel set-up for the ex vivo analysis of mechanical properties of mouse aortic segments stretched at physiological pressure and frequency. J Physiol, 594, 6105-6115.

5) Histology: It was stated in the method section of CaV1.2 Splice-variant PCR, the most average n=5 from each age-group was selected. Also n=5 for both histological and IHC study, did the selection follow the same manner?

Indeed, these were samples taken from the same mice. To clarify: the mean values for systolic blood pressure (SBP), aortic pulse wave velocity (aPWV), and *ex vivo* Peterson modulus (E_p) were calculated. For each mouse, the deviation of its SBP, aPWV, and E_p values from these means was calculated. The 5 mice with the lowest sum of deviations from mean SBP, aPWV, and E_p were selected for histological and molecular experiments.

<u>*Result section*</u>

6) In Figure 1B, *** p<0.001 at 6-month vs 2mo for Ep at isobaric conditions of 80-120, while in Figure 2A, the * p<0.05 at 6 mo vs 2 mo for same condition. Why p value changes significantly when comparing same data?

Although these data at 80-120 mmHg are indeed identical for Figure 1b and Figure 2a, as the reviewer correctly states, different statistical methods were applied to the data, as was mentioned in their respective figure legends. For Figure 1b, statistical analysis was done using one-way ANOVA (factor: age) with post-hoc testing versus 2-month old mice (updated to 4 month old mice in the revised manuscript, as requested by Reviewer 3). For Figure 2a, statistical analysis was done using two-way ANOVA (factors: age, pressure), to include the effect of distending pressure in the statistical testing.

This resulted in a higher number of post-hoc tests, and - as follows - a more stringent multiple testing correction. Therefore, the significance level was lower in Figure 2a compared to Figure 1b.

7) When acquiring the data in Figure 3A, after adding 2 �M PE into ROTSAC bath, isometric force (contraction force) was recorded right away or is there any time lag? Is it same for force measurement after adding L-NAME? Same issues for data acquisition in Figure 4 when using potassium and force change in Figure 5 when adding acetylcholine?

For all vasoreactivity studies, measurements were always performed when a stable condition was reached. For 2 µM PE contractions, it usually takes 10 minutes to reach a stable contraction. For 300 µM L-NAME (in the presence of 2 µM PE), this usually takes 15 minutes. Potassium concentrations also take up to 15 minutes to stabilize, whereas acetylcholine usually takes less than 2 minutes per concentration. This was clarified in the method sections (Line 378): "**All measurements were performed after steady-state conditions were reached.**"

8) When acquiring the data in Figure 3D, what was the concentration of PE? How to calculate the isometric force change %? Is it relative to the state with & without agonist before adding diltiazem?

The data in Figure 3d were obtained from the maximal effect calculation of the dose-response curves using a non-linear regression. This was clarified in the figure legend in the revised manuscript: "… **Maximal** PE contractions**, as obtained from non-linear regression of the PE concentration response curves,** in the absence (circles) and presence (squares) of 30 nM BAY-K8644 (D), …"

Indeed, as the reviewer correctly states, the effect of diltiazem on isometric force was calculated as the percentage contraction inhibition compared to the 10 µM PE contraction. This is illustrated in Figure 6 for Reviewer, which shows a representative tracing of the measurement.

9) Histology: In either method section or figure legend, the magnification of microscope for imaging is missing. The scale bar in orcein-stained slides in supplementary Figure 1 is not clear. Which fluorescent filter (emission/excitation info) was used to image the myocardin stained section in supplementary Figure 2?

The scale bar for the orcein staining was updated in the revised manuscript, and the information on magnification and fluorescent filters was added to the methods section of the manuscript (Lines 407-410): "Microscopic images were acquired with Universal Grap 6.1 software using an Olympus BX4 microscope **(10x objective [aortic tissue] or 40x objective [cardiac tissue])** **or Celena S fluorescence microscope (4x objective, DAPI LED filter [Ex375/28, Em460/50] and RFP LED filter [Ex470/30, Em530/50])** and quantified using ImageJ software."

Minor editorial issues:

10) A few typos (Line 11: "Our" spelled as Out, Line 79: p=0.021, Line 103: -7.1�0.1 log(M), Line 275: "recording" as "recoding", Line 348: "of" as "off",

11) Wrong labeling in Figure 3 legend: Statistical analysis using two-way ANOVA in (A,D, F) NOT (B,D,F). Also, data are listed as mean�SEM in (A,D-F, n>8) NOT (B,D-F, n>8).

12) Wrong labeling in Figure 4 legend: Data are listed as mean�SEM (A,E, n>5) NOT (A,F, n>5).

13) Wrong description about F in Figure 4 legend.

Minor editorial issues (Questions 10-13) were corrected in the revised manuscript.

REVIEWER #3

The manuscript submitted by De Moudt et al. characterized and explored the mechanisms of age-related aortic stiffening in C57BL/6 mice. The study used a longitudinal study design in mice to assess aortic stiffness using the gold standard measurement, aortic pulse wave velocity (PWV), and assessed elastic modulus, cardiac function, endothelial function, extracellular matrix remodeling, splice variation and isometric reactivity to determine the mechanisms by which arterial stiffness occurs throughout the lifespan. The investigators should be commended for their heroic efforts in accomplishing this comprehensive study. However, in its current form, the narrative throughout this manuscript does not clearly reflect the data, which markedly reduced enthusiasm. Despite the reduced enthusiasm, I believe that adjustments to the overall narrative, rationale and detail of particular experimental approaches and statistical analyses would significantly improve the impact of the manuscript.

Firstly, the authors wish to thank the reviewer for the thorough review of this manuscript. The authors believe he/she raised some interesting comments which enhanced the value of the revised work. Please find the FIGURES FOR REVIEWER, REVISED MANUSCRIPT FIGURES, and REVISED MANUSCRIPT SUPPLEMENTARY FIGURES at the end of this document. All changes to the revised manuscript text are indicated in this document in **bold/underlined/grey highlight**.

Major

1) Narrative: The introduction is presented in the style of a general review of the literature. As such, the structure of the introduction does not accurately set up the study design and results narrative.

The introduction section was updated in the revised manuscript (see Lines 22-45).

2) Narrative: There needs to be a reworking of the statistics/narrative to fit the data. Currently most statistics are comparing the 2 mo timepoint to other timepoints. It may be advantageous to leverage the 6 mo timepoint since that is considered a fully developed "young adult" mouse and is more consistent with the literature in the cardiovascular aging field. The period between 2 and 6 months shows how CV function is changed throughout the maturation/development process in mice, but not aging per se.

The reviewer is correct in stating that 2-month old mice are not fully adult, and he/she makes an interesting point in separating out the effects of maturation versus aging. According to this reasoning, we agree that comparing all data statistically versus the youngest age group was an incorrect approach. However, we disagree with the reviewer that the 6 month old mice would be the optimal 'control' age. Using the information available on the website of The Jackson Laboratory regarding the life stages of C57Bl/6 mice *[1,2]*, the 'mature adult' stage is situated from 2-6 months of age (corresponding to an equivalent human age of 20-30 years). Therefore, the 6-month old group is already at the outer limit of what should be considered as 'mature adult'. The authors therefore believe it would be more appropriate to use the 4-month group as the 'control' age for statistical testing, and have updated all figures and result descriptions in the manuscript accordingly. Furthermore, a statement was added to the statistical methods to explain this approach (Lines 423-425): **"For the main aging experiment (2-24 months), post-hoc testing for the factor age was performed vs. the 4-month value unless otherwise stated, since this represents the first age included in the experiment which represents the mature adult stage.[17,18]"** Please note that, even when using the 4-month age as 'control', significant aging was observed in the 6-month group (including increased collagen and decreased elastin content), which is why the authors do not consider it would be advisable to compare all data to the 6-month old mice, where significant arterial aging is already occurring. This is consistent with clinical findings, which also show that PWV increases early in adult life, with significant PWV changes already from 20-29 years of age *[3-7]* (which is a similar age range as the 3-6 month old mouse age-range, designated by The Jackson Laboratory as 'mature adult'), as was stated in the manuscript discussion. We believe it strengthens our conclusions that the aging

effects described in the manuscript can be demonstrated versus 'mature adult' 4-month old mice instead of 'juvenile' 2-month old mice, and thus thank the reviewer for this excellent suggestion.

[1] THE JACKSON LABORATORY. Life span as a biomarker. Available: https://www.jax.org/research-and-faculty/research-labs/the-harrison-lab/gerontology/life-span-as-a-biomarker.

[2] THE JACKSON LABORATORY. 2017. When are mice considered old? Available: https://www.jax.org/news-and-insights/jax-blog/2017/november/when-are-mice-considered-old.

[3] AQUARO, G. D., CAGNOLO, A., TIWARI, K. K., TODIERE, G., BEVILACQUA, S., BELLA, G. D., AIT-ALI, L., FESTA, P., GLAUBER, M. & LOMBARDI, M. 2013. Age-dependent changes in elastic properties of thoracic aorta evaluated by magnetic resonance in normal subjects. Interactive CardioVascular and Thoracic Surgery, 17, 6.

[4] BOTTO, F., OBREGON, S., RUBINSTEIN, F., SCUTERI, A., NILSSON, P. M. & KOTLIAR, C. 2018. Frequency of early vascular aging and associated risk factors among an adult population in Latin America: the OPTIMO study. J Hum Hypertens, 32, 219-227.

[5] DIAZ, A., GALLI, C., TRINGLER, M., RAMIREZ, A. & CABRERA FISCHER, E. I. 2014. Reference values of pulse wave velocity in healthy people from an urban and rural argentinean population. Int J Hypertens, 2014, 653239.

[6] PEREIRA, E. N., VITORINO, P. V. D. O., SOUZA, W. K. S. B. D., PINHEIRO, M. C., SOUSA, A. L. L., JARDIM, P. C. B. V., REZENDE, J. M. & COCA, A. 2017. Assessment of Central Blood Pressure and Arterial Stiffness in Practicing Long-Distance Walking Race. International Journal of Cardiovascular Sciences, 30.

[7] REFERENCE VALUES FOR ARTERIAL STIFFNESS, C. 2010. Determinants of pulse wave velocity in healthy people and in the presence of cardiovascular risk factors: 'establishing normal and reference values'. Eur Heart J, 31, 2338-50.

3) Statistical analyses: All analyses were conducted relative to the 2 month time-point. This approach does not accurately represent the aging effect on the outcomes assessed. Rather, analyses should be conducted across all time-points. Furthermore, the most accurate representation of the aging effect would be to compare the 6 mo time-point (time-point representing the earliest stage of physical maturation [*i.e.*, young adulthood]) vs. the 24 month time-point [*i.e.*, older adulthood]).

Although it would be interesting to perform the statistical analysis across all time-points, the authors believe it would be impossible to represent these statistical results graphically in a clear and readable manner, considering the complexity of the data (*e.g.*, two-way ANOVA data using both 6 age groups and 4 distending pressures). Rather, the authors opted for the clean and easy-to-read approach of using a single control age group for the statistical testing. The statistical testing was thus updated throughout the manuscript (as the reviewer suggested) using the 4-month old mice as 'control' age group. Please refer to the previous reply (Question 2) for the reasoning behind the choice of 'control' age.

4) Narrative: Numerous techniques and abbreviations, such as VGCC and Cacna1C, are not well explained in the introduction or prior to use in the results section.

First use of abbreviations was updated throughout the manuscript. The authors hope this will improve the readability of the results.

5) Rationale: Clear rationale should be provided regarding the selection of mouse ages. Middle-aged for this mouse strain is ~18 months and the median lifespan for this strain is ~27 months – why weren't these ages used? Furthermore, it is not clear in the literature as to how vascular function changes through physical maturation (2-6 months in this mouse strain), so this is a novel component of the study; however, authors do not highlight this fact.

Again, we refer to the information available on the website of The Jackson Laboratory regarding the life stages of C57Bl/6 mice *[1,2]*. Using this information, the authors converted the mouse ages in the manuscript to their equivalent human ages as follows:

- mouse, 2 month (human, 16 years), 'juvenile';
- mouse, 4 month (human, 23 years), 'mature adult';
- mouse, 6 month (human, 30 years), 'mature adult';
- mouse, 9 month (human, 36 years), 'middle-aged';
- mouse, 12 month (human, 42.5 years), 'middle-aged';
- mouse, 24 month (human, 69 years), 'old'.

We have updated the discussion to clearly state these equivalent human ages and life stages of the mouse life span (Lines 169-175): "**The present study included mice of 2, 4, 6, 9, 12, and 24 months of age. This corresponds to equivalent human ages of 16, 23, 30, 36, 42.5, and 69 years, respectively. As follows, the study includes most mouse life phases including 'juvenile' (2-month old), 'mature adult' (4- and 6-month old), 'middle-aged' (9- and 12-month old), and 'old' (24-month old), and the study thus describes both the maturation (from 2 to 4 months of age) and aging (from 4 to 24 month of age) of male C57Bl/6 mice. The results in this study demonstrate that significant aortic aging (including increased collagen and decreased elastin content) occurs early in the aging process, starting at 6 months of age**. ~~6-Month old C57Bl/6 mice are usually regarded as 'adult' animals, and thus cannot be termed 'old' or 'aged'.~~ In (healthy) humans, PWV also increases early in adult life, …"

[1] THE JACKSON LABORATORY. Life span as a biomarker. Available: https://www.jax.org/research-and-faculty/research-labs/the-harrison-lab/gerontology/life-span-as-a-biomarker.

[2] THE JACKSON LABORATORY. 2017. When are mice considered old? Available: https://www.jax.org/news-and-insights/jax-blog/2017/november/when-are-mice-considered-old.

6) Rationale: There is no justification for the varying sample sizes in different figures or why there are different mouse number at different timepoints within a given figure.

Please note that the data presented in this manuscript are not paired measurements, but rather individual age groups which were each measured once at their respective end ages. The authors decided on this simple study design, to avoid the influences of repeated measurements, because- even though many of our measurements (blood pressure, echocardiography, aPWV) are non-invasive, they involve stress for the animals and - in the case of echocardiography and aPWV - additional rounds of anaesthesia. Furthermore, much of the data was obtained from the isolated *ex vivo* thoracic aorta, and since these measurements could not be performed as serial measurements, the authors wanted to ensure that *in vivo*, *ex vivo*, histological, and molecular measurements could be related within each mouse.

Off course the reviewer in correct in stating that there was a high variability between the sample sizes for each age group. This is so, because the C57Bl/6 mice in this study were actually the control mice in a larger study, looking at the effects of aging and arterial stiffening on neurodegeneration. In this study, age-related neurodegeneration was compared between aging mutant humanized amyloid

precursor protein (APP) overexpressing (hAPP23$^{+/-}$) Alzheimer disease mice (not published) and C57Bl/6 control mice (published separately by Hendrickx et al *[1]*). For the behavioural memory tests, a sample size of n=10 was planned. However, due to high mortality of the hAPP23$^{+/-}$ mice, some age groups (6 and 12 months of age) had to be repeated, which resulted in n-numbers of approximately 10 for the 2, 4, 9, and 24-month old mice, and n-numbers of approximately 20 in the 6 and 12-month old mice for the C57Bl/6 control groups.

Regarding the different sample sizes in different figures, this is due to the fact that all mice were included for the *in vivo* analyses, whereas fewer mice were included for the labour-intensive *ex vivo* testing, and an n=5 selection was used for histological/molecular analyses. This difference was clarified in the methods section (see Line 335-336, Line 358-360, Line 388-389).

[1] HENDRICKX, J. O., DE MOUDT, S., CALUS, E., DE DEYN, P. P., VAN DAM, D. & DE MEYER, G. R. Y. 2022. Age-related cognitive decline in spatial learning and memory of C57BL/6J mice. Behav Brain Res, 418, 113649.

7) Rationale: In the results section it would be helpful to explain why certain pressure ranges were selected for Peterson's modulus. How do those pressure ranges relate to mouse and human physiology?

The pressure range was selected to include a broad physiologically relevant pressure range (hypotensive, normotensive, hypertensive). This information was added to the results section (Lines 67-70): "~~**Next, E_p was studied over a pressure range from 60-100, 80-120, 100-140 to 120-160 mmHg.**~~ **Next, E_p was studied over a broad physiologically relevant pressure range from hypotensive (60-100 mmHg) to normotensive (80-120 mmHg), borderline hypertensive (100-140 mmHg), and hypertensive (120-160 mmHg) distending pressures.** The age-E_p relationship …"

Regarding blood pressure, mouse and human physiology is similar, with equal right atrial and mean systemic arterial pressures (*i.e.*, 5 mmHg and 100 mmHg, respectively) *[1,2].*

[1] HAMLIN, R. L. & ALTSCHULD, R. A. 2011. Extrapolation from mouse to man. Circ Cardiovasc Imaging, 4, 2-4.

[2] WOLINSKY, H. & GLAGOV, S. 1969. Comparison of abdominal and thoracic aortic medial structure in mammals. Deviation of man from the usual pattern. Circ Res, 25, 677-86.

8) Rationale: The rationale for using L-NAME in particular experiments is not well justified. If authors hypothesize that NO mediates the responses they are measuring, that should be clearly stated throughout.

The rationale of L-NAME use during *ex vivo* experiments was added throughout the manuscript results where applicable (see Lines 76-78, Lines 98-99,.Lines 104-105, and Lines 126-128).

9) Methods/Rationale: Better justification and rationale is needed to explain why certain methods such as CaV1.2 splice variant PCR and isometric reactivity were used to study aortic stiffness.

To clarify, aortic stiffness was only studied *in vivo* as aPWV and *ex vivo* as Peterson modulus. Measurements of isometric reactivity and the variant histological/molecular techniques were used to further investigate the mechanisms of aortic stiffening in spontaneously aging C57Bl/6 mice. The objective behind each method was now added to the start of each method subsection of the revised manuscript (see Lines 333-335, Line 351, Lines 380-381, Lines 394-395, and Lines 411-412).

10) Methods: Inclusion of tracking points in the PWV graphs by using lines in between the same mouse to ensure similar trends with age would be helpful to the audience.

As previously stated in the reply to Question 6, the authors want to stress that the data presented in this manuscript are not paired measurements, but rather individual age groups which were each

measured once at their respective end ages. This was clarified in the methods section as follows (Lines 318-320): "For the main experiment, male C57Bl/6 mice were used at the age of 2 (n=14), 4 (n=11), 6 (n=25), 9 (n=10), 12 (n=20), and 24 (n=8) months old**, which were each measured once at their respective end ages to avoid the influences of repeated measurements**."

11) Methods: Only one aortic cross-sectional image per group is included to represent the histology data. Rather one section per group should be provided for each sub analysis. This will enhance rigor and reproducibility.

The representative images of orcein, collagen type I, collagen type III, and myocardin stainings were updated to include a representative image for each age group (see Supplementary Figures 1, 2, 3, and 5).

12) Data interpretation: The PWV measures are a bit concerning, given that these are genetically identical mice who are not on intervention there is a very large spread (nearly 350 cm/sec at some time points). Please expand on this finding in the discussion/limitations section….Showing individual mouse trends in PWV (as mentioned above) could limit this concern.

As previously stated in the reply to Questions 6 and 10, the authors want to stress that the data presented in this manuscript are not paired measurements, but rather individual age groups which were each measured once at their respective end ages.

Regarding the spread of the PWV data, the authors want to note that this is an *in vivo* measurement, and is therefore highly dependent on many confounding factors (*e.g.*, blood pressure, heart rate, level of anaesthesia, circulating factors), as well as experimental factors (*e.g.*, exact site of the measurement on the abdominal aorta). Furthermore, as the manuscript includes aPWV measurements of close to 100 mice, these were obviously not done all at once, and seasonal and circadian rhythm changes may also underlie this spread. The authors were thus not much concerned by the spread of the data. The standard deviation of *in vivo* and *ex vivo* stiffness measurements is presented in Table 1 for Reviewer (based on the data in Figure 1a,b). Please note that in the 12-month age group (largest spread for aPWV), a standard deviation of 0.96 m/s was measured, which is 27.7% of the mean aPWV value. For *ex vivo* E_p, a standard deviation of 13.86 mmHg was measured, which is only 4.2% of the mean E_p value. Therefore, the authors believed the variation was largely due to *in vivo* confounding factors and experimental variation. As statistical testing takes into account the spread of the data, and considering the high sample sizes used in the study, the authors consider that the variation on the various measurements was not a limiting factor in the study.

Table 1 for Reviewer: Experimental spread of *in vivo* (aPWV) and *ex vivo* (E_p) stiffness measurements.

age (month)	mean (m/s)	SD (m/s)	SD/mean (%)	mean (mmHg)	SD (mmHg)	SD/mean (%)
2	1.97	0.38	19.3	290	23	8.1
4	2.61	0.68	26.0	307	12	4.0
6	2.94	0.72	24.6	319	17	5.4
9	3.73	0.80	21.5	322	23	7.1
12	3.48	0.96	27.7	327	14	4.2
24	4.87	0.95	19.4	368	17	4.5

Abbreviations: aPWV, aortic pulse wave velocity; E_p, Peterson modulus, SD, standard deviation

13) Data interpretation: Type-1 collagen is the primary isoform of collagen in arteries and does not show a clear "aging" pattern, while Type-3 collagen does. Throughout the discussion, authors describe these data collectively, when there were clearly differing age-related changes between the two isoforms.

The main fibril-forming collagens are types I, III, and V, of which types I and III are most involved in imparting strength to the vessel wall *[1,2]*. Therefore, it was indeed surprising to see that collagen types I and III displayed a different age-related trend. We thank the reviewer for noting that this was not highlighted in the original manuscript discussion. A statement on this difference was added to the

discussion of the revised manuscript (Lines 193-200): "The present study described marked aortic ECM changes with age, including increased collagen and decreased elastin content, which represent a hallmark ECM response to ageing.[27-29] **Interestingly, although collagen types I and III (which are the main fibril-forming collagens involved in imparting strength to the vessel wall[30,31]) were both increased with age, different trends were observed during the aging process. For collagen type III, a gradual increase with age was observed (similar to the observed increase in passive aortic stiffness), whereas collagen type I was increased in 6-24 month old mice versus 2-4 month old mice (similar to the observed increase in aortic contractility). These results suggest a variant role of collagen types I and III in aortic physiology and aging**."

[1] JACOB, M. P., BADIER-COMMANDER, C., FONTAINE, V., BENAZZOUG, Y., FELDMAN, L. & MICHEL, J. B. 2001. Extracellular matrix remodeling in the vascular wall. Pathol Biol (Paris), 49, 326-32.

[2] WAGENSEIL, J. E. & MECHAM, R. P. 2009. Vascular extracellular matrix and arterial mechanics. Physiol Rev, 89, 957-89.

14) Narrative/Data interpretation: Previous lifelong vascular function studies have been conducted in this same mouse strain (PMID: 33103241). The results of this previous lifelong study should be considered in the context of the present study. In particular, this previous publication highlights the role of vascular mitochondrial oxidative stress and inflammation and reduced nitric oxide bioavailability as mediators of vascular aging (*i.e.*, mechanisms underlying vascular aging). Furthermore, this publication showed a clear phenotype for age-related endothelial dysfuncton(where the current manuscript did not), albeit in a pressurized carotid artery model (which is suggested to be more translational than the aortic ring model for assessing endothelium-dependent dilation).

We thank the reviewer for providing the PMID 33103241 publication. It was added to the discussion of the revised manuscript (Lines 246-249): "Preclinical studies in accelerated aging models have indeed identified endothelial dysfunction as a key mechanism of aging.[56] **Similarly, another recent aging study demonstrated impaired NO function during aging of C57Bl/6 mice, which was accelerated by Western-type diet and ameliorated by aerobic exercise.[53]**"

15) Data interpretation: The fact that changes in PWV precede any changes in blood pressure, is certainly interesting (which was also shown in the lifelong study highlighted above); however, PWV is considered the "gold standard" technique for assessing large elastic artery stiffness, while tail-cuff blood pressure is not a "gold-standard" technique. Further, there is not enough data to claim that endothelial function is not relevant to arterial dysfunction. This statement can be made when specifically referring to the mice in this study but it not all-encompassing to other mouse strains or even other cohorts.

Regarding the tail-cuff BP measurement: The reviewer is correct in stating that the tail-cuff method for blood pressure measurement is not considered a gold-standard technique. Please note, however, that the authors never state that PWV precedes the change in blood pressure (in general), but that we always specify peripheral blood pressure. Considering the close relationship between arterial stiffness and hypertension, it would not be surprising to the authors to see that central blood pressure (as measured using more sensitive methods such as invasive catheterization or telemetry) would have been increased earlier during the aging process, especially when considering that the hemodynamic alterations associated with aortic stiffening (*i.e.*, early return of the backward travelling pressure wave and systolic BP augmentation) directly lead to increased systolic aortic pressure. However, the authors wanted to ascertain at which stage during the aging process the arterial tree was no longer able to compensate for increased aortic pressure, resulting in peripheral blood pressure alterations. This would be indicative of the stage in the aging process where organ damage could occur in the highly perfused tissues (*e.g.*, kidney, brain). That is the reason why the authors chose the tail-cuff method for peripheral blood pressure measurement, rather than a more sensitive method for central blood

pressure measurement. Furthermore, when considering the use of BP or PWV measurement in standard clinical practice, the authors consider it is more relevant to include peripheral rather than central blood pressure assessments in aging studies, as blood pressure is also measured peripherally in the clinic. To clarify, the following text was added to the discussion (Lines 282-285): "**The present study measured blood pressure using the tail-cuff method. Although this is not a gold standard method for blood pressure assessment, it allows for measurement at a peripheral site, which is more relatable to the clinical setting and indicative of the risk of pulsatile damage to the peripheral organs.**"

Regarding the conclusion regarding endothelial dysfunction in arterial aging: To accommodate the reviewer's comment, the study conclusion was updated as follows (Lines 305-307): "… Minimal changes in endothelial NO signalling were observed, suggesting that spontaneous arterial ageing in **the studied male** C57Bl/6 mice was due to ECM and VSMC signalling changes rather than altered EC function."

Minor

16) Methods: The authors do not clearly identify the mouse strain, C57BL/6 (J? or N?), where the strain was obtained, and the specific mouse chow used in the study. Details such as these are essential for enhancing rigor and reproducibility.

As stated in the methods section: "All mice were bred and housed in the animal facility of the University of Antwerp, …", all animals were bred in-house. Although these C57Bl/6 mice were originally purchased as C57Bl/6J, the authors are uncomfortable with assigning the 'J' specification as this strain of C57Bl/6 mice has since been bred for >10 years in our own animal facility. Therefore, the more general term C57Bl/6 was used. Regarding the chow, the methods were updated to state (Line 318): "… and had free access to water and standard chow **(Ssniff® Rat/Mouse maintenance 10 mm, V1534-000)**."

17) Narrative: Line 37: Define "spontaneously aging."

Spontaneously aging was defined in the revised manuscript as follows (Line 48): "In the present study, a longitudinal cardiovascular characterization of spontaneously **(*i.e.*, age-dependent)** aging C57Bl/6 mice is presented …"

18) Grammar: Line 45, "aortic" should not be capitalized.

Corrected.

19) Narrative: Line 45-46, aPWV is not a marker but the gold-standard method to assess aortic stiffness.

Manuscript updated as follows (Line 57): "*In vivo* aortic pulse wave velocity (aPWV~~, marker of aortic stiffness~~) increased time-dependently, …"

20) Data interpretation: It would be informative for the reader if authors would highlight the individual data. The manuscript states that PWV increased by 250%, which doesn't appear to be the case for all mice.

Please refer to the previous comments on the fact that this manuscript does not show inter-animal comparisons over time. Therefore, this statement refers to the change in mean aPWV, which is consistent with the data.

21) Data interpretation: Line 49: "age-related stiffening" should not be assessed in 2 vs. 6 mo old mice. This time-frame represents physical maturation, not aging.

Please refer to the previous comments on the statistical analysis (Question 2 and 3) and how the manuscript was revised to accommodate these comments.

22) Methods: Line 64-65,69,76,88, compound concentrations not necessary; include in the materials and methods section

Updated as suggested by the reviewer in the revised manuscript.

23) Narrative: Line 66,83, NOS should be spelled out before and defined in the previous sentence where it was introduced

As mentioned in the reply to Question 4, first use of abbreviations was updated throughout the revised manuscript.

24) Rationale/ Data interpretation: Line 70, why are mice starting at 6 mo considered older (6 months is equivalent to ~30 in human years)?

In this context, "older" is meant as older than the 2- and 4-month old mice, and not as 'old' or 'aged'. As 6-months is numerically higher, the authors do not consider this is incorrect, although we understand this can be confusing. Therefore, the text was updated as follows (Lines 85-86): "…, *i.e.*, increased contraction-dependent aortic stiffening in the **6-24 month old** versus **2-4 month old** mice (Figure 3c)."

25) Rationale: Line 80 & Figure 4A, why were these age groups chosen?

The exact age groups were largely chosen for practical reasons (availability and planning), but can certainly serve as model ages for 'adult' and 'old' mice, as specified in the revised manuscript (Lines 95-96): "Age-dependent VGCC activity was further assessed in a separate group of **adult (5 month old)** and **old (26 month old)** mice."

26) References: Lines 112-119, the references are not properly inserted.

Cross-references were corrected.

27) Narrative: Line 227, "BP" should be spelled out or defined previously.

As mentioned in the reply to Question 4, first use of abbreviations was updated throughout the revised manuscript.

28) Narrative: Line 242: Define "carotid-femoral PWV."

As mentioned in the reply to Question 4, first use of abbreviations was updated throughout the revised manuscript.

29) Narrative: Line 247: "…affirming the use of aortic stiffness as an early marker of CVD monitoring" is a very broad statement. This can only be described in the context of this mouse model.

The authors do not agree that using arterial stiffness as a marker in CVD monitoring is only applicable to the presented study. It has been observed in several studies, both in animal studies and clinical studies, that PWV is indicative of cardiovascular disease (see manuscript discussion), leading the ESC/ESH to adopt PWV measurement in its guidelines for cardiovascular risk evaluation in 2007. Therefore, the authors only state that our data is line with what is already recommended clinical practice, even though the guideline is not implemented routinely in the clinic. To avoid confusion, we have updated this sentence as follows (Lines 281-828): "…, affirming the use of aortic stiffness as an early marker in CVD monitoring **as recommended by the ESC/ESH guidelines**."

30) Figures/Methods: Figure 3E statistical symbols do not directly relate to legend below.

Figure legend updated as follows: "…, and calculated absolute BAY-K8644 effect (**circles,** E). …"

31) Figures/Rationale: Figure 5B lack of rationale for comparing 8 to 24 mos.

This was done to indicate where the overall ANOVA significance value <0.05 (actual value: p= 0.0338) originated from, as this was mainly driven the difference between the 9-month old and 24 month old mice. When performing a post-hoc analysis comparing all ages to all other ages, this was the only comparison that was significant. All others were above p=0.1000 (see Table 2 for Reviewer). To clarify, the following sentence was added to the results description (Lines 122-124): "**A statistically significant difference in ACh IC_{50} was only obtained when comparing the 24-month values to the 9-month values, as illustrated in Figure 5b.**"

Table 2 for Reviewer: Overview table of ANOVA post-hoc testing of acetylcholine IC_{50}.

Tukey's multiple comparisons test	Mean Diff	95,00% CI of diff	Significant?	Summary	Adjusted P Value
2 vs. 4	-0.01789	-0.4109 to 0.3751	No	Ns	>0.9999
2 vs. 6	-0.02954	-0.3900 to 0.3309	No	Ns	0.9999
2 vs. 9	0.1882	-0.2136 to 0.5900	No	Ns	0.7405
2 vs. 12	0.04047	-0.3331 to 0.4141	No	Ns	0.9995
2 vs. 24	-0.3083	-0.7332 to 0.1166	No	Ns	0.2844
4 vs. 6	-0.01165	-0.3500 to 0.3267	No	Ns	>0.9999
4 vs. 9	0.2061	-0.1760 to 0.5882	No	Ns	0.6109
4 vs. 12	0.05836	-0.2940 to 0.4107	No	Ns	0.9965
4 vs. 24	-0.2904	-0.6967 to 0.1159	No	Ns	0.3003
6 vs. 9	0.2177	-0.1307 to 0.5662	No	Ns	0.4504
6 vs. 12	0.07	-0.2456 to 0.3856	No	Ns	0.9864
6 vs. 24	-0.2787	-0.6536 to 0.09618	No	Ns	0.2592
9 vs. 12	-0.1477	-0.5098 to 0.2143	No	Ns	0.8355
9 vs. 24	-0.4965	-0.9112 to -0.08170	Yes	*	0.0101
12 vs. 24	-0.3487	-0.7363 to 0.03881	No	Ns	0.1016

Abbreviations: ANOVA, analysis of variance; CI, confidence interval; diff, difference; ns, non-significant.

FIGURES FOR REVIEWER

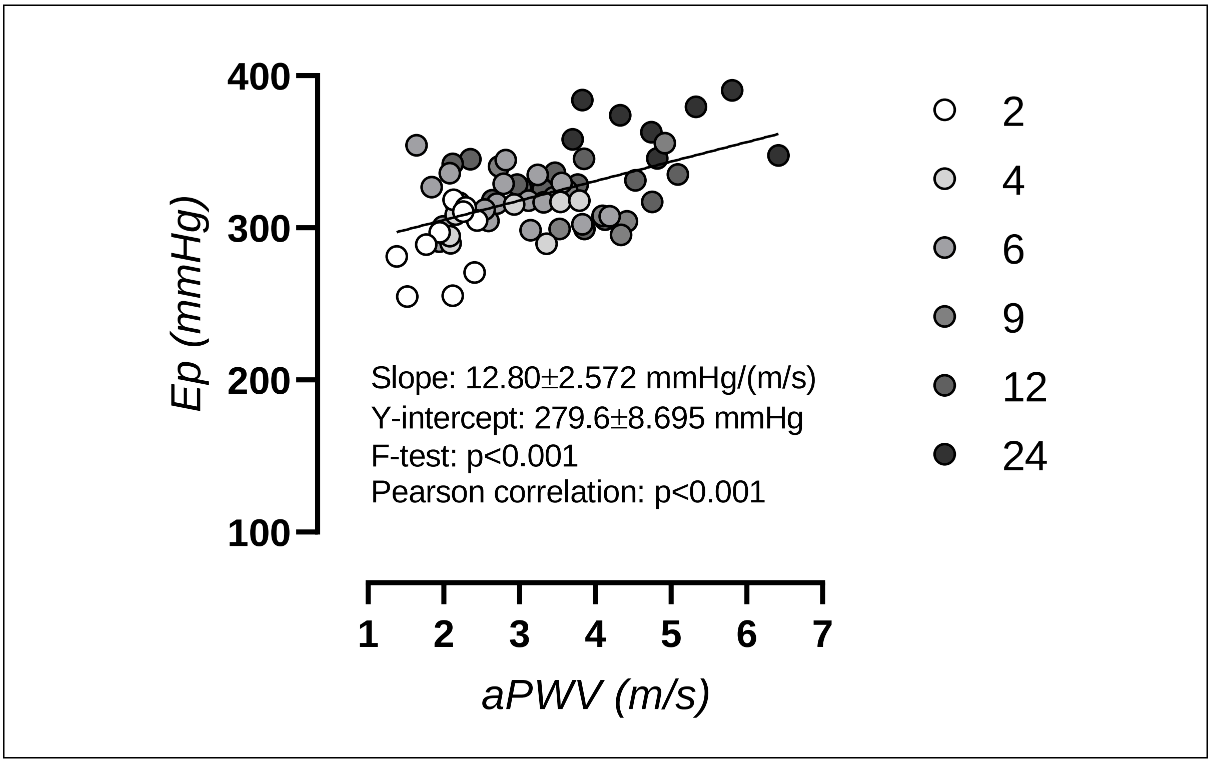

Figure 1 for Reviewer: Correlation of *in vivo* aPWV and *ex vivo* E_p (baseline, 80-120 mmHg) in male C57Bl/6 mice aged 2 to 24 months old. Slope and Y-intercept of a linear regression analysis is shown. Statistical analysis using an F-test (slope significantly non-zero) and Pearson correlation test.

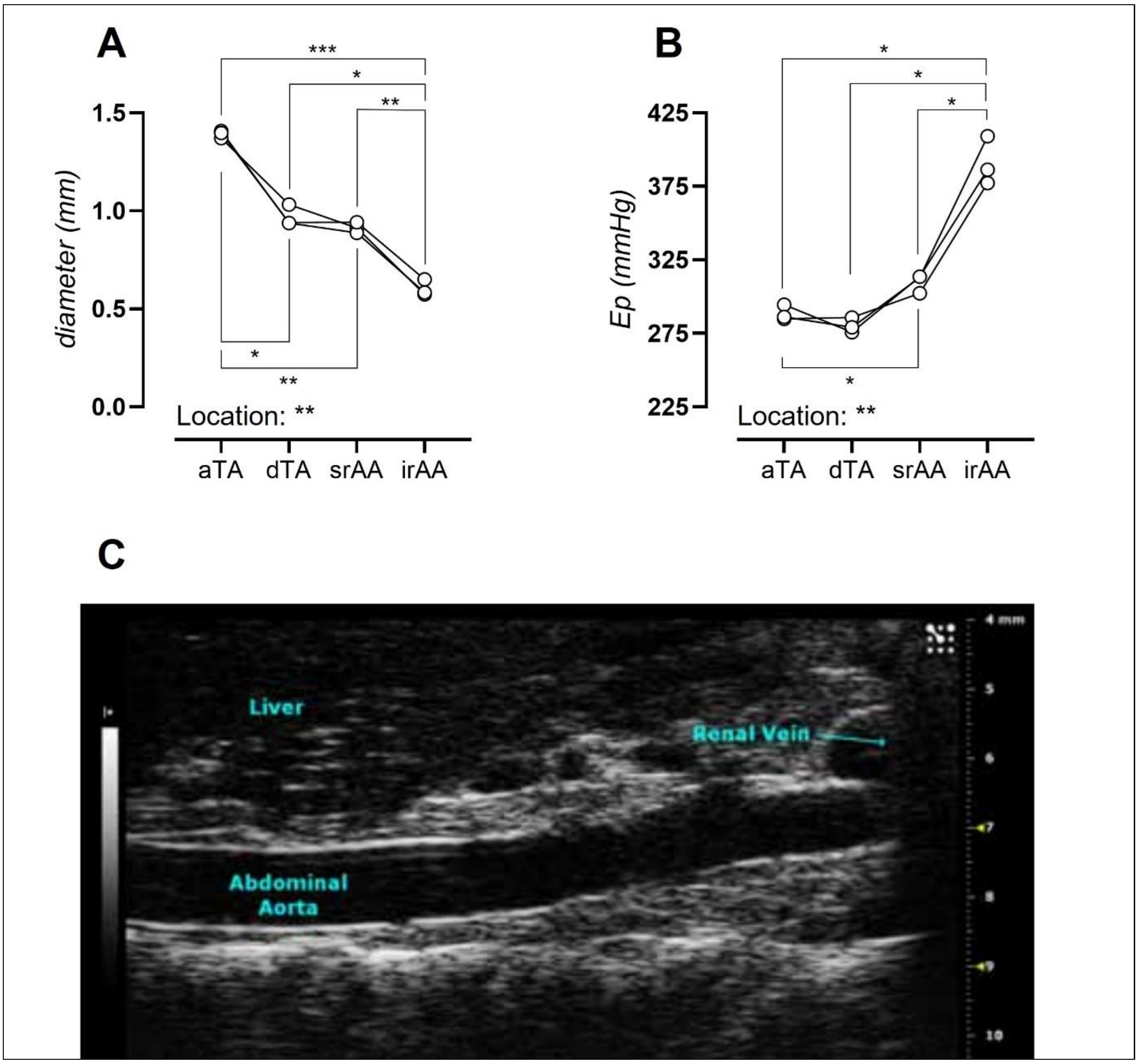

Figure 2 for Reviewer: *In vivo* and *ex vivo* measurements of arterial stiffness. Baseline aortic diameter at 80 mmHg (A) and Peterson modulus at 80-120 mmHg (E_p, B) distending pressure was shown for the ascending thoracic aorta (aTA), descending thoracic aorta (dTA), suprarenal abdominal aorta (srAA), and infrarenal abdominal aorta (irAA). A representative B-mode image of the abdominal aorta on ultrasound analysis was shown, indicating the site of aPWV measurement using the 'abdominal aorta' notation (C). Statistical analysis in A,B using repeated-measures one-way ANOVA. Overall significance (bottom of graph) and post-hoc significance (in graph) are listed. *, $p<0.05$; **, $p<0.01$; *** $p<0.001$.

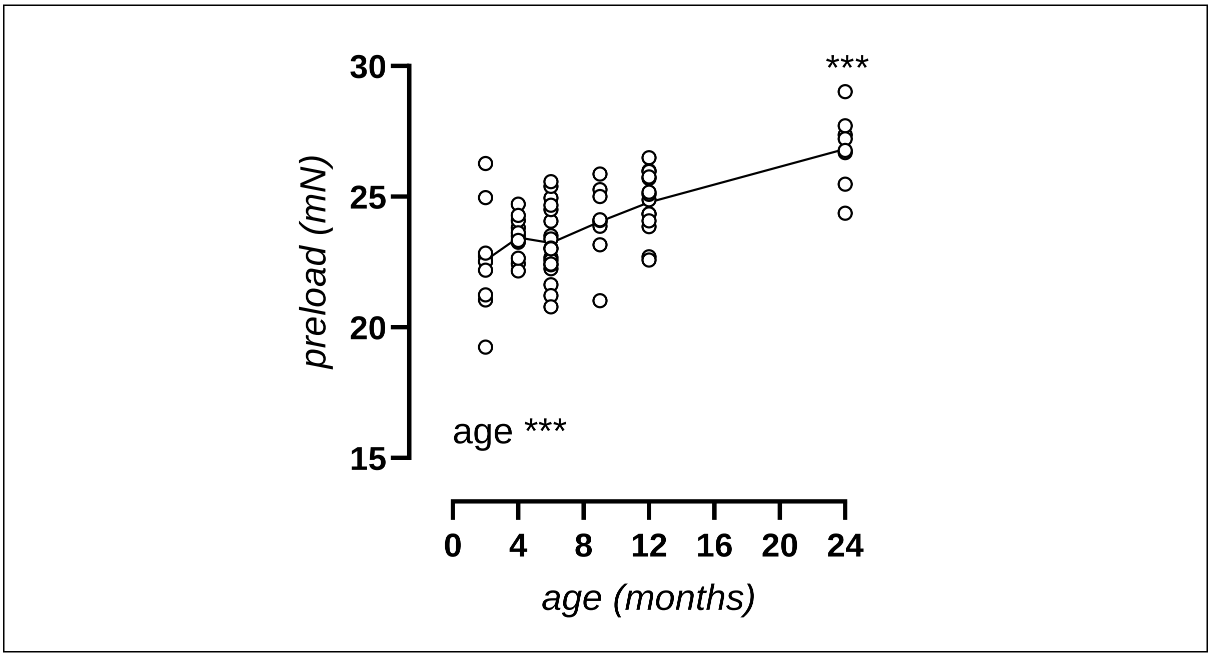

Figure 3 for Reviewer: Post-hoc analysis of the preload applied to obtain a calculated mean 100 mmHg distending pressure. Statistical analysis using one-way ANOVA. Overall significance (bottom of graph) and post-hoc significance vs. 4 month value (in graph) are listed. *** p<0.001.

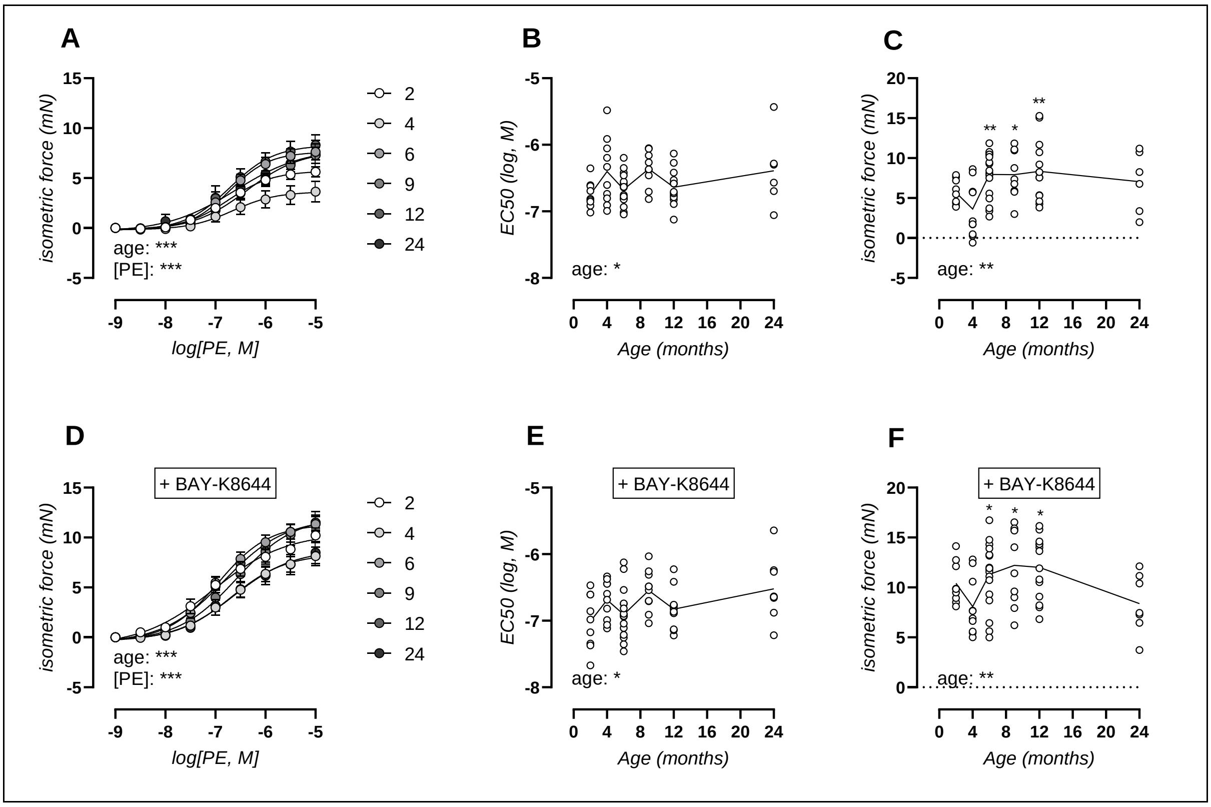

Figure 4 for Reviewer: Analysis of phenylephrine concentration response curves. Contractions were elicited by cumulative concentrations of PE-in the absence (A-C) and presence (D-F) of BAY-K8466. Concentration-response curves (A,D) and calculated values of EC_{50} (B,E) and maximal effect (C,F) are shown. Statistical analysis using one-way (B,C,E,F) or two-way ANOVA (A,D). Overall significance (bottom of graph) and post-hoc significance vs. 4 month value (in graph) are listed. No post-hoc significance was listed in A,D. *, p<0.05; **, p<0.01; *** p<0.001.

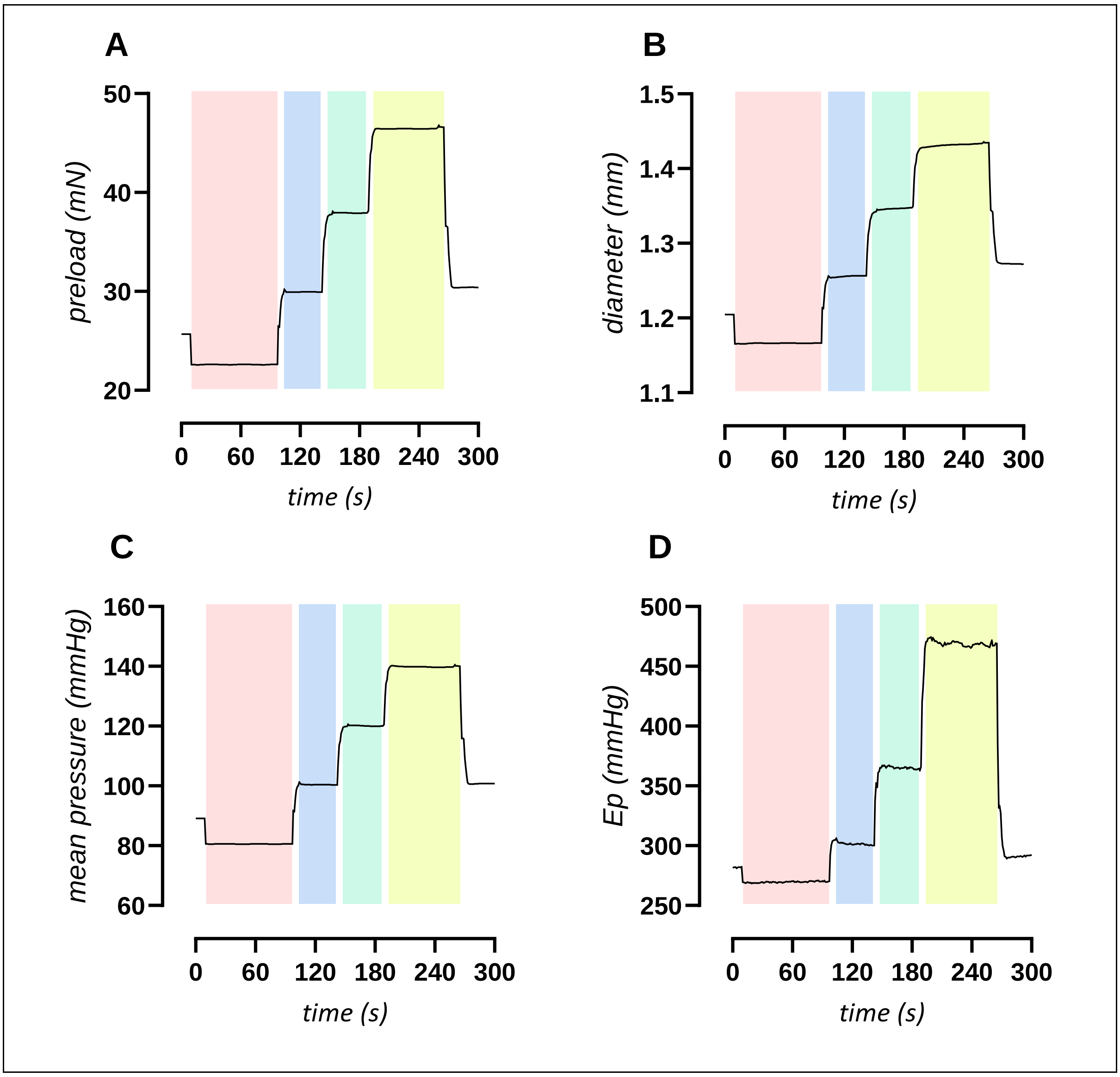

Figure 5 for Reviewer: Representative tracing of pressure-E_p curve recording. Measurement of force (A) and diameter (B) were used to calculate distending pressure (C) and Peterson modulus (E_p, D). Preload was adjusted to obtain 60-100 mmHg (red background), 80-120 mmHg (blue background), 100-140-mmHg (green background), and 120-160 mmHg (yellow background) pressure oscillations. On average, pressure-E_p curves were measured over a 5-minute period.

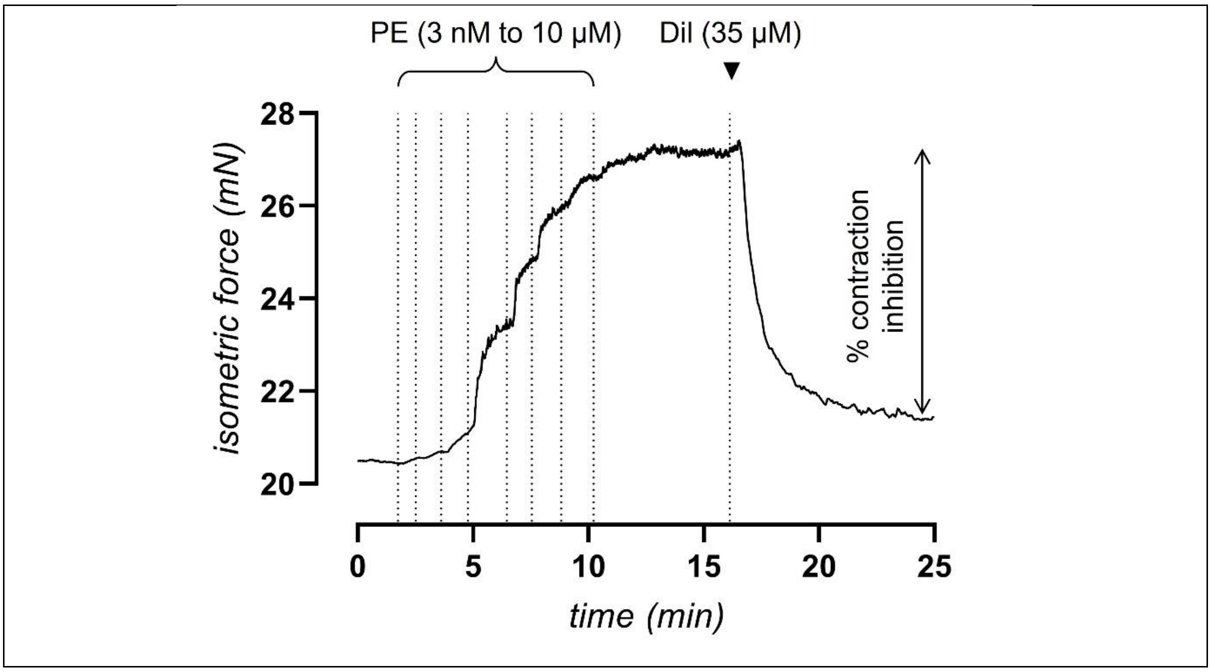

Figure 6 for Reviewer: Representative tracing of the PE-induced isometric contractions and diltiazem-induced isometric relaxation. Aortic contractions were induced by concentration response stimulation with PE (3 nM to 10 µM). When a steady-state contraction was reached, 35 µM diltiazem was added to block VGCC-dependent calcium entry. The effect of diltiazem was assessed as the percentage contraction inhibition. A baseline preload of approximately 20 mN was applied and the measurement was performed over 25 minutes in total.

REVISED MANUSCRIPT FIGURES

With the exception of Figure 5, all figures in the manuscript were updated according to Question 2 of Reviewer 3 (*i.e.*, updating the post-hoc statistical testing of ANOVA tests versus the 4 month instead of the 2 month age group). Please find the updated figures below:

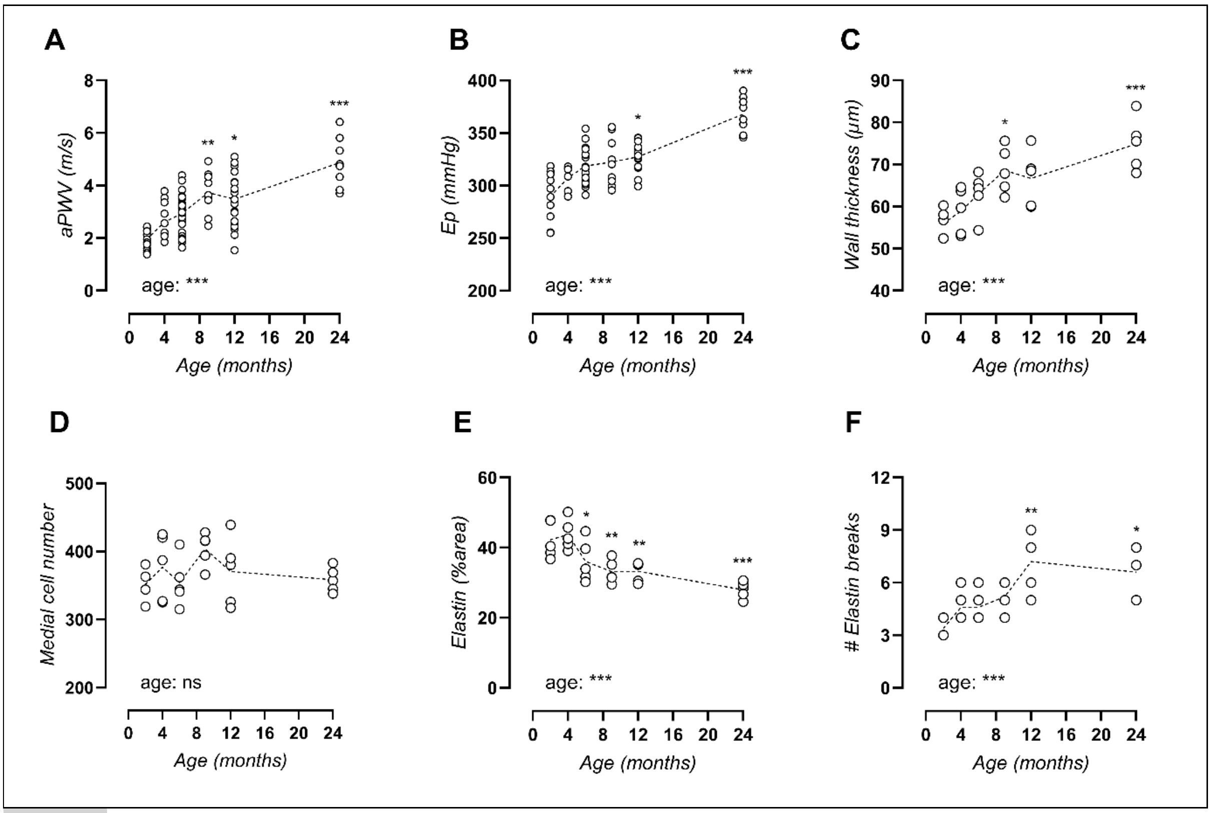

Figure 1: Age-related aortic stiffening in C57Bl/6 mice. Aortic stiffness was measured in vivo as aPWV (A) and ex vivo in the isolated thoracic aorta at isobaric conditions of 80-120 mmHg as Peterson modulus (Ep, B). Wall thickness was determined on orcein-stained aortic tissue sections (C) and total medial cell number was counted on haematoxylin-stained aortic tissue sections (D). Orcein staining was used to quantify elastin positive area (E) and counting of elastin breaks (F). Each symbol represents an individual biological repeat, with n>8 (A,B) and n=5 (C-F). Statistical analysis using one-way ANOVA. Overall significance (bottom of graph) and post-hoc significance vs. **4** month value (in graph) are listed. * p<0.05, ** p<0.01, *** p<0.001.

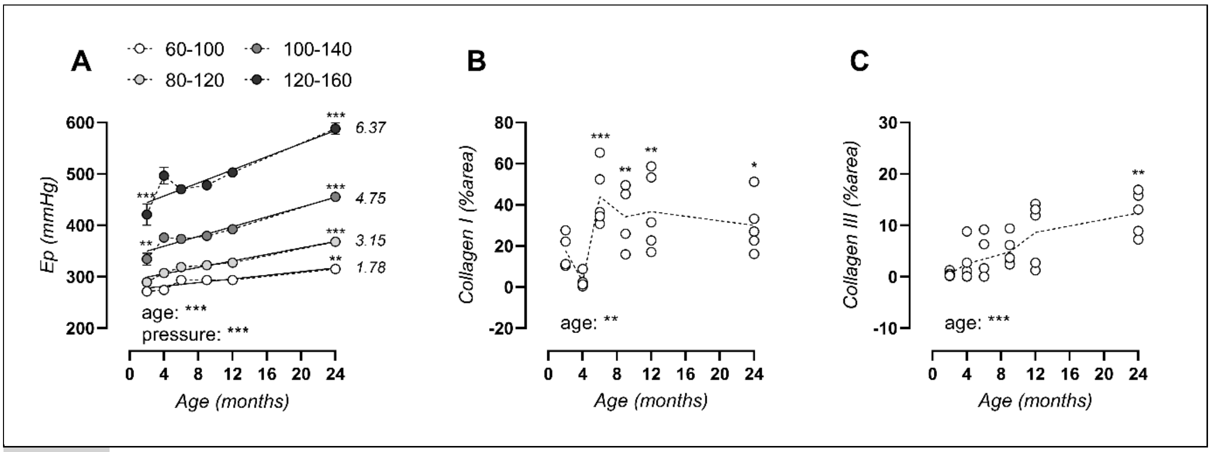

Figure 2: More pronounced aortic stiffening at high distending pressure corresponds to increased levels of types I and III collagen. Peterson modulus expressed in function of age for increasing distending pressure from 60-100, 80-120, 100-140, to 120-160 mmHg (A). Values of slope from linear regression analysis are listed on the right for each pressure. Histological assessment of area positivity for type I and II collagen (B-C). Data are listed as mean±SEM (n>8) (A) or each symbol represents an individual biological repeat (n=5) (B-C). Statistical analysis using two-way ANOVA (A) or one-way ANOVA (B-C). Overall significance (bottom of graph) and post-hoc significance vs. 4 month value (in graph) are listed. * p<0.05, ** p<0.01, *** p<0.001.

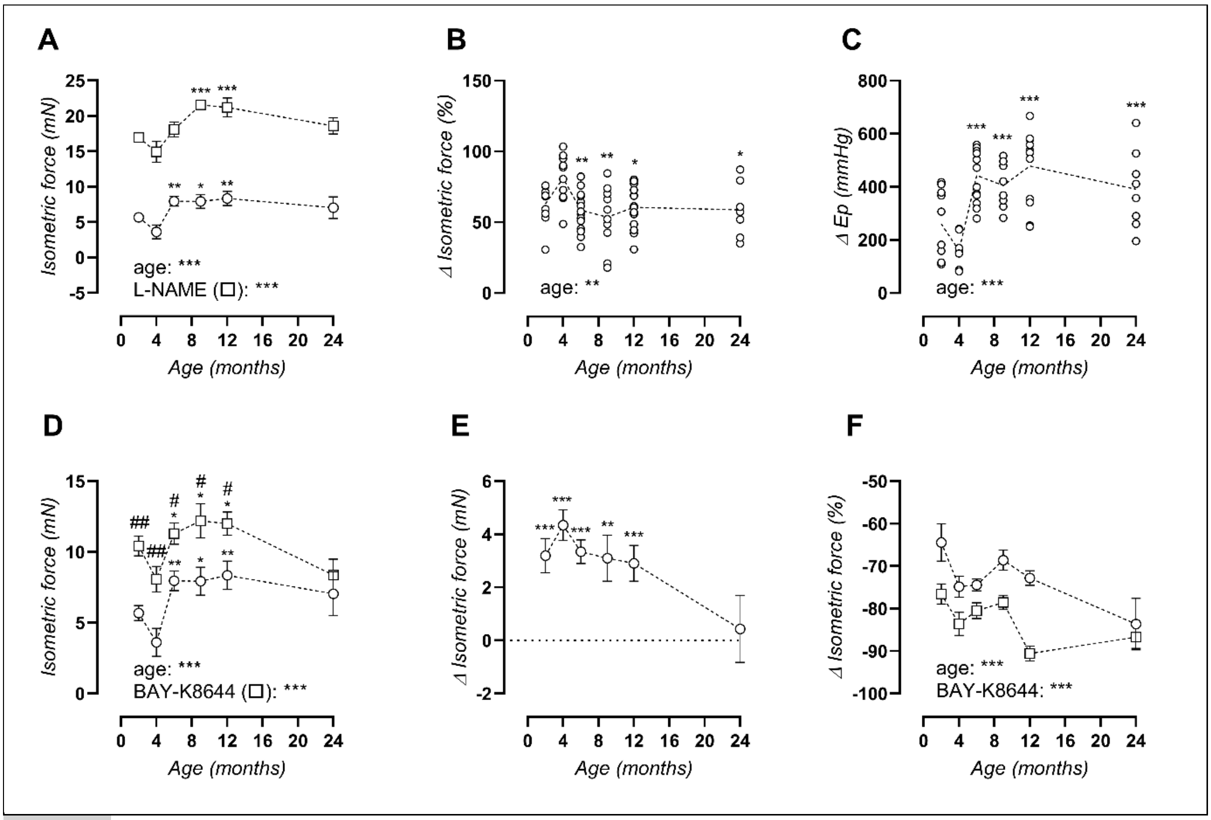

Figure 3: Increased PE-contractility and VGCC alterations. Maximal PE contraction in the absence (circles) and presence (squares) of L-NAME (A). Basal NO levels, quantified as the relative increase in PE contraction after addition of L-NAME (B). Contraction-dependent aortic stiffening by addition of 2 µM PE + 300 µM L-NAME at isobaric 80-120 mmHg pressure (C). Maximal PE contractions, as obtained from non-linear regression of the PE concentration response curves, in the absence (circles) and presence (squares) of 30 nM BAY-K8644 (D), and calculated absolute BAY-K8644 effect (circles, E). Contraction-inhibition by diltiazem in the absence (circles) or presence (squares) of BAY-8644 (F). Statistical analysis using two-way ANOVA (A,D,F), one-way ANOVA (B,C) or one-sample t-test vs. 0 (E). Data are listed as mean±SEM (A,D-F, n>8) or each symbol represents an individual biological repeat (B,C, n>8). Overall significance (bottom of graph) and post-hoc significance vs. 4 month value (in graph) are listed. * p<0.05, ** p<0.01, *** p<0.001. In D, post-hoc comparison for BAY-K8644 effect is listed as # p<0.05, ## p<0.01.

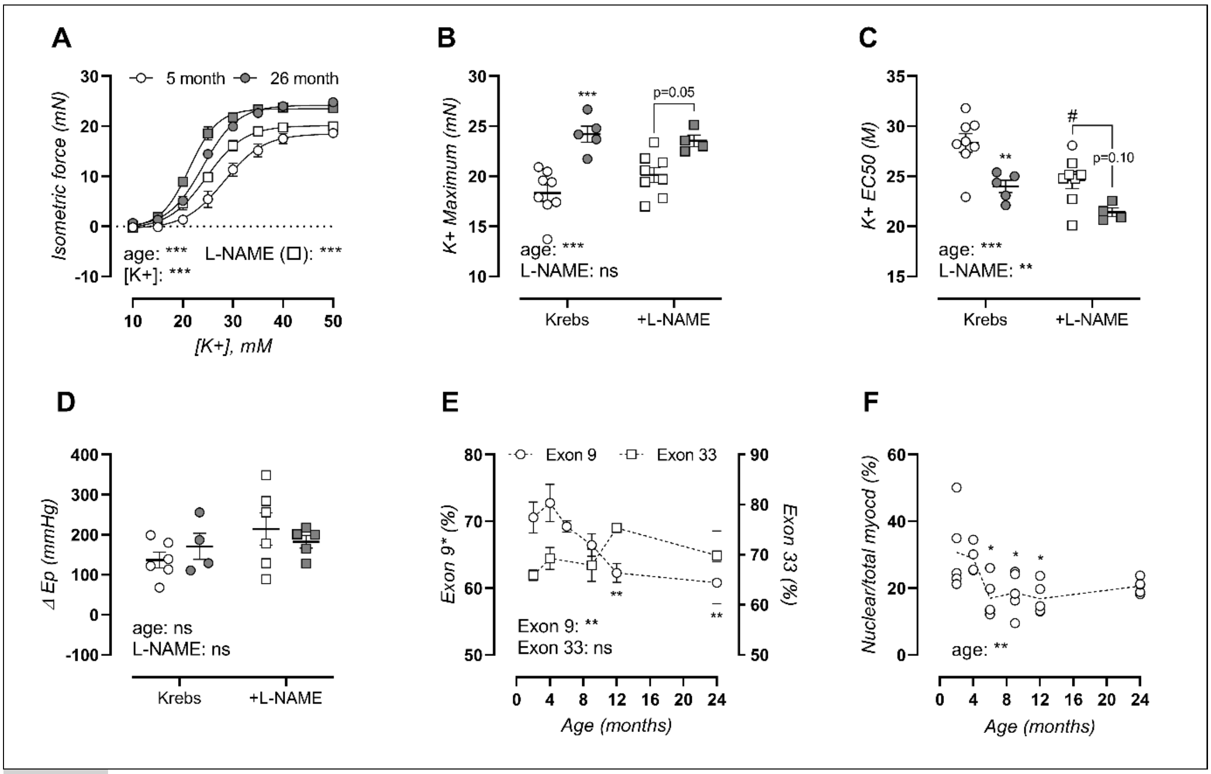

Figure 4: Functional and molecular VGCC phenotyping. Potassium concentration-response curves of 5 and 26 months old mice were plotted in the absence (circles) and presence (squares) of L-NAME (A). Non-linear regression analysis was used to calculate the maximal effect (B) and EC$_{50}$ value (C). 50 mM potassium-induced contraction-dependent change in Peterson modulus at calculated isobaric 80-120 mmHg distending pressure was measured in the absence and presence of 300 µM L-NAME (D). Splice variation was assessed for CaV1.2 exon 9* and exon 33, and quantification of the %mRNA with inclusion of exon 9* or exon 33 is shown (E). The fraction nuclear/total myocardin in the media was assessed using immunohistochemical staining, as a marker of VSMC phenotype regulation (F). Data are listed as mean±SEM (A,E, n>5) or each symbol represents an individual biological repeat (B-D,F, n>5). Statistical analysis using three-way ANOVA (A), two-way ANOVA (B-D), or one-way ANOVA (E,F). Overall significance (bottom of graph) and post-hoc significance vs. 4 month value (in graph) are listed (post-hoc significance was not added in A). ** p<0.01, *** p<0.001. In C and D, post-hoc comparison for L-NAME effect is listed as # p<0.05.

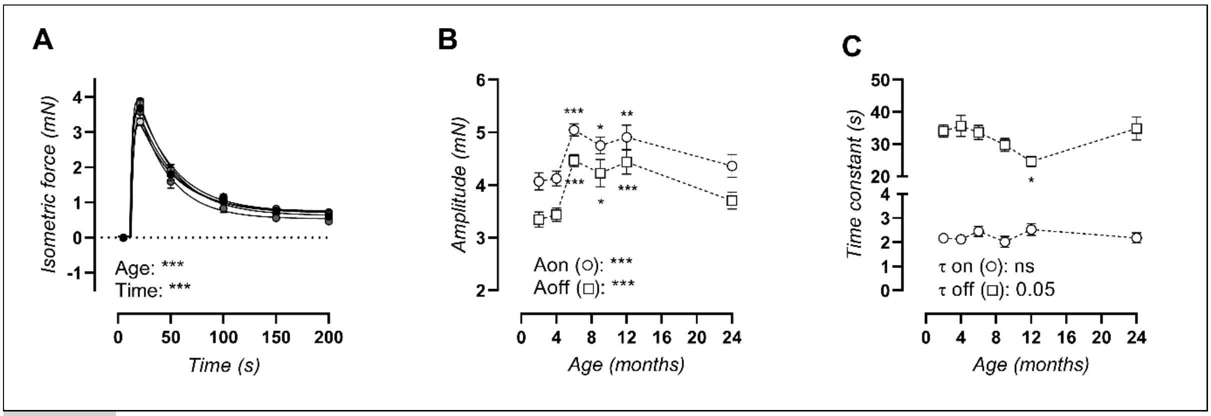

Figure 5: Altered transient SR-mediated contractions. A tracing of the transient SR-mediated contraction (A) was fitted with a bi-exponential regression equation encompassing the amplitude of the upward (A_{on}) and downward (A_{off}) phase (B), as well as the time constant of the upward (τ_{on}) and downward (τ_{off}) phase (C). Statistical analysis using two-way ANOVA (A) or one-way ANOVA (B,C). Data are listed as mean±SEM (n>8). Overall significance (bottom of graph) and post-hoc significance vs. 4 month value (in graph) are listed (post-hoc significance was not added in A). * p<0.05, ** p<0.01, *** p<0.001.

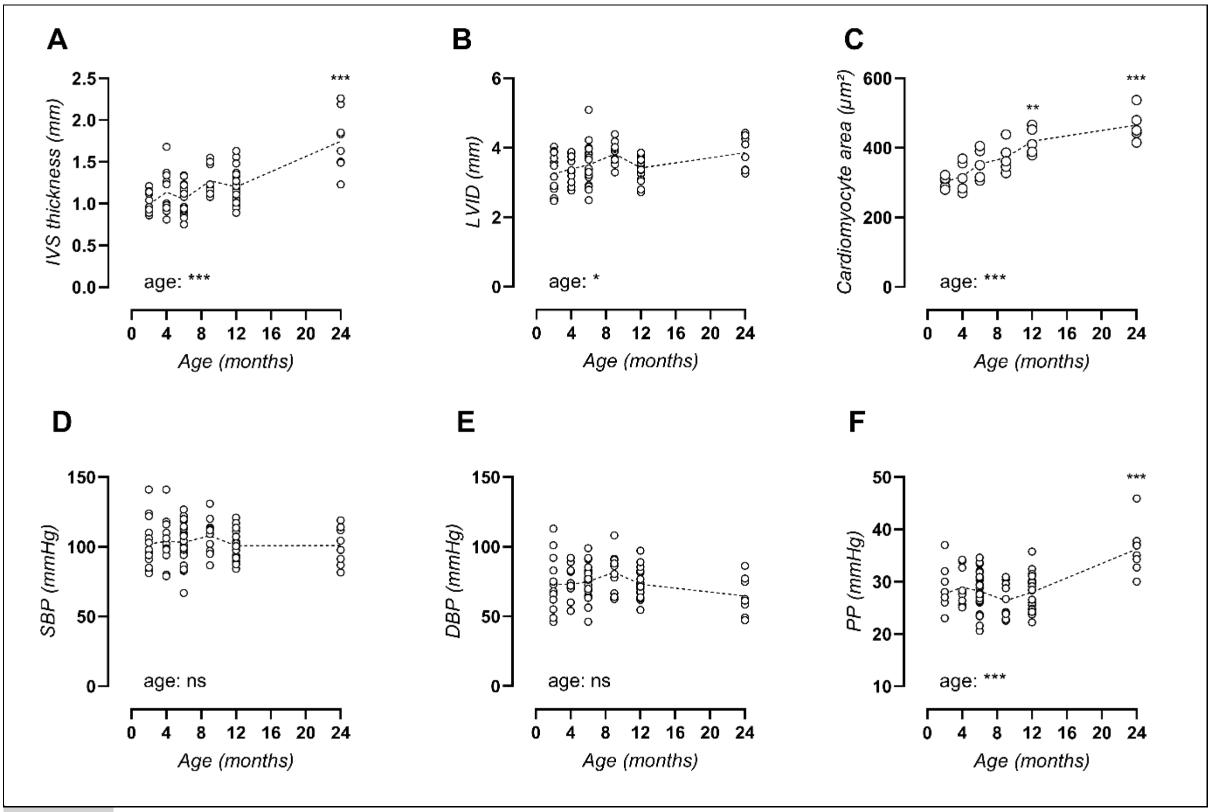

Figure 6: Arterial-stiffness precedes cardiac disease and peripheral blood pressure alterations. Echocardiograms were used to assess interventricular septum (IVS) thickness (A) and LV internal diameter (LVID, B). Cardiomyocyte cross-sectional area was studied using immunohistochemical staining for laminin (C). Peripheral systolic blood pressure (SBP, D), diastolic blood pressure (DBP, E) and pulse pressure (PP, F) were measured. Each symbol represents an individual biological repeat (n>8).Statistical analysis using one-way ANOVA. Overall significance (bottom of graph) and post-hoc significance vs. 4 month value (in graph) are listed. *** p<0.001.

REVISED MANUSCRIPT SUPPLEMENTARY FIGURES

Supplementary Figures 1-3 and 5-6 were updated according to Question 9 of Reviewer 2 and Question 11 of Reviewer 3 (*i.e.*, updating the scale bars and including all age groups in the representative histological images). Furthermore, a new Supplementary Figure 7 was added to clarify the method of pressure-E_p measurements according to Question 4 of Reviewer 2. Please find the updated figures below:

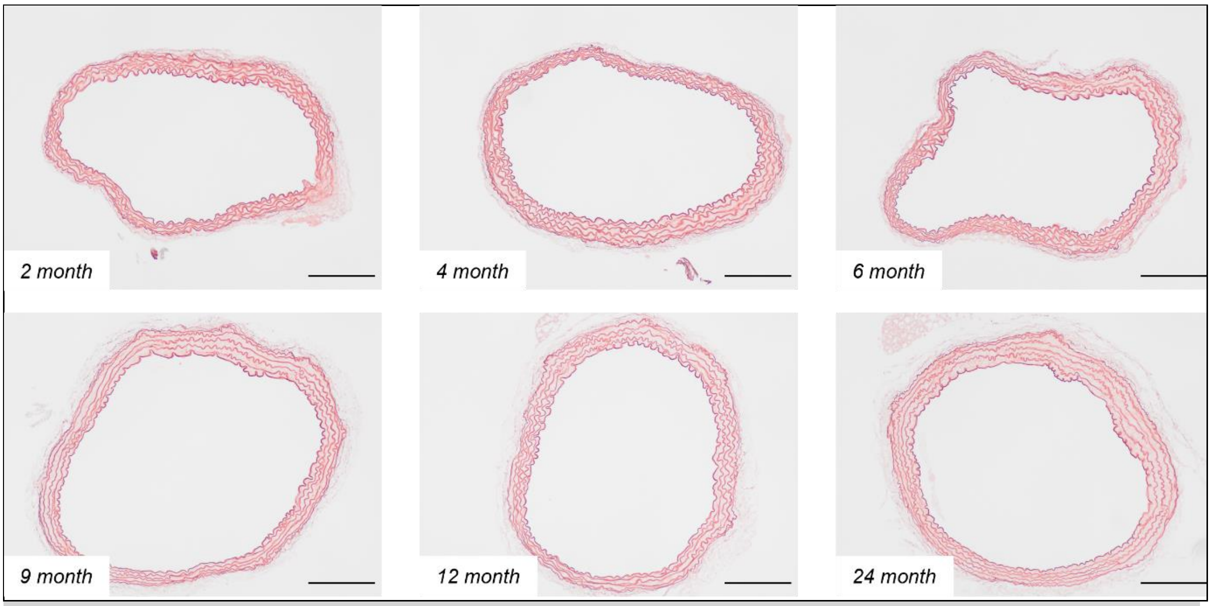

Supplementary Figure 1: Histological evaluation of aortic structure in aging C57Bl/6 mouse aortic tissue. Representative images of orcein-stained aortic tissue of 2, 4, 6, 9, 12, and 24 month old mice. Scale bar (bottom right) represents 200 µm.

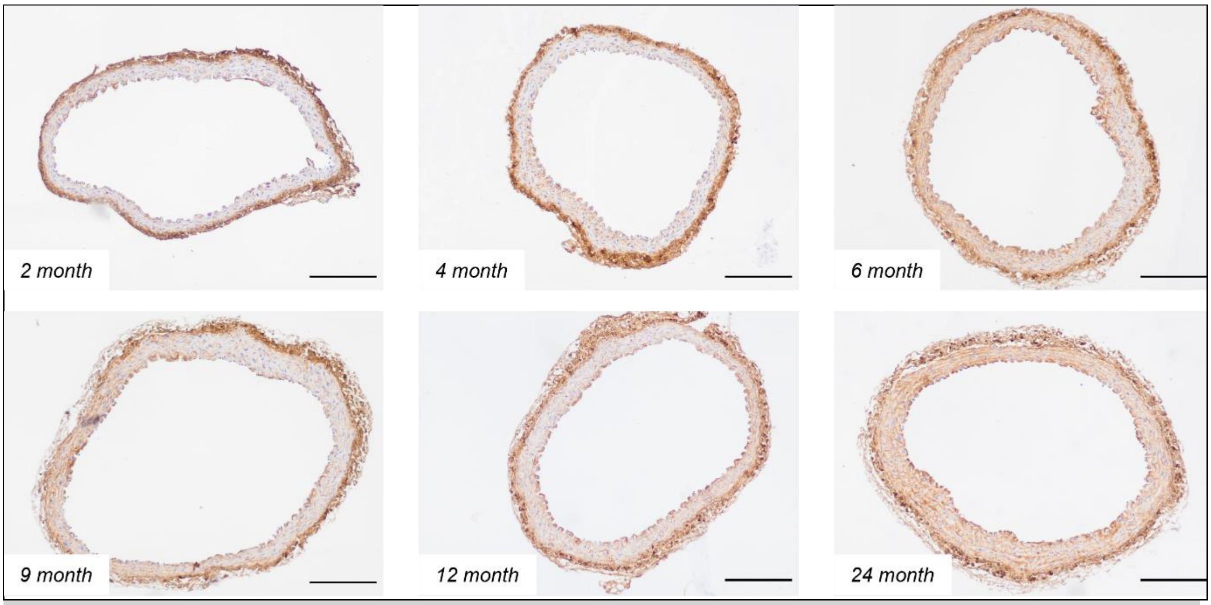

Supplementary Figure 2: Histological evaluation of collagen type I in aging C57Bl/6 mouse aortic tissue. Representative images of immunohistochemical staining for collagen type I fibres on aortic tissue of 2, 4, 6, 9, 12, and 24 month old mice. Scale bar (bottom right) represents 200 µm.

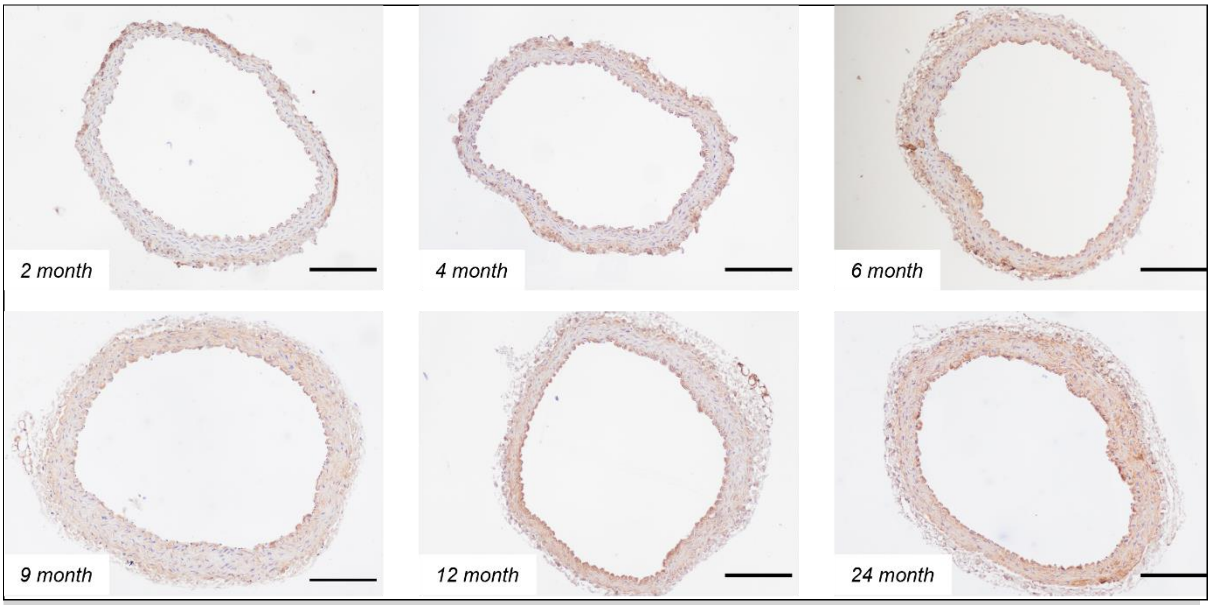

Supplementary Figure 3: Histological evaluation of collagen type III in aging C57Bl/6 mouse aortic tissue. Representative images of immunohistochemical staining for collagen type III fibres on aortic tissue of 2, 4, 6, 9, 12, and 24 month old mice. Scale bar (bottom right) represents 200 µm.

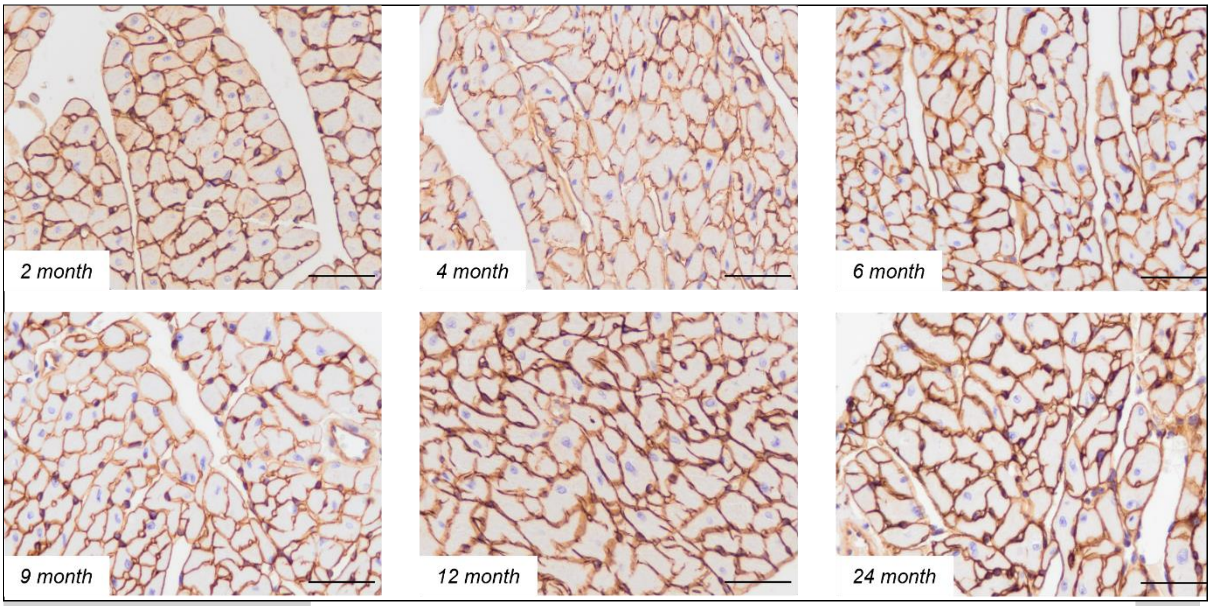

Supplementary Figure 6: Histological evaluation of cardiomyocyte cross-sectional area of aging C57Bl/6 mouse cardiac tissue. Representative images of laminin-stained cardiac tissue of 2, 4, 6, 9, 12 and 24 month old mice. **Scale bar (bottom right) represents 50 µm.**

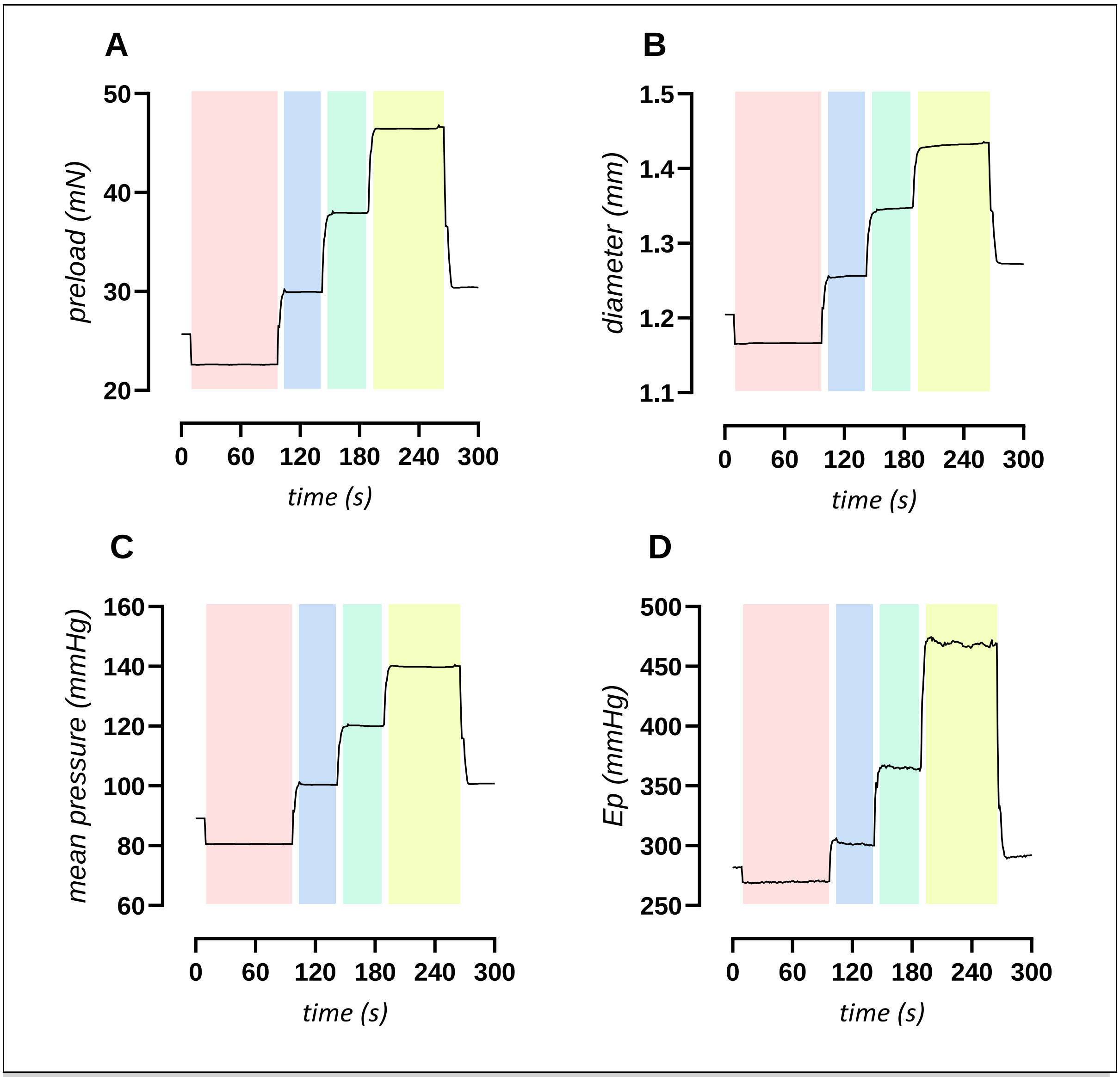

Supplementary Figure 7: Representative tracing of pressure-E_p curve recording. Measurement of force (A) and diameter (B) were used to calculate distending pressure (C) and Peterson modulus (E_p, D). Preload was adjusted to obtain 60-100 mmHg (red background), 80-120 mmHg (blue background), 100-140-mmHg (green background), and 120-160 mmHg (yellow background) pressure oscillations. On average, pressure-E_p curves were measured over a 5-minute period.

REVIEWERS' COMMENTS:

Reviewer #2 (Remarks to the Author):

I would like to thank the authors for thoroughly addressing each point of my concerns/issues from initial review. The revised manuscript is easier to follow. No further comments.

Reviewer #3 (Remarks to the Author):

The revised manuscript submitted by De Moudt et al. is markedly approved from the initial submission. The authors thoroughly responded to all comments in a thoughtful and logical manner. Furthermore, authors used important and relevant references to justify their comments in the rebuttal. Taken together, this manuscript is of high quality, has biomedical relevance, and would be a nice addition to the scientific literature on cardiovascular aging
